# A Strategy to Conjugate Bioactive Fragments to Cytotoxic Diiron Bis(cyclopentadienyl) Complexes

**DOI:** 10.1021/acs.organomet.1c00270

**Published:** 2021-07-02

**Authors:** Silvia Schoch, Mouna Hadiji, Sarah A. P. Pereira, M. Lúcia M. F. S. Saraiva, Simona Braccini, Federica Chiellini, Tarita Biver, Stefano Zacchini, Guido Pampaloni, Paul J. Dyson, Fabio Marchetti

**Affiliations:** † University of Pisa, Dipartimento di Chimica e Chimica Industriale, 56124 Pisa, Italy; ‡ Institut des Sciences et Ingénierie Chimiques, Ecole Polytechnique Fédérale de Lausanne (EPFL), Lausanne, Switzerland; § LAQV, REQUIMTE, Laboratório de Química Aplicada, Faculdade de Farmácia, da Universidade do Porto, Porto, Portugal; ∥ University of Pisa, Dipartimento di Farmacia, 56126 Pisa, Italy; ⊥ University of Bologna, Dipartimento di Chimica Industriale “Toso Montanari”, 40136 Bologna, Italy

## Abstract

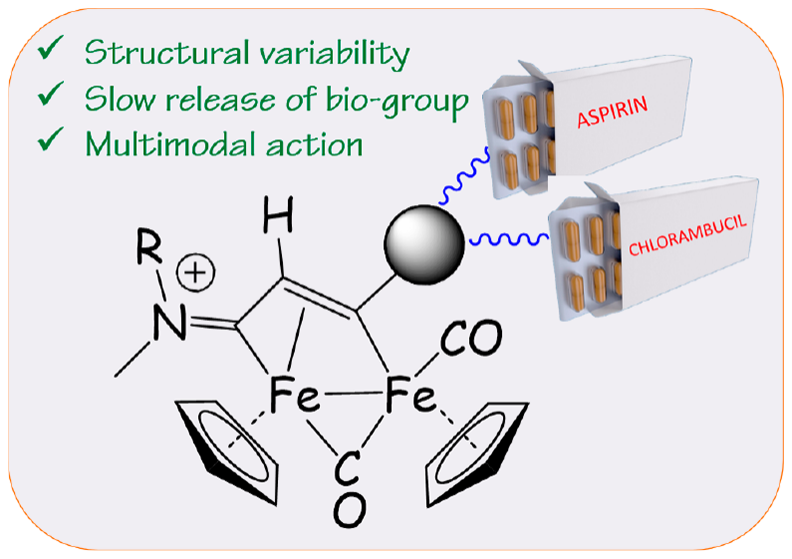

A series of bioactive molecules were synthesized from the condensation of aspirin or chlorambucil with terminal alkynes bearing alcohol or amine substituents. Insertion of the resulting alkynes into the iron–carbyne bond of readily accessible diiron bis(cyclopentadienyl) μ-aminocarbyne complexes, [**1a**,**b**]CF_3_SO_3_, afforded novel diiron complexes with a bridging vinyliminium ligand, [**2**–**10**]CF_3_SO_3_, functionalized with a bioactive moiety. All compounds were characterized by elemental analysis and IR and multinuclear NMR spectroscopy and in three cases by single-crystal X-ray diffraction. Moreover, the D_2_O solubility, stability in D_2_O and cell culture media, and octanol–water partition coefficients of diiron complexes were determined spectroscopically. The cytotoxicity of the complexes was assessed in the tumorigenic A2780 and A2780cisR and the nontumorigenic HEK 293T cell lines. Some complexes exhibit high potency and the ability to overcome resistance in A2780cisR cells (aspirin complexes) or high selectivity relative to HEK 293T cells (chlorambucil complexes). Further studies indicate that the complexes significantly trigger intracellular ROS production, irrespective of the nature of the bioactive fragment. DNA alkylation and protein binding studies were also undertaken.

## Introduction

**Figure 1:**
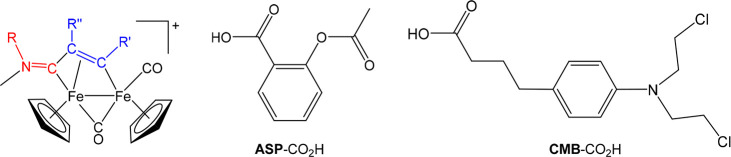
General structure of diiron μ-vinyliminium complexes obtained from the assembly of one isocyanide (fragment in red) and one alkyne (fragment in blue), starting from Fe_2_Cp_2_(CO)_4_ and structures of aspirin (**ASP**-CO_2_H) and chlorambucil (**CMB**-CO_2_H).

Platinum anticancer compounds have been extensively employed in the clinic but have important limitations due to the occurrence of severe side effects and both intrinsic and acquired resistance.^1^ Therefore, compounds based on other transition metals have been widely investigated for their anticancer potential.^2^ In this context, iron has emerged as an attractive element, being bioessential and basically nontoxic in many forms,^3^ and especially ferrocifens, resulting from the conjugation of the ferrocene skeleton with the drug tamoxifen, hold much promise.^4^ The redox chemistry of the ferrocenyl iron(II) center is the key to the cytotoxicity, with oxidation to Fe^III^ in the tumor environment triggering the production of toxic metabolites, leading to DNA damage and cell death.^5^ Additionally, some piano-stool monoiron cyclopentadienyl complexes display potent cytotoxicity against various tumor cell lines.^6^ However, the assessment of the anticancer properties of diiron complexes still remains undeveloped, despite opportunities offered by the cooperativity of two metal centers.^7^ Indeed, bimetallic systems often enable reactivity patterns not accessible to homologous monometallic compounds, with significant catalytic and biological implications.^8^ A useful diiron platform is represented by [Fe_2_Cp_2_(CO)_4_] (Cp = η^5^-C_5_H_5_), where the carbonyl ligands can be progressively replaced by small unsaturated molecules, leading to unusual organic fragments stabilized by coordination to both metal centers.^9^ In particular, bridging vinyliminium ligands can be constructed from isocyanide–alkyne coupling reactions (Figure 1).^10^ The broad availability of alkynes permits a vast choice of substituents for the vinyliminium ligand, modulating the physicochemical properties of the cationic complexes. Recently, we reported a variety of cationic diiron vinyliminium compounds that are cytotoxic against A2780 and A2780cisR cell lines, with IC_50_ values spanning from nanomolar concentrations to inactivity, depending on the substituents.^11−13^ According to preliminary studies, the mode of action seems multitargeted, involving ROS production. Antiproliferative activity is maintained in neutral derivatives containing a modified vinyliminium chain.^14^

A general and effective strategy to enhance the anticancer activity of metal complexes consists of the incorporation of an organic fragment with known biological activity, including clinically approved drugs, within the metal scaffold.^15^ Synthetic methods include direct coordination of the *bioactive group* to the *metal* and the use of a suitable ligand as a linker between such two entities.^16^ The ligand–-biomolecule connection might consist of either an ester or an amide function, and these two different linkers may significantly affect the mode of action and, hence, the cytotoxicity.^17^ The cyclopentadienyl ring(s) in ferrocenes have been extensively derivatized with bioactive molecules,^18^ e.g. ferrocifens (see above),^19^ whereas examples with non-ferrocene iron species are rare.^20^

Relevant to the present work, the inclusion of acetylsalicylic acid (aspirin) and chlorambucil (Figure 1) within Pt^IV^ and Ru^II^ anticancer complexes has resulted in synergistic effects on cancer cells originating from the two components.^21,22^ Aspirin, one of the most widely used medicines in the world, possesses analgesic, antipyretic, and anti-inflammatory properties, which are associated with the inactivation of COX-1 and COX-2 enzymes, and anticancer properties have been recently evidenced.^23^ On the other hand, chlorambucil is a DNA alkylating agent approved by the FDA for oral administration in the treatment of chronic lymphocytic leukemia and some other tumors.^22,24^

Here, we report a straightforward synthetic strategy allowing the attachment of a bioactive molecule, i.e. aspirin or chlorambucil, to a diiron scaffold which itself displays anticancer activity. This method is applicable to both ester and amide linkages. The resulting functionalized complexes have been assessed for their cytotoxicity, and experiments to elucidate mechanistic aspects have been carried out.

## Results and Discussion

### Synthesis and Characterization of Compounds

**Scheme 1:**
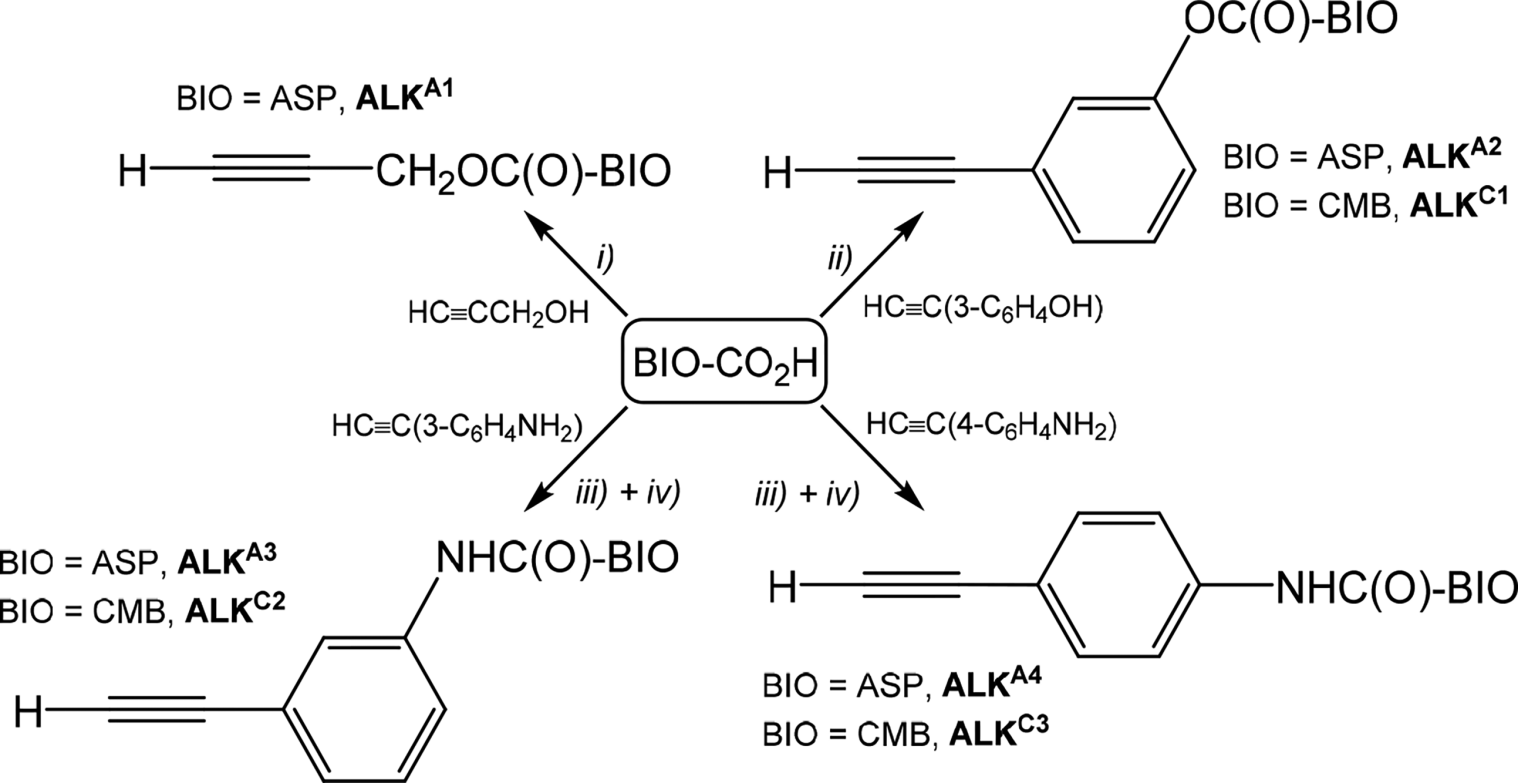
Synthesis of Terminal Alkynes Derivatized with Aspirin or Chlorambucil Reaction conditions: CH_2_Cl_2_ solution, room temperature; (i) and (ii) EDCI·HCl/DMAP; (iii) BIO-CO_2_H + oxalyl chloride/DMF; (iv) +alkyne/NEt_3_.

The bioderivatized alkynes **ALK**^**A1**^, **ALK**^**A2**^, **ALK**^**A3**^, and **ALK**^**A4**^ (aspirin-alkynes) and **ALK**^**C1**^, **ALK**^**C2**^ and **ALK**^**C3**^ (chlorambucil-alkynes) were prepared via condensation reactions of propargyl alcohol, 3-hydroxyphenylacetylene, 3-aminophenylacetylene, and 4-aminophenylacetylene with the appropriate carboxylic acid, i.e. **ASP**-CO_2_H or **CMB**-CO_2_H, using different protocols (Scheme 1). The products were purified by silica chromatography and then isolated as white/light yellow solid/oily materials in 51–95% yield.

**Figure 2:**
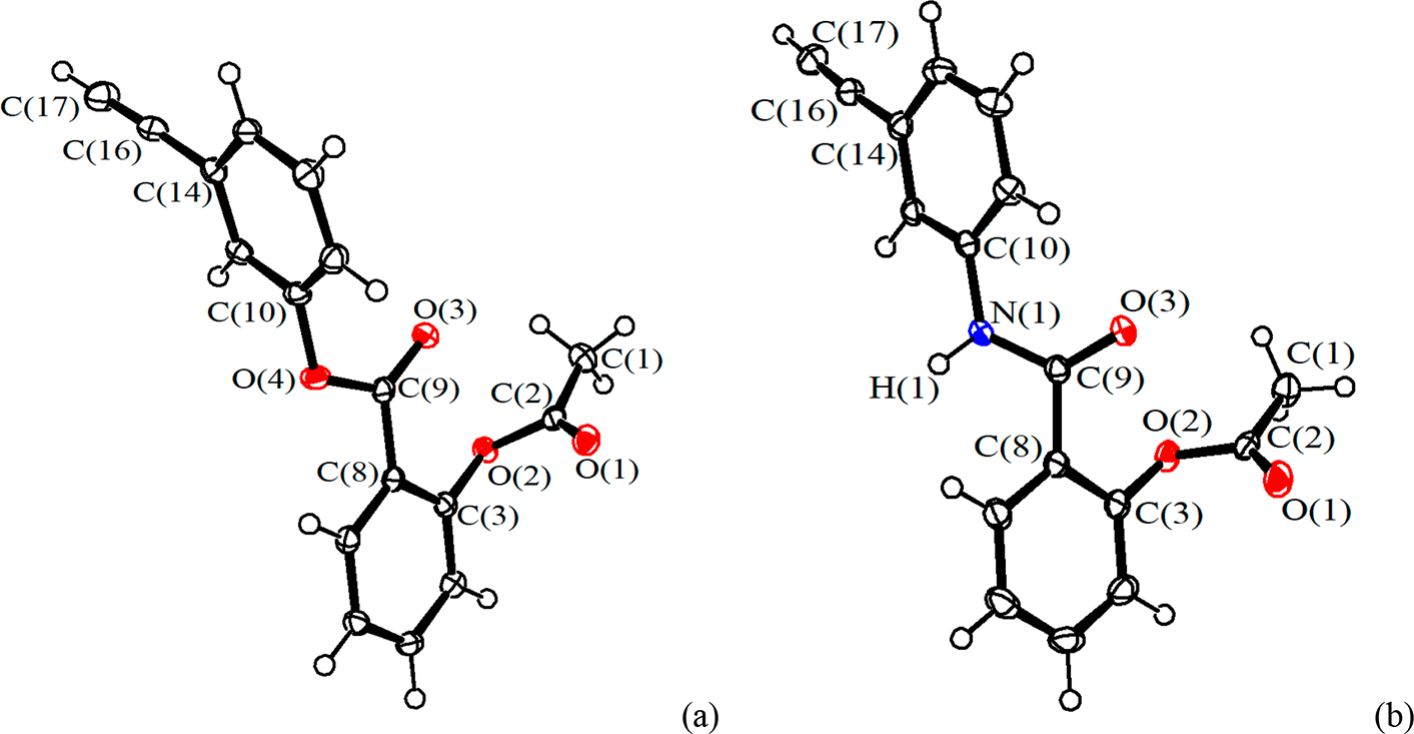
Molecular structures of (a) **ALK**^**A2**^ and (b) **ALK**^**A3**^ with key atoms labeled. Displacement ellipsoids are at the 30% probability level. Main bond distances (Å) and angles (deg) for **ALK**^**A2**^: C(1)–C(2) 1.4913(16), C(2)–O(1) 1.1947(13), C(2)–O(2) 1.3718(13), O(2)–C(3) 1.3947(13), C(8)–C(9) 1.4905(14), C(9)–O(3) 1.1984(13), C(9)–O(4) 1.3600(13), O(4)–C(10) 1.4050(13), C(14)–C(16) 1.4383(15), C(16)–C(17) 1.1840(17), C(1)–C(2)–O(2) 109.80(9), C(2)–O(2)–C(3) 116.37(8), C(8)–C(9)–O(4) 109.85(9), C(9)–O(4)–C(10) 117.57(8), C(14)–C(16)–C(17) 177.99(12). Main bond distances (Å) and angles (deg) for **ALK**^**A3**^: C(1)–C(2) 1.4827(16, C(2)–O(1) 1.2030(14), C(2)–O(2) 1.3532(13), O(2)–C(3) 1.3965(13), C(8)–C(9) 1.5035(15), C(9)–O(3) 1.2210(13), C(9)–N(1) 1.3577(14), N(1)–C(10) 1.4191(13), C(14)–C(16) 1.4400(16), C(16)–C(17) 1.1873(17), C(1)–C(2)–O(2) 110.18(9), C(2)–O(2)–C(3) 117.30(8), C(8)–C(9)–N(1) 114.60(9), C(9)–N(1)–C(10) 124.23(9), C(14)–C(16)–C(17) 175.31(12) Hydrogen bonds for **ALK**^**A3**^ (Å and deg): N(1)–H(1) 0.883(12), H(1)**···**O(1)#1 2.006(12), N(1)**···**O(1)#1 2.8653(12), ∠N(1)H(1)O(1)#1 164.0(12). Symmetry transformation: (#1) *x* + 1/2, *y*, −*z* + 1/2.

The alkyne **ALK**^**A1**^ was previously reported,^25^ while the remaining alkynes are unprecedented and were fully characterized by elemental analysis and IR and NMR spectroscopy. In the IR spectra (solid state), the triple carbon–carbon bond manifests itself by a weak absorption around 2100 cm^–1^, whereas the amide NH group in **ALK**^**A3**^, **ALK**^**A4**^, **ALK**^**C2**^, and **ALK**^**C3**^ absorbs as a medium-intensity stretching band at ca. 3250 cm^–1^. The infrared band of the carbonyl belonging to the alkyne–BIO linkage falls within the ranges 1721–1757 cm^–1^ (ester) and 1652–1673 cm^–1^ (amide). The ^1^H NMR spectra (CDCl_3_ solutions) display a resonance due to the alkyne CH proton in the range 2.56–3.56 ppm, with the NH of amide-containing species being observed between 7.35 and 9.30 ppm. In the ^13^C NMR spectra, the carbonyl groups give rise to a resonance between 162.7 and 171.8 ppm. The NMR signals due to the bioactive cores do not significantly differ from the corresponding signals of aspirin^26^ and chlorambucil.^27^ The molecular structures of **ALK**^**A2**^ and **ALK**^**A3**^ were confirmed by single-crystal X-ray diffraction (Figure 2).

**Scheme 2:**
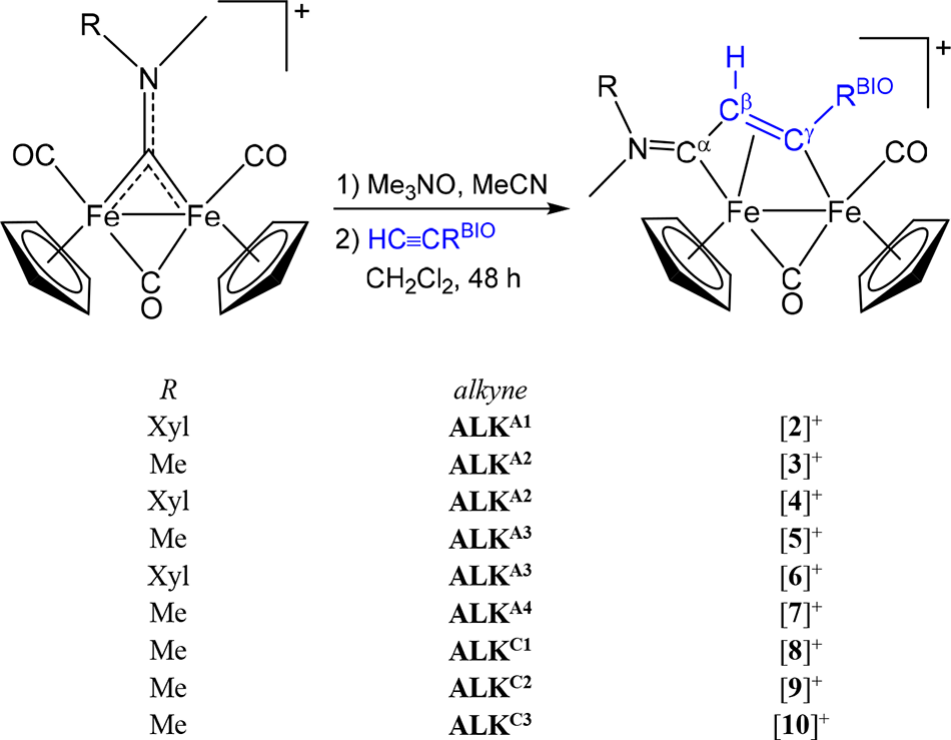
Two-Step Synthesis of Diiron Vinyliminium Complexes via Coupling of Bridging Aminocarbyne Ligands with Aspirin- and Chlorambucil-Functionalized Alkynes (CF_3_SO_3_^–^ Salts)

In order to obtain the functionalized diiron complexes, the μ-aminocarbyne complexes [Fe_2_Cp_2_(CO)_2_(μ-CO){μ-CNMe(R)}]CF_3_SO_3_ (R = Me, [**1a**]CF_3_SO_3_; R = Xyl = 2,6-C_6_H_3_Me_2_, [**1b**]CF_3_SO_3_), readily available from a multigram-scale synthesis, were first converted into the mono(acetonitrile) adducts [Fe_2_Cp_2_(CO)(NCMe)(μ-CO){μ-CNMe(R)}]CF_3_SO_3_ (R = Me, Xyl).^28^ Then, the latter complexes, in dichloromethane solution, were treated with a slight molar excess of the alkyne, thus allowing a highly regio- and stereoselective alkyne insertion into the iron–bridging carbyne bond upon removal of the labile acetonitrile ligand (Scheme 2).

Complexes [**2**–**10**]CF_3_SO_3_ were isolated in 48–96% yield after workup and fully characterized by elemental analysis and IR and NMR spectroscopy. The IR spectra (CH_2_Cl_2_ solutions) share a common pattern with two bands due to terminal (1991–2007 cm^–1^) and bridging carbonyl ligands (1807–1820 cm^–1^) and another band related to the {C_β_C_α_N}^10a^ moiety (1628–1692 cm^–1^). The ester linkages between the vinyliminium and the bioactive fragment in [**2**–**4**,**8**]CF_3_SO_3_ absorb in the 1727–1758 cm^–1^ range, whereas the amido carbonyl groups belonging to [**5**–**7**,**9**–**10**]CF_3_SO_3_ are observed at 1678–1691 cm^–1^. The ^1^H NMR spectra of [**2**,**6**]CF_3_SO_3_ (acetone-*d*_6_ solutions) contain two sets of resonances, due to *E*–*Z* isomerism arising from the different substituents on the iminium nitrogen, with prevalence of the *E* form (*E*/*Z* ratio = 2 for [**2**]CF_3_SO_3_ and 9 for [**6**]CF_3_SO_3_). The other complexes exist as single isomeric species, including [**4**]CF_3_SO_3_, which is present in solution as the *E* isomer only. Comparisons with a library of data related to nonfunctionalized diiron vinyliminium complexes indicate that the Cp ligands exclusively adopt a mutual *cis* geometry in all compounds [**2**–**10**]CF_3_SO_3_.^10−12,29^ C_β_-H resonates at 4.4–4.8 ppm and is negligibly affected by the adjacent C_γ_ substituent; the NH amide proton in [**5**–**7**,**9**–**10**]CF_3_SO_3_ is slightly deshielded in comparison to the alkyne precursors and is observed at around 9.5 ppm. Salient ^13^C NMR features are given by the resonances related to the vinyliminium carbon chain, respectively in the intervals 224.3–232.4 ppm (C_α_), 48.6–53.8 ppm (C_β_), and 200.7–207.8 ppm (C_γ_), thus evidencing the alkylidene character of C_α_ (amino-alkylidene) and C_γ_. The chemical shifts for the ester/amide linkage carbon in [**2**–**10**]CF_3_SO_3_ do not differ significantly with respect to the biofunctionalized alkynes.

**Figure 3:**
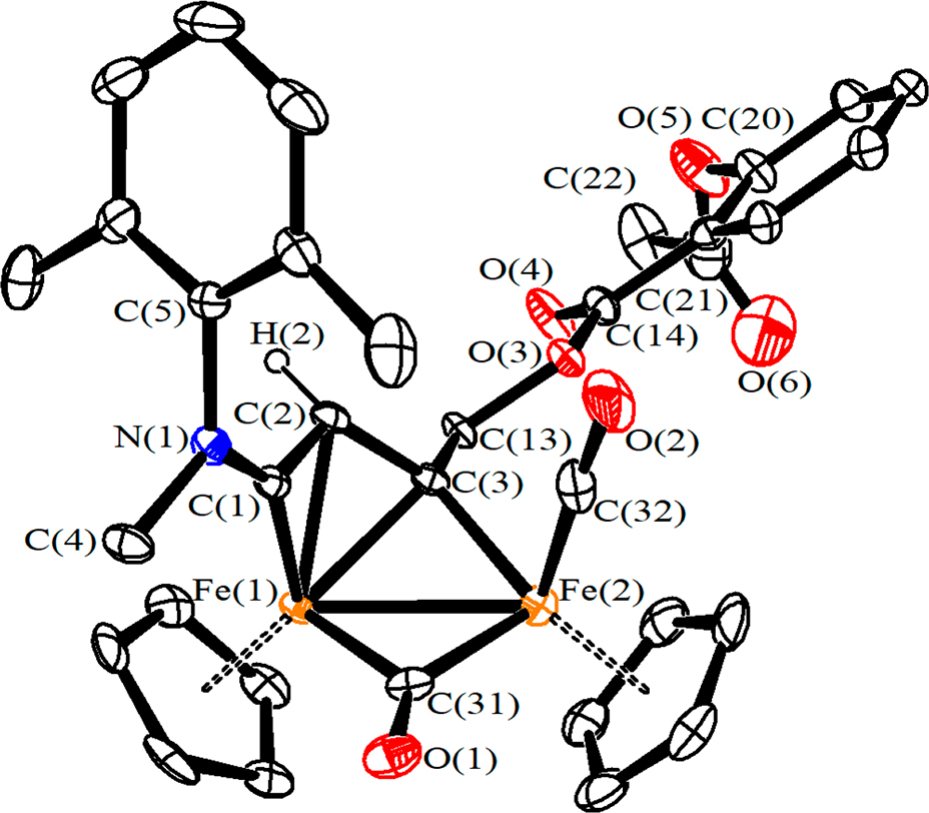
View of the cation of [**2**]CF_3_SO_3_ with key atoms labeled. Displacement ellipsoids are at the 30% probability level. Hydrogen atoms, except H(2), have been omitted for clarity. Main bond distances (Å) and angles (deg): Fe(1)–Fe(2) 2.5421(12), Fe(1)–C(31) 1.971(6), Fe(2)–C(31) 1.876(6), Fe(2)–C(32) 1.758(8), Fe(1)–C(3) 2.016(6), Fe(2)–C(3) 1.945(6), Fe(1)–C(2) 2.042(6), Fe(1)–C(1) 1.831(6), C(31)–O(1) 1.172(8), C(32)–O(2) 1.153(9), C(1)–N(1) 1.295(8), C(1)–C(2) 1.411(8), C(2)–C(3) 1.416(9), C(3)–C(13) 1.511(8), C(13)–O(3) 1.446(7), O(3)–C(14) 1.326(7), C(14)–O(4) 1.210(8), Fe(1)–C(31)–Fe(2) 82.7(2), Fe(1)–C(3)–Fe(2) 79.8(2), Fe(2)–C(32)–O(2) 175.5(7), Fe(2)–C(3)–C(2) 122.2(4), C(3)–C(2)–C(1) 116.6(5), C(2)–C(1)–N(1) 134.5(6), C(3)–C(13)–O(3) 105.1(5), C(13)–O(3)–C(14) 116.1(5), O(3)–C(14)–O(4) 123.1(6).

The structure of [**2**]CF_3_SO_3_ was elucidated by X-ray diffraction (Figure 3). The cation is composed of a [Fe_2_Cp_2_(CO)(μ-CO)] skeleton, with the Cp-ligands in a *cis* geometry, and a {μ-η^1^:η^3^-C^3^(R)C^2^HC^1^N(Me)(Xyl)}^+^ vinyliminium ligand bearing the aspirin moiety (R = CH_2_OC(O)C_6_H_4_OC(O)CH_3_). The bridging alkylidene C^3^ carbon is slightly asymmetric with respect to the Fe centers (Fe(1)–C(3) 2.016(6) Å, Fe(2)–C(3) 1.945(6) Å), and a more pronounced asymmetry is observed for the μ-CO ligand (Fe(1)–C(31) 1.971(6) Å, Fe(2)–C(31) 1.876(6) Å). The C(1)–N(1) distance (1.295(8) Å) is indicative of an iminium bond, although the Fe(1)–C(1) distance (1.831(6) Å) possesses some aminoalkylidene nature, in agreement with the ^13^C NMR spectrum.^10,11^ The iminium group adopts an *E* conformation which corresponds to the prevalent conformation detected in solution by NMR spectroscopy (see above).

### Solubility and Stability in Aqueous Media and Octanol–Water Partition Coefficients

The solubility of the complexes in D_2_O was assessed using ^1^H NMR spectroscopy (see Table 1 and the Experimental Section for details). Complexes [**3**]CF_3_SO_3_, [**5**]CF_3_SO_3_, and [**7**]CF_3_SO_3_ possess appreciable water solubility, whereas the solubility values for the remaining compounds are low. Data from the literature for the related nonfunctionalized complexes [**2′,3′**]CF_3_SO_3_ are compiled in Table 1 for comparison.^11^ The octanol–water partition coefficients (Log *P*_ow_) were measured using a UV–vis method and fall within the −0.2 to +1.2 range (Table 1). The structural variability provided by the synthetic route offers much opportunity for fine-tuning the lipophilicity of the complexes, which is significantly influenced by the iminium substituents, the type of linkage to the bioactive group (i.e., ester or amide), and its location on the aryl ring. For instance, Log *P*_ow_ values of 1.07 and 0.65 are obtained for the geometric isomers [**9**]CF_3_SO_3_ and [**10**]CF_3_SO_3_, respectively.

**Table 1:** Solubility in D_2_O (based on ^1^H NMR Spectroscopy, Me_2_SO_2_ as Internal Standard) and Partition Coefficients (Log *P*_ow_) of Diiron Complexes (*T* = 21 °C) and Residual Percent of cComplex in D_2_O/DMSO 2/1 v/v Mixture after 24 h at 37 °C

compound	solubility (mol L^–1^)	Log *P*_ow_	stability (%)
[**2**]CF_3_SO_3_	<1 × 10^–4^	0.40 ± 0.01	85 (69)^a^
[**3**]CF_3_SO_3_	7.8 × 10^–4^	0.24 ± 0.01	81 (57)^a^
[**4**]CF_3_SO_3_	<1 × 10^–4^	1.06 ± 0.04	59
[**5**]CF_3_SO_3_	1.1 × 10^–3^	–0.10 ± 0.01	69
[**6**]CF_3_SO_3_	<1 × 10^–4^	0.80 ± 0.02	91
[**7**]CF_3_SO_3_	5.3 × 10^–4^	–0.19 ± 0.01	70
[**8**]CF_3_SO_3_	<1 × 10^–4^	1.2 ± 0.2	84 (77)^a^^,^^b^
[**9**]CF_3_SO_3_	<1 × 10^–4^	1.07 ± 0.05	88^b^
[**10**]CF_3_SO_3_	<1 × 10^–4^	0.65 ± 0.02	93 (85)^a^^,^^b^
[**2′**]CF_3_SO_3_^c^	1.0 × 10^–3^	–0.1	
[**3′**]CF_3_SO_3_^c^	1.4 × 10^–2^	–0.34	

a The residual percentage of the complex after 72 h at 37 °C is given in parentheses.

b Total percentage of diiron complexes with a chlorambucil-like group in D_2_O/DMSO 1/1 v/v mixture.

c Values from ref (11).

**Figure 4:**
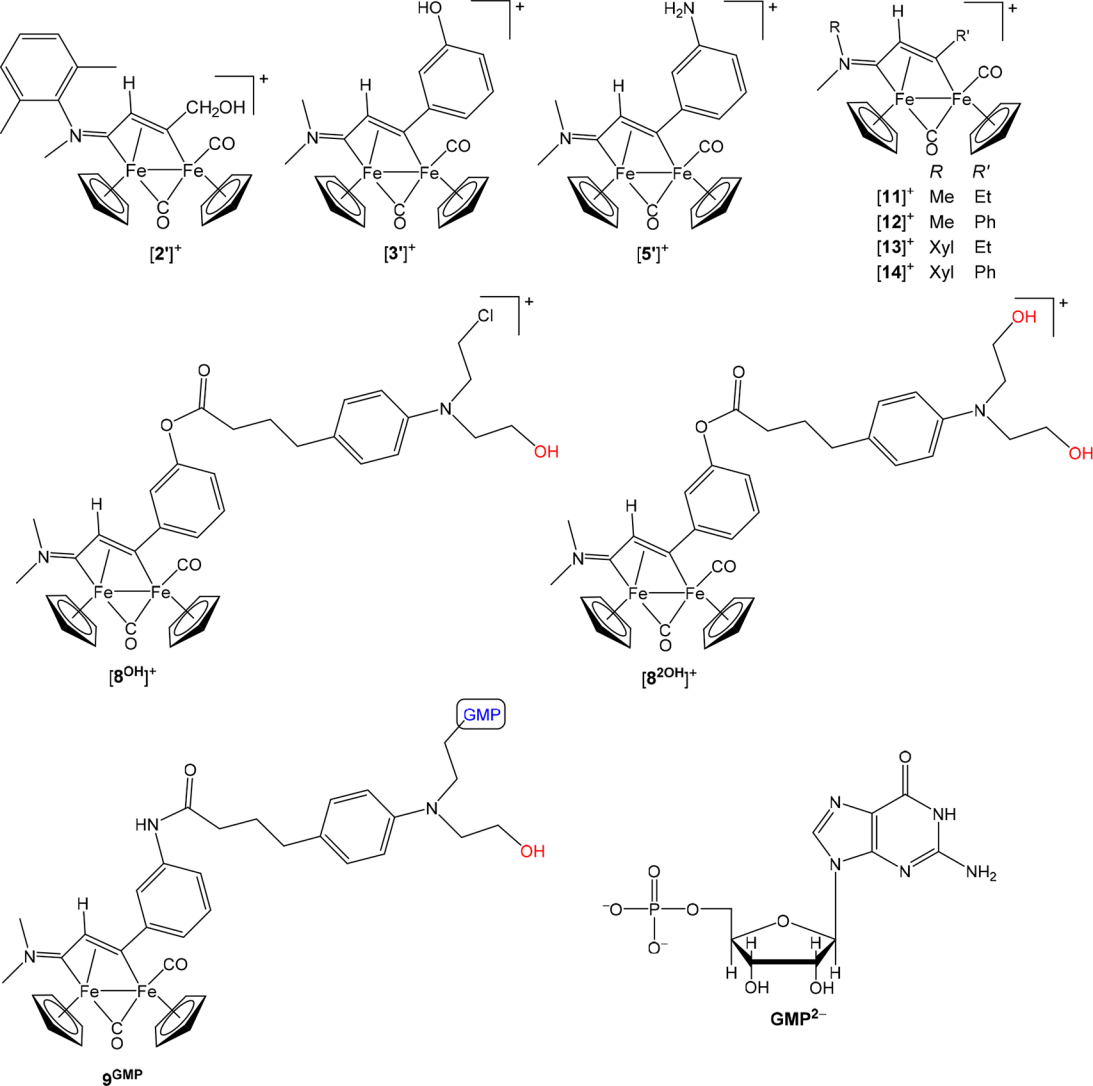
Structures of diiron complexes discussed in this work in addition to those shown in Scheme 2: [**2’**]^+^, [**3′**]^+^, [**5′**]^+^, [**8**^**OH**^]^+^, and [**8**^**2OH**^]^+^ formed upon release of the bioactive fragments in aqueous/biological media; **9**^**GMP**^ formed upon interaction of [**9**]^+^ with the model nucleotide guanosine 5′-monophosphate (disodium salt, Na_2_[GMP]); [**11**–**14**]^+^ investigated for COX-2 inhibition assays. The structures of [**8**^**OH**^]^+^ and [**8**^**2OH**^]^+^ are also representative of those of the homologous complexes [**9**^**OH**^]^+^, [**10**^**OH**^]^+^, [**9**^**2OH**^]^+^, and [**10**^**2OH**^]^+^ (not shown).

Stability studies in deuterated aqueous medium at 37 °C were carried out using ^1^H NMR spectroscopy and evidenced partial release (approximately 20%) of aspirin (Asp-CO_2_H) from the {CH_2_–OCOAsp} linkage within [**2**]CF_3_SO_3_ after 24 h (see Figure S40 in the Supporting Information), to give the hydrolyzed alcohol derivative [**2′**]^+^ (Figure 4). Conversely, [**3**–**7**]CF_3_SO_3_, containing either {Aryl–OCOAsp} or {Aryl-NHCOAsp} moieties, were stable under the same conditions. The ester bond connecting chlorambucil to the aryl unit in [**8**]CF_3_SO_3_ did not undergo hydrolysis; nevertheless, gradual chloride/hydroxide exchange occurred within the peripheral chlorambucil moiety, affording [**8**^**OH**^]^+^ and [**8**^**2OH**^]^+^ (Figure 4; their identity was confirmed by HPLC-MS). Complete conversion into the bis-hydroxide [**8**^**2OH**^]^+^ was achieved within 72 h. Similarly, [**9**,**10**]CF_3_SO_3_ undergo activation of the {C–Cl} bonds to give mixtures of the partially Cl/OH substituted species [**9**^**OH**^]^+^ and [**10**^**OH**^]^+^ and the fully substituted species [**9**^**2OH**^]^+^ and [**10**^**2OH**^]^+^.

Complexes [**3**]CF_3_SO_3_, [**5**]CF_3_SO_3_, and [**8**]CF_3_SO_3_ were selected for an evaluation of their stability in the culture medium. Compounds were dissolved in DMSO-*d*_6_, and these solutions were diluted with the cell culture medium and stored at 37 °C. According to NMR spectroscopy, progressive release of the bioactive fragment occurred over 72 h, affording [**3′**]^+^ from [**3**]CF_3_SO_3_ and [**5′**]^+^ from [**5**]CF_3_SO_3_ (Figures S41 and S42). The amide linkage between aspirin and the diiron frame in [**5**]CF_3_SO_3_ was found to be weaker than the corresponding ester bond in [**3**]CF_3_SO_3_, and complete dissociation was observed after 72 h for the former complex. The ^1^H NMR spectrum suggests that chlorambucil release from [**8**]CF_3_SO_3_ is accompanied by the gradual hydrolysis of the two chloroethyl groups, as observed in D_2_O/DMSO-*d*_6_. Overall, these findings indicate that the diiron scaffold decorated with biofunctionalized vinyliminium ligands is robust in aqueous media, with a tendency to progressively release the bioactive moiety in the medium used in cytotoxicity studies.

### Cytotoxicity Studies

The antiproliferative activity of the new diiron complexes [**2**–**10**]CF_3_SO_3_ and that of cisplatin as a reference was assessed toward cisplatin-sensitive and cisplatin-resistant human ovarian cancer cell lines, A2780 and A2780cisR, respectively, and the noncancerous HEK 293T cell line. The results are compiled in Table 2, where they are compared to those previously reported for [**2′,3′**]CF_3_SO_3_,^11^ [**1a**,**b**]CF_3_SO_3_,^7^ chlorambucil,^22b^ and aspirin.^30^ In general, a correlation exists between the IC_50_ and Log *P*_ow_ values, the most lipophilic compounds being also the most active. With regard to the aspirin-containing complexes [**2**–**7**]CF_3_SO_3_, these exhibit a comparable level of activity against the two cancer cell lines; in particular, [**4**]^+^ and [**6**]^+^ are strongly cytotoxic with IC_50_ values falling in the low-micromolar range. The performance of the chlorambucil derivative [**9**]CF_3_SO_3_ is notable, since this complex displays a potent cytotoxicity against the A2780 cell line, a significant selectivity (SI = 5), and an enhanced ability to overcoming cisplatin resistance (IC_50_ on A2780cisR cell line 7.9 μM for cisplatin, 3.8 μM for [**9**]CF_3_SO_3_). The performance of [**9**]CF_3_SO_3_ against the cancer cell lines is superior to that of chlorambucil. The chlorambucil complex [**8**]CF_3_SO_3_, differing from [**9**]CF_3_SO_3_ in the presence of an ester group instead of an amide, also displays some selectivity, but it is not as effective as the aspirin complexes in the cisplatin-resistant cell line. Overall, the attachment of bioactive fragments to the diiron scaffold leads to a marked effect on the activity of the complexes. Finally, it should be mentioned that the cytotoxicity of the vinyliminium-functionalized complexes [**3**,**5**,**7**–**10**]CF_3_SO_3_ on the A2780 cell line is strongly improved in comparison to their aminocarbyne precursor [**1a**]CF_3_SO_3_, which is not active;^7^ a previous investigation pointed out the absence of activity of the **ALK**^**A1**^ toward cancer cell lines.^25^

**Table 2:** IC_50_ Calues (μM) Determined for Compounds [**2**–**10**]CF_3_SO_3_, Cisplatin, [**2′**,**3′**]CF_3_SO_3_,^11^ [**1a**,**b**]CF_3_SO_3_,^7^ Aspirin,^30^ and Chlorambucil^22b^ on Human Ovarian Carcinoma (A2780), Human Ovarian Carcinoma Cisplatin Resistant (A2780cisR), and Human Embryonic Kidney 293T (HEK 293T) Cell Lines after 72 h Exposure^a^

compound	A2780	A2780cisR	HEK 293T
[**2**]CF_3_SO_3_	13 ± 2	14 ± 1	9.2 ± 0.5
[**3**]CF_3_SO_3_	24 ± 5	21 ± 5	42 ± 6
[**4**]CF_3_SO_3_	2.8 ± 0.4	2.6 ± 0.4	3.8 ± 0.7
[**5**]CF_3_SO_3_	43 ± 6	45 ± 1	89.9 ± 0.3
[**6**]CF_3_SO_3_	6.5 ± 0.8	7 ± 2	7.2 ± 0.9
[**7**]CF_3_SO_3_	71 ± 14	87 ± 10	>100
[**8**]CF_3_SO_3_	4.7 ± 0.4	23 ± 3	20 ± 9
[**9**]CF_3_SO_3_	1.8 ± 0.3	3.8 ± 0.3	9 ± 3
[**10**]CF_3_SO_3_	6.4 ± 1.4	15 ± 5	30 ± 1
[**2′**]CF_3_SO_3_	11.6 ± 0.6	21 ± 2	13.4 ± 1.0
[**3′**]CF_3_SO_3_	163 ± 16	172 ± 11	200 ± 21
[**1a**]CF_3_SO_3_	>200		
[**1b**]CF_3_SO_3_	9.3 ± 0.7		
aspirin	>200	>200	>200
chlorambucil	5.2 ± 1.6	25 ± 4	
cisplatin	0.6 ± 0.1	7.9 ± 0.1	2.6 ± 0.4

a Values are given as the mean ± SD.

### Mechanistic Studies

**Figure 5:**
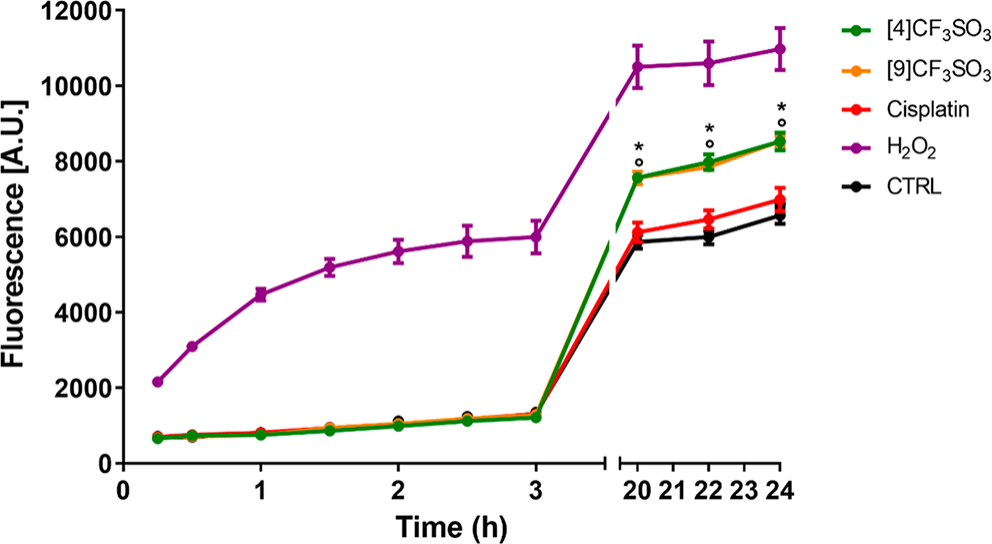
Fluorescence kinetic measurements of intracellular reactive oxygen species (ROS, *p* < 0.05). A2780 cells were incubated for 24 h with 10 μM of the iron compounds at 37 °C and 5% CO_2_.

In order to shed light on the possible mechanism of action of the biofunctionalized diiron complexes, a series of complementary studies were performed. It was previously shown that the diiron vinyliminium structural motif induces ROS production, possibly associated with more than one mechanism: (1) monoelectron reduction of the complex favored by its net cationic charge; (2) fragmentation into a monoiron derivative and atomic iron; (3) rupture of the organometallic scaffold in aqueous medium releasing the two Fe^+I^ centers which rapidly convert into iron(III) oxides.^11−13,7^ The aspirin complex [**4**]CF_3_SO_3_ and chlorambucil complex [**9**]CF_3_SO_3_ were selected as representative, strongly cytotoxic compounds for the evaluation of induced intracellular ROS production. Thus, fluorescence measurements were conducted using the DCFH-DA assay, exposing A2780 cells to [**4**]CF_3_SO_3_, [**9**]CF_3_SO_3_, the reference drug cisplatin, or H_2_O_2_ as a positive control. Significant intracellular ROS levels were detected from [**4**]CF_3_SO_3_ and [**9**]CF_3_SO_3_, especially after ca. 20 h, and progressively increasing up to 24 h (Figure 5). Remarkably, both diiron complexes elicited a ROS production higher than that recorded for H_2_O_2_. Thus, the incorporation of the bioactive fragments within the diiron structure does not reduce the ability of the complexes to interfere with cellular redox processes via ROS production, a phenomenon which may substantially contribute to the observed antiproliferative activity.

Next, the possible role of the bioactive fragment in the complexes was studied. COX-2 enzyme inhibition assays were performed on the aspirin compounds [**2**–**7**]CF_3_SO_3_ and also on a series of nonfunctionalized vinyliminium complexes as references, [**11**–**14**]CF_3_SO_3_ (Figure 4). In each case, the enzyme was treated with the complex and the residual enzyme activity was measured to determine the IC_50_ concentrations (Table 3). All diiron–aspirin conjugates exhibit significantly lower IC_50_ values in comparison to [**11**–**14**]CF_3_SO_3_ and aspirin itself; this outcome indicates that the assembly of aspirin with the diiron framework provides a synergic effect in terms of COX-2 inhibition.

**Table 3:** IC_50_ Values (μM) Determined for Diiron Complexes and Aspirin in the Inhibition of COX-2 Enzyme^a^

compound	IC_50_ value (μM)
[**2**]CF_3_SO_3_	84 ± 2
[**3**]CF_3_SO_3_	71 ± 1
[**4**]CF_3_SO_3_	30 ± 3
[**5**]CF_3_SO_3_	10 ± 2
[**6**]CF_3_SO_3_	66 ± 2
[**7**]CF_3_SO_3_	20 ± 2
[**11**]CF_3_SO_3_	541 ± 55
[**12**]CF_3_SO_3_	506 ± 15
[**13**]CF_3_SO_3_	629 ± 49
[**14**]CF_3_SO_3_	288 ± 6
aspirin	>1500^31^

a Values are given as the mean ± SD.

**Figure 6:**
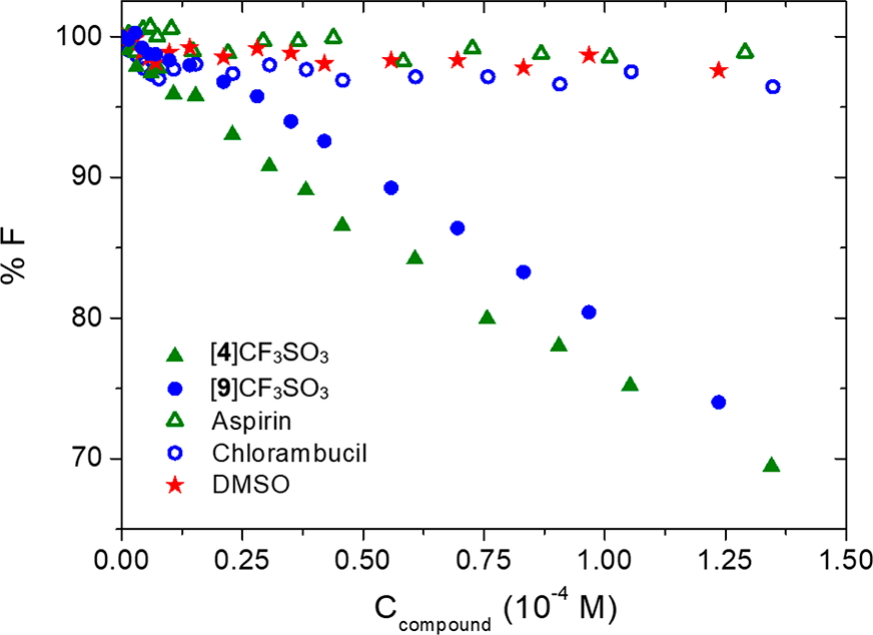
Ethidium bromide displacement tests for selected diiron vinyliminium complexes. Conditions: *C*_DNA_ = 1.15 × 10^–4^ M, *C*_EB_ = 5.60 × 10^–5^ M, NaCl 0.1 M, NaCac 0.01 M, λ_ex_ = 520 nm, λ_em_= 595 nm, *T* = 25 °C.

The ability of [**4**]CF_3_SO_3_ and [**9**]CF_3_SO_3_ to interact with natural DNA was studied using the ethidium bromide (EB) exchange test (Figure 6), with the concentration of the complexes beingin the 1.5–124 μM range for [**4**]CF_3_SO_3_ and 1.4–135 μM for [**9**]CF_3_SO_3_. Blank experiments were carried out with the two bioactive molecules and also with DMSO to check for solvent/dilution effects. The results indicate that both [**4**]CF_3_SO_3_ and [**9**]CF_3_SO_3_ are able to interact weakly with natural DNA, similarly to that previously described for nonfunctionalized diiron vinyliminium complexes.^11^ However, EB exchange titrations are suitable to recognize fast noncovalent binding events, but not subsequent slow covalent binding. Therefore, since chlorambucil is a DNA-alkylating agent, we studied the possible reaction of [**9**]CF_3_SO_3_ with the nucleotide guanosine 5′-monophosphate (disodium salt, Na_2_[GMP]; see Figure 4) as a model for DNA binding. Complex [**9**]CF_3_SO_3_ was incubated with a D_2_O/CD_3_OD mixture containing GMP for 24 h at 37 °C. A subsequent mass spectrometric analysis was indicative of almost complete conversion of [**9**]^+^ into the neutral adduct **9**^**GMP**^ (Figure 4); the latter is best viewed as the result of the modification of the two ethyl chloride functions of [**9**]^+^, one of the two being hydrolyzed and the other one undergoing chloride substitution by one imine function of [GMP]^2–^.

The interaction of [**4**]CF_3_SO_3_ and [**9**]CF_3_SO_3_ with bovine serum albumin (BSA) as a model protein was also explored. The addition of the metal complex to the protein in pH buffer solution (pH 7.0 buffer; NaCl 0.1 M, NaCac 0.01 M) produced a quenching of the intrinsic fluorescence emission of the latter. A data fit, according to the Stern–Volmer equation,^11^ provided quenching constants (*K*_SV_) values equal to ca. 3.4 × 10^4^ for both systems, indicative that a binding event occurs (Figure S1).^32^ Spectral analysis^33^ collected during the titration revealed that a 1:1 stoichiometry model is adequate to describe the binding (Figure S2). The related Log *K* values are 5.9 ± 0.1 for [**4**]CF_3_SO_3_/BSA and 6.5 ± 0.1 for [**9**]CF_3_SO_3_/BSA and suggest the occurrence of a reversible interaction, similarly to that previously found for nonfunctionalized diiron vinyliminium complexes.^11^ More precisely, the binding constant (10^6^) appears to be high enough to ensure adduct formation but also weak enough to release the complex once the biotarget is reached; this is assumed to be the optimal condition for BSA-driven transport and diffusion of a drug.^34^

## Conclusions

Monoiron cyclopentadienyl complexes, and ferrocenes in particular, have aroused great interest due to their anticancer properties, while diiron complexes have been much less investigated despite the advantageous cooperative effects provided by two iron centers. The readily accessible {Fe_2_Cp_2_(CO)_2_} scaffold offers considerable opportunities for the construction of various bridging hydrocarbyl ligands, and here we show that bioactive carboxylic acids can be incorporated through an alkyne-insertion reaction. Aspirin (enzyme inhibitor) and chlorambucil (DNA alkylating agent) were selected as representative compounds to demonstrate the viability of the synthetic approach, but this may be extended to other bioactive molecules given the generality of the insertion reaction.^10−12,29^ The new complexes exhibit favorable characteristics in aqueous media, in that they are quite stable with a tendency to progressively release the bioactive fragment. The cytotoxicity ranges from moderate to the low-micromolar range, and complementary experiments reveal a possible multimodal action of the complexes, with the induction of intracellular ROS production playing a major role. The complexes manifest the specificity provided by the bioactive fragment, since experiments reveal the ability of chlorambucil complexes to alkylate DNA and that of aspirin complexes to inhibit COX-2 enzyme. In particular, diiron–chlorambucil conjugates display a cytotoxicity profile more comparable to that of cisplatin. The versatility of the synthetic method and the broad structural variability provided by the diiron core allows fine-tuning of physicochemical properties of the complexes, which is conducive to the future development of optimal iron drug candidates.

## Experimental Section

### Synthesis and Characterization of Compounds

#### General Details

Organic reactants were purchased from TCI Europe or Merck and were of the highest purity available, while solvents were purchased from Merck (petroleum ether, bp 40–60 °C). The compounds **ALK**^**A1**^,^25,35^ [Fe_2_Cp_2_(CO)_2_(μ-CO){μ-CNMe(R)}]CF_3_SO_3_ (R = Me, [**1a**]CF_3_SO_3_; R = Xyl = 2,6-C_6_H_3_Me_2_, [**1b**]CF_3_SO_3_),^28^ [**11**]CF_3_SO_3_,^36^ [**12**]CF_3_SO_3_, and [**14**]CF_3_SO_3_^11^ and [**13**]CF_3_SO_3_^37^ were prepared according to the literature. The synthesis of alkynes was carried out under an N_2_ atmosphere using standard Schlenk techniques, and solvents were distilled before use from the appropriate drying agents under N_2_; all other operations were conducted in air. Once isolated, products were stored in air. Separations were carried out on columns of silica (Merck), deactivated alumina (Merck, 4% w/w water), or Celite (Fluka, 512 Medium). Infrared spectra of solutions were recorded on a PerkinElmer Spectrum 100 FT-IR spectrometer with a CaF_2_ liquid transmission cell (2300–1500 cm^–1^ range) or on solid samples at 298 K on a PerkinElmer FT-IR spectrometer, equipped with a UATR sampling accessory. UV–vis spectra were recorded on an Ultraspec 2100 Pro spectrophotometer. IR and UV–vis spectra were processed with Spectragryph software.^38^ NMR spectra were recorded at 298 K on a Bruker Avance II DRX400 instrument equipped with a BBFO broad-band probe. Chemical shifts (expressed in parts per million) are referenced to the residual solvent peaks^39^ (^1^H, ^13^C). NMR spectra were assigned with the assistance of ^1^H–^13^C (*gs*-HSQC and *gs*-HMBC) correlation experiments.^40^ NMR signals due to secondary isomeric forms (where it has been possible to detect them) are italicized. Elemental analyses were performed on a Vario MICRO cube instrument (Elementar). HPLC-MS analyses were performed with a HPLC 1200 Infinity, coupled with a quadrupole time of flight tandem mass spectrometer 6530 Infinity Q-ToF detector by a Jet Stream ESI interface (Agilent Technologies, USA); the data were processed with Mass Hunter Qualitative Analysis software.

### Synthesis and Characterization of Diiron Complexes

#### General Procedure

A solution of [**1a**,**b**]CF_3_SO_3_ (ca. 0.5 mmol) in MeCN (ca. 10 mL) was treated with Me_3_NO (1.3 equiv). The resulting mixture was stirred for 1 h, during which time progressive color darkening occurred. The complete conversion of the starting material into the corresponding acetonitrile adduct [Fe_2_Cp_2_(CO)(μ-CO)(NCMe){μ-CN(Me)(R)}]CF_3_SO_3_^41^ was checked by IR spectroscopy. The volatiles were removed under vacuum to afford a dark brown residue that was dissolved in dichloromethane (ca. 20 mL) and treated with the appropriate alkyne (ca. 1.3 equiv). The mixture was stirred at room temperature for 48 h, and then it was filtered through Celite. The volatiles were evaporated from the filtered solution under reduced pressure, and the residue was repeatedly washed with diethyl ether and finally dried under vacuum.

##### [Fe_2_Cp_2_(CO)(μ-CO){μ-η^1^:η^3^-C_γ_(CH_2_OC(=O)C_6_H_4_OC(=O)Me)C_β_HC_α_NMe(Xyl)}]CF_3_SO_3_ ([**2**]CF_3_SO_3_) (Chart 1)

This compound was obtained from [**1b**]CF_3_SO_3_ and **ALK**^**A1**^: brown solid, yield 52%. Anal. Calcd for C_35_H_32_F_3_Fe_2_NO_9_S: C, 51.81; H, 3.98; N, 1.73; S, 3.95. Found: C, 51.70; H, 3.88; N, 1.83; S, 4.04. IR (CH_2_Cl_2_): ν̃/cm^–1^ 2007vs (CO), 1820s (μ-CO), 1766s (Me*C=O*), 1727s (C_6_H_4_*C=O*), 1634m (C_β_C_α_N), 1607m (C–C_arom_). ^1^H NMR (CDCl_3_): δ/ppm 8.05–6.90 (m, 7 H, C_6_H_4_ + C_6_H_3_); 6.81, 6.53 (d, ^2^*J* = 15.1 Hz, 2 H, CH_2_); 5.53, 5.24, 5.03, 4.45 (s, 10 H, Cp); 4.38 (s, 1 H, C_β_H); 4.20, 2.70 (s, 3 H, NMe); 2.21, 2.20 (s, 3 H, O=CMe); 2.18, 2.14, 2.07, 1.80 (s, 6 H, C_6_H_3_*Me*_2_). *E*/*Z* ratio ca. 2. ^13^C{^1^H} NMR (acetone-*d*_6_): δ/ppm 252.7 (μ-CO); 232.4 (C_α_); 210.0 (CO); 200.7 (C_γ_); 169.0 (O=*C*Me); 163.5 (C_6_H_4_*C*=O); 151.1 (C^2^); 145.2, 131.8, 131.3 (*ipso*-C_6_H_3_); 134.5, 131.2, 129.6, 129.4, 129.3, 126.3, 124.3 (C_6_H_4_ + C_6_H_3_); 123.2 (C^1^); 90.8, 88.1 (Cp); 76.3 (CH_2_); 48.6 (C_β_); 45.7 (NMe); 20.0 (O=C*Me*); 17.1, 16.5 (C_6_H_3_*Me*_2_). Crystals of **2** suitable for X-ray analysis were obtained by slow evaporation of the solvent from a methanol solution.

**Chart 1:**
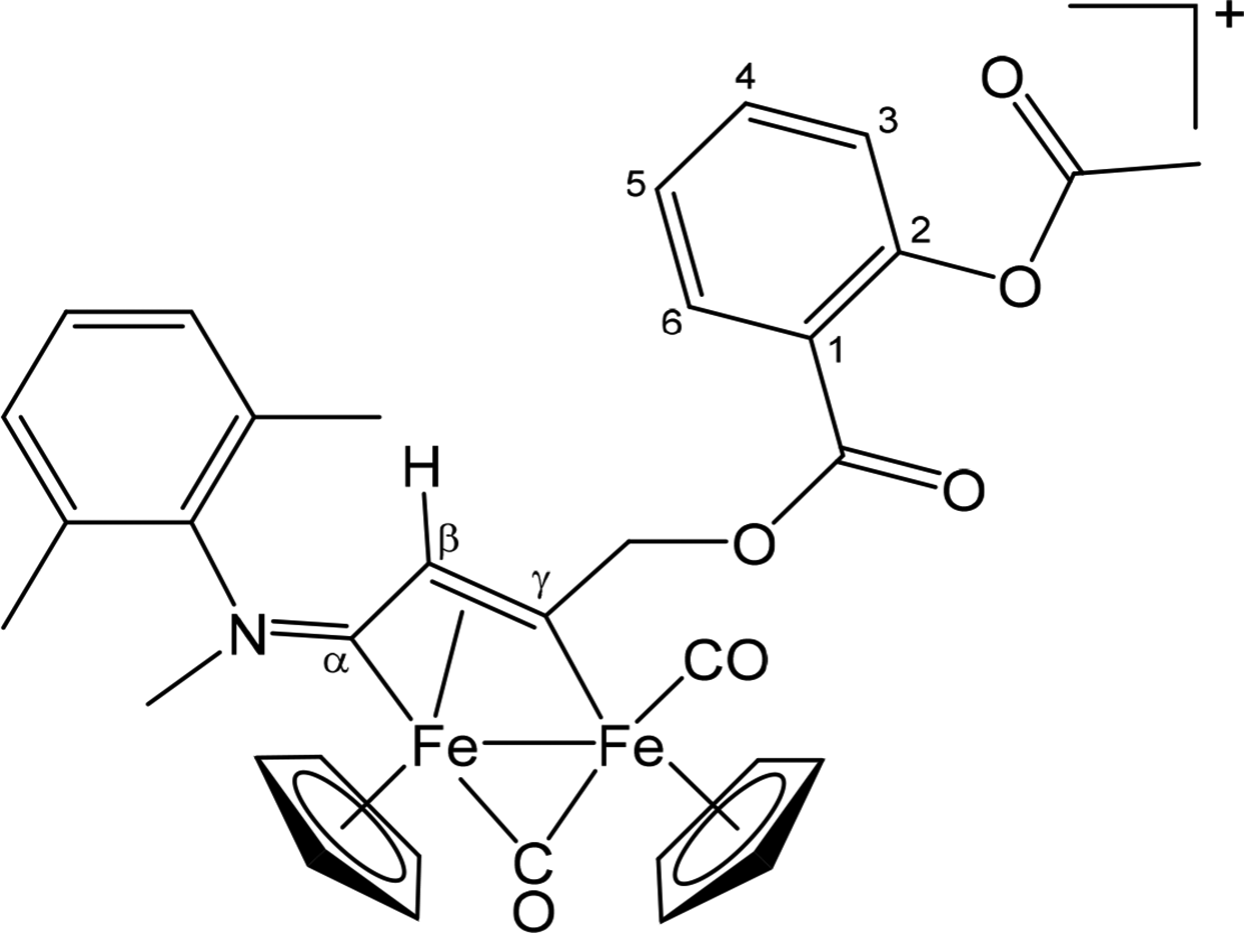
Structure of [**2**]^+^

##### [Fe_2_Cp_2_(CO)(μ-CO){μ-η^1^:η^3^-C_γ_(3-C_6_H_4_OC(=O)C_6_H_4_OC(=O)Me)C_β_HC_α_NMe_2_}]CF_3_SO_3_ ([**3**]CF_3_SO_3_) (Chart 2)

This compound was obtained from [**1a**]CF_3_SO_3_ and **ALK**^**A2**^: brown solid, yield 48%. Anal. Calcd for C_33_H_28_F_3_Fe_2_NO_9_S: C, 50.60; H, 3.60; N, 1.79; S, 4.09. Found: C, 50.45; H, 3.65; N, 1.83; S, 4.03. IR (CH_2_Cl_2_): ν̃/cm^–1^ 1993vs (CO), 1809s (μ-CO), 1767m (Me*C=O*), 1743s (C_6_H_4_*C=O*), 1692m (C_β_C_α_N), 1607w (C–C_arom_), 1577w (C–C_arom_). ^1^H NMR (CDCl_3_): δ/ppm 8.28 (dd, ^3^*J*_H3–H4_ = 7.8 Hz, ^4^*J*_H3–H5_ = 1.4 Hz, 1 H, H^3^); 7.71 (t, ^3^*J* = 7.6 Hz, 1 H, H^5^); 7.58 (t, ^3^J = 8.1 Hz, 1 H, H^4^); 7.51, 7.49–7.41, 7.25–7.18 (m, 5 H, H^6^ + H^8^ + H^10^ + H^11^ + H^12^); 5.25, 5.02 (s, 10 H, Cp); 4.84 (s, 1 H, C_β_H); 3.96, 3.43 (s, 6 H, NMe_2_); 2.30 (s, 3 H, O=CMe). ^13^C{^1^H} NMR (CDCl_3_): δ/ppm 255.7 (μ-CO); 224.5 (C_α_); 209.4 (CO); 201.3 (C_γ_); 169.9 (O=*C*Me); 163.5 (C_6_H_4_*C*=O); 157.0 (C^7^); 150.9, 150.3 (C^2^ + C^9^); 134.9, 132.2, 130.1, 126.4, 124.5, 123.8, 121.0, 120.1 (C_6_H_4_); 122.5 (C^1^); 91.3, 87.8 (Cp); 52.9 (C_β_); 51.7, 44.6 (NMe_2_); 21.2 (O=C*Me*).

**Chart 2:**
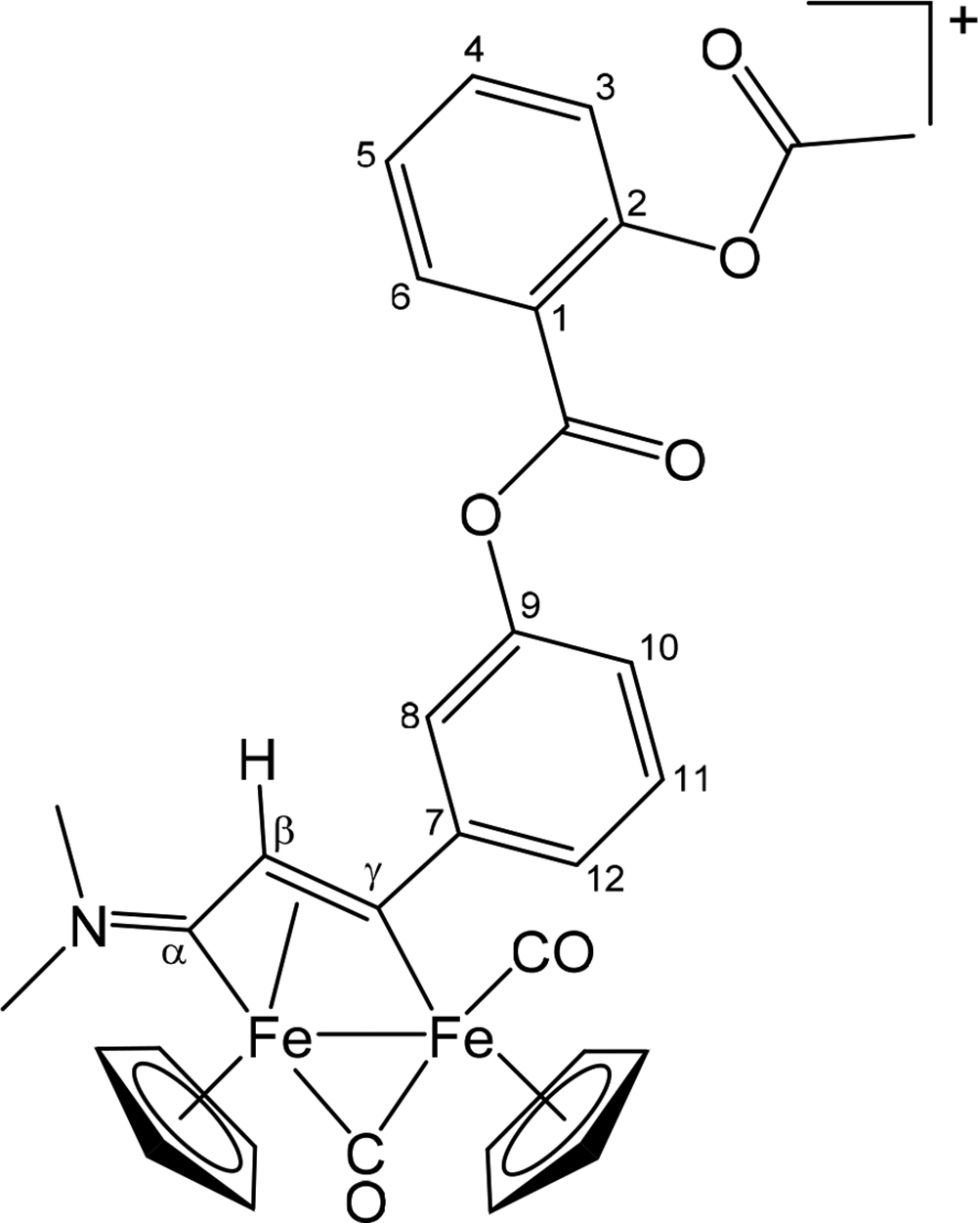
Structure of [**3**]^+^

##### [Fe_2_Cp_2_(CO)(μ-CO){μ-η^1^:η^3^-C_γ_(3-C_6_H_4_OC(=O)C_6_H_4_OC(=O)Me)C_β_HC_α_NMe(Xyl)}]CF_3_SO_3_ ([**4**]CF_3_SO_3_) (Chart 3)

This compound was obtained from [**1b**]CF_3_SO_3_ and **ALK**^**A2**^: brown solid, yield 86%. Anal. Calcd. for C_40_H_34_F_3_Fe_2_NO_9_S: C, 55.00; H, 3.924; N, 1.60; S, 3.67. Found: C, 54.87; H, 3.95; N, 1.55; S, 3.52. IR (CH_2_Cl_2_): ν̃/cm^–1^ 2004vs (CO), 1819s (μ-CO), 1768m (Me*C=O*), 1744s (C_6_H_4_*C=O*), 1631m (C_β_C_α_N), 1607w (C–C_arom_), 1578w (C–C_arom_). ^1^H NMR (acetone-*d*_6_): δ/ppm 8.23, 7.79, 7.65–7.00 (m, 11 H, C_6_H_4_ + C_6_H_3_); 5.70, 5.47 (s, 10 H, Cp); 4.44 (br s, 4 H, NMe + C_β_H); 2.37 (s, 3 H, O=CMe); 2.25, 1.89 (s, 6 H, C_6_H_3_*Me*_2_). ^13^C{^1^H} NMR (acetone-*d*_6_): δ/ppm 253.1 (μ-CO); 232.1 (C_α_); 210.1 (CO); 169.0 (O=*C*Me); 163.1 (C_6_H_4_*C*=O); 157.2 (C^7^); 151.1, 150.4 (C^2^ + C^9^); 145.4, 132.0, 131.3 (*ipso*-C_6_H_3_); 134.9, 131.8, 129.7, 129.6, 129.3, 129.3, 126.2, 124.1, 124.1, 120.8, 120.1 (C_6_H_4_ + C_6_H_3_); 122.9 (C^1^); 92.5, 88.2 (Cp); 53.8 (C_β_); 45.5 (NMe); 20.2 (O=C*Me*); 17.3, 16.6 (C_6_H_3_*Me*_2_). C_γ_ overlapped with solvent signal.

**Chart 3:**
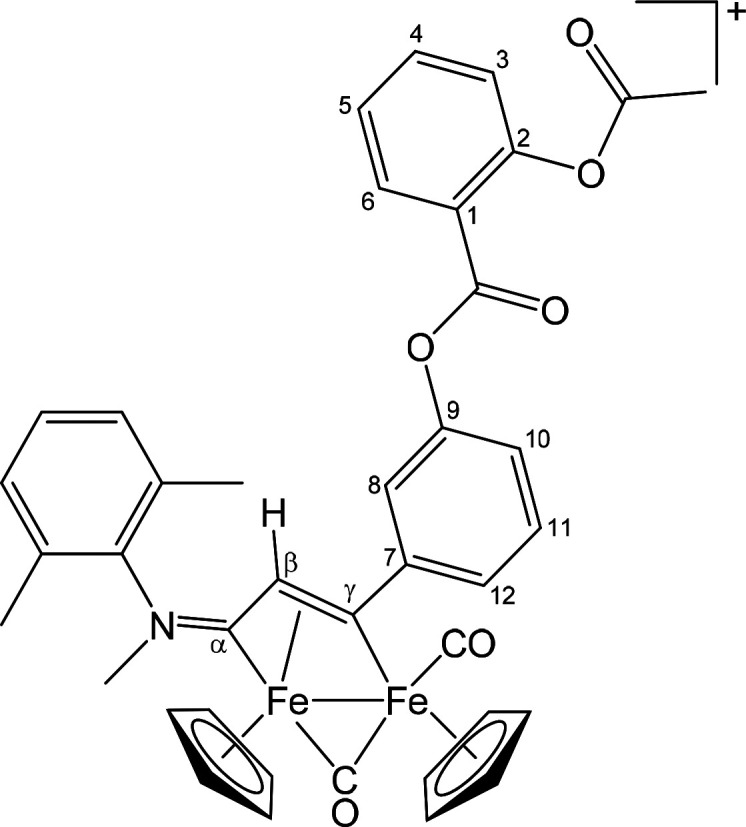
Structure of [**4**]^+^

##### [Fe_2_Cp_2_(CO)(μ-CO){μ-η^1^:η^3^-C_γ_(3-C_6_H_4_NHC(=O)C_6_H_4_OC(=O)Me)C_β_HC_α_NMe_2_}]CF_3_SO_3_ ([**5**]CF_3_SO_3_) (Chart 4)

This compound was obtained from [**1a**]CF_3_SO_3_ and **ALK**^**A3**^: brown solid, yield 96%. Anal. Calcd for C_33_H_29_F_3_Fe_2_N_2_O_8_S: C, 50.66; H, 3.74; N, 3.58; S, 4.10. Found: C, 50.74; H, 3.62; N, 3.68; S, 4.00. IR (CH_2_Cl_2_): ν̃/cm^–1^ 1991vs (CO), 1807s (μ-CO), 1770m (Me*C=O*), 1678s (C_β_C_α_N + N*C = O*), 1604m (C–C_arom_), 1582m (C–C_arom_), 1537s. ^1^H NMR (acetone-d_6_): δ/ppm 9.75 (s, 1 H, NH); 8.70, 7.90–7.20 (m, 8 H, C_6_H_4_); 5.45, 5.31 (s, 10 H, Cp); 4.74 (s, 1 H, C_β_H); 4.07, 3.48 (s, 6 H, NMe_2_); 2.23 (s, 3 H, O=CMe). ^13^C NMR (acetone-*d*_6_): δ/ppm 255.8 (μ-CO); 224.9 (C_α_); 210.0 (CO); 203.6 (C_γ_); 168.7 (O=Me); 164.8 (NC=O); 156.7 (C^7^); 148.7 (C^2^); 139.1 (C^9^); 129.6 (C^1^); 131.8, 129.1, 128.8, 125.9, 123.4, 122.4, 119.2, 118.2 (C_6_H_4_); 91.7, 87.9 (Cp); 53.0 (C_β_); 50.9, 44.2 (NMe_2_); 20.2 (O=C*Me*).

**Chart 4:**
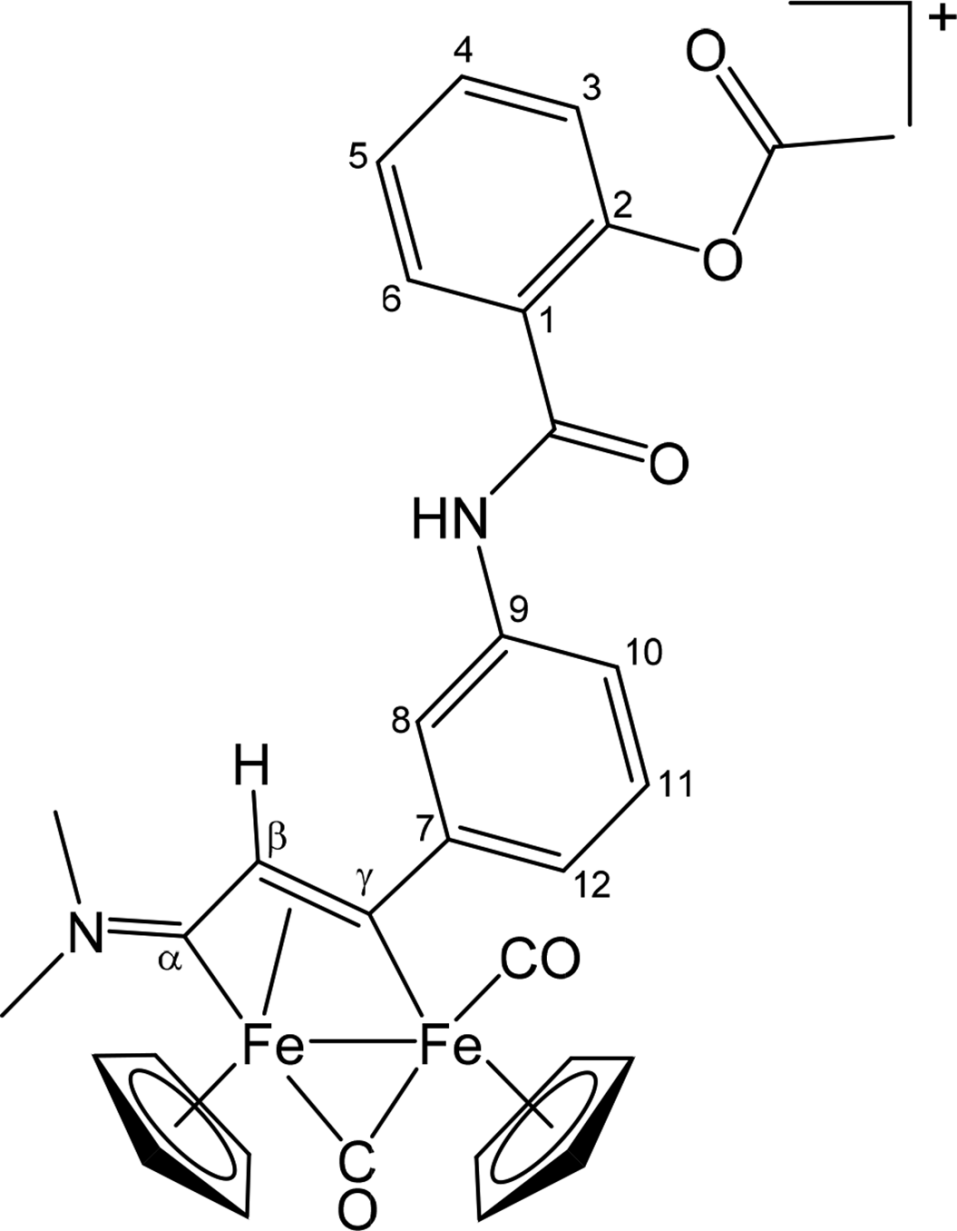
Structure of [**5**]^+^

##### [Fe_2_Cp_2_(CO)(μ-CO){μ-η^1^:η^3^-C_γ_(3-C_6_H_4_NHC(=O)C_6_H_4_OC(=O)Me)C_β_HC_α_NMe(Xyl)}]CF_3_SO_3_ ([**6**]CF_3_SO_3_) (Chart 5)

This compound was obtained from [**1b**]CF_3_SO_3_ and **ALK**^**A3**^: brown solid, yield 95%. Anal. Calcd. for C_40_H_35_F_3_Fe_2_N_2_O_8_S: C, 55.07; H, 4.04; N, 3.21; S, 3.68. Found: C, 55.25; H, 4.03; N, 3.25; S, 3.62. IR (CH_2_Cl_2_): ν̃/cm^–1^ 2003vs (CO), 1816s (μ-CO), 1771w (Me*C=O*), 1678m (N*C=O*), 1628m (C_β_C_α_N), 1605m (C–C_arom_), 1583m (C–C_arom_), 1535s. ^1^H NMR (acetone-*d*_6_): δ/ppm 9.74 (s, 1 H, NH); 8.40 (s, 1 H, H^8^); 7.68, 7.44, 7.15 (m, 3 H, H^10^ + H^11^ + H^12^); 7.82, 7.59, 7.40, 7.24 (m, 4 H, H^3^ + H^4^ + H^5^ + H^6^); 7.26, 7.19, 7.10 (m, 3 H, C_6_H_3_); 5.69, 5.43, *5.38*, *5.09* (s, 10 H, Cp); 4.43, *2.62* (s, 3 H, NMe); 4.36 (s, 1 H, C_β_H); 2.36, 1.89 (s, 6 H, C_6_H_3_*Me*_2_); 2.21 (s, 3 H, O=CMe). *E*/*Z* ratio 9. ^13^C NMR (acetone-*d*_6_): δ/ppm 253.4 (μ-CO); 232.2 (C_α_); 210.3 (CO); 207.8 (C_γ_); 168.6 (O=*C*Me); 164.7 (NC=O); 156.4 (C^7^); 148.7 (C^2^); 145.5, 132.0, 131.3 (*ipso*-C_6_H_3_); 139.0 (C^9^); 129.6, 129.3, 129.1 (C_6_H_3_); 129.1 (C^1^); 131.8, 129.3, 125.9, 123.4 (C^3^ + C^4^ + C^5^ + C^6^); 128.8, 122.0, 118.2 (C^10^ + C^11^ + C^12^); 118.2 (C^8^); 92.5, *92.4*, 88.2, *88.0* (Cp); 53.8 (C_β_); 45.5 (NMe); 20.2 (O=C*Me*); 17.3, 16.6 (C_6_H_3_*Me*_2_).

**Chart 5:**
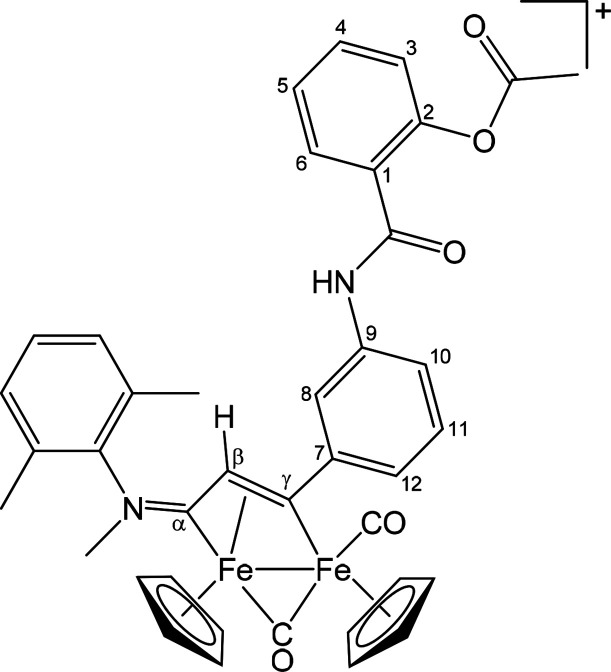
Structure of [**6**]^+^

##### [Fe_2_Cp_2_(CO)(μ-CO){μ-η^1^:η^3^-C_γ_(4-C_6_H_4_NHC(=O)C_6_H_4_OC(=O)Me)C_β_HC_α_NMe_2_}]CF_3_SO_3_ ([**7**]CF_3_SO_3_) (Chart 6)

This compound was obtained from [**1a**]CF_3_SO_3_ and **ALK**^**A4**^: brown solid, yield 81%. Anal. Calcd for C_33_H_29_F_3_Fe_2_N_2_O_8_S: C, 50.66; H, 3.74; N, 3.58; S, 4.10. Found: C, 50.52; H, 3.78; N, 3.51; S, 4.16. IR (CH_2_Cl_2_): ν̃/cm^–1^ 1991vs (CO), 1807s (μ-CO), 1773w (Me*C=O*), 1681s (C_β_C_α_N + N*C = O*), 1604m (C–C_arom_), 1589m (C–C_arom_), 1518s. ^1^H NMR (acetone-*d*_6_): δ/ppm 9.65 (s, 1 H, NH); 7.98, 7.95–7.70, 7.60, 7.42, 7.26 (m, 8 H, C_6_H_4_); 5.41, 5.30 (s, 10 H, Cp); 4.66 (s, 1 H, C_β_H); 4.05, 3.47 (s, 6 H, NMe_2_); 2.29 (s, 3 H, O=CMe). ^13^C{^1^H} NMR (acetone-*d*_6_): δ/ppm 256.0 (μ-CO); 225.3 (C_α_); 210.1 (CO); 203.3 (C_γ_); 168.6 (O=*C*Me); 152.3 (NC=O); 148.7, 138.4, 131.7, 129.7, 129.1, 128.1, 125.9, 123.5, 119.4, 119.4 (C_6_H_4_); 91.6, 87.7 (Cp); 52.7 (C_β_); 50.8, 44.1 (NMe_2_); 20.1 (O=C*Me*).

**Chart 6:**
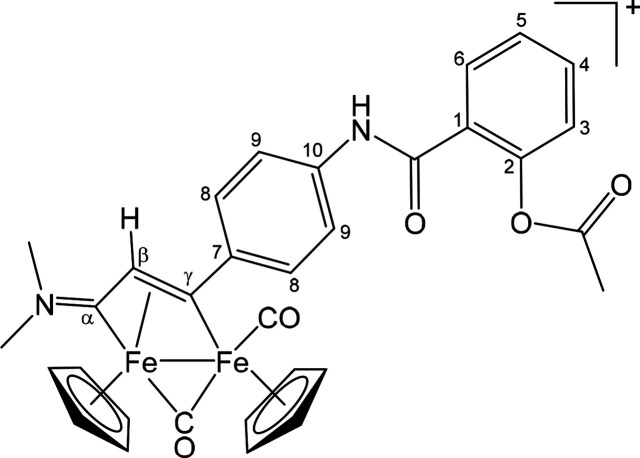
Structure of [**7**]^+^

##### [Fe_2_Cp_2_(CO)(μ-CO){μ-η^1^:η^3^-C_γ_(3-C_6_H_4_OC(=O)(CH_2_)_3_C_6_H_4_N(CH_2_CH_2_Cl)_2_)C_β_HC_α_NMe_2_}]CF_3_SO_3_ ([**8**]CF_3_SO_3_) (Chart 7)

This compound was obtained from [**1a**]CF_3_SO_3_ and **ALK**^**C1**^: brown solid, yield 87%. Anal. Calcd for C_38_H_39_Cl_2_F_3_Fe_2_N_2_O_7_S: C, 50.30; H, 4.33; N, 3.09; S, 3.53. Found: C, 50.21; H, 4.26; N, 3.15; S, 3.58. IR (CH_2_Cl_2_): ν̃/cm^–1^ 1992vs (CO), 1809s (μ-CO), 1758m (O*C=O*), 1690w (C_β_C_α_N), 1615w (C–C_arom_), 1578w (C–C_arom_), 1519s. ^1^H NMR (acetone-*d*_6_): δ/ppm 7.71 (d, ^3^*J*_H10–H9_ = 7.3 Hz, 1 H, H^10^); 7.63 (br s, 1 H, H^6^); 7.59 (t, ^3^*J* = 7.9 Hz, 1 H, H^9^); 7.19 (d, ^3^*J*_H8–H9_ = 7.9 Hz, 1 H, H^8^); 7.15 (d, ^3^*J*_H3–H2_ = 8.6 Hz, 2 H, H^3^); 6.78 (d, ^3^*J*_H2–H3_ = 8.7 Hz, 2 H, H^2^); 5.42, 5.29 (s, 10 H, Cp); 4.72 (s, 1 H, C_β_H); 4.06, 3.48 (s, 6 H, NMe_2_); 3.81 (m, 4 H, NCH_2_); 3.75 (m, 4 H, CH_2_Cl); 2.67 (m, 4 H, H^11^ + H^13^); 2.08 (m, 2 H, H^12^). ^13^C{^1^H} NMR (CDCl_3_): δ/ppm 256.3 (μ-CO); 224.2 (C_α_); 209.5 (CO); 202.0 (C_γ_); 172.3 (C=O); 156.7 (C^5^); 150.5 (C^7^); 144.5 (C^1^); 130.2 (C^4^); 129.8 (C^3^ + C^10^); 123.9, 120.8 (C^6^ + C^9^); 120.0 (C^8^); 112.3 (C^2^); 91.0, 87.8 (Cp); 53.6 (NCH_2_); 53.2 (C_β_); 52.0, 44.7 (NMe_2_); 40.6 (CH_2_Cl); 34.0 (C^13^); 33.9 (C^11^); 26.7 (C^12^).

**Chart 7:**
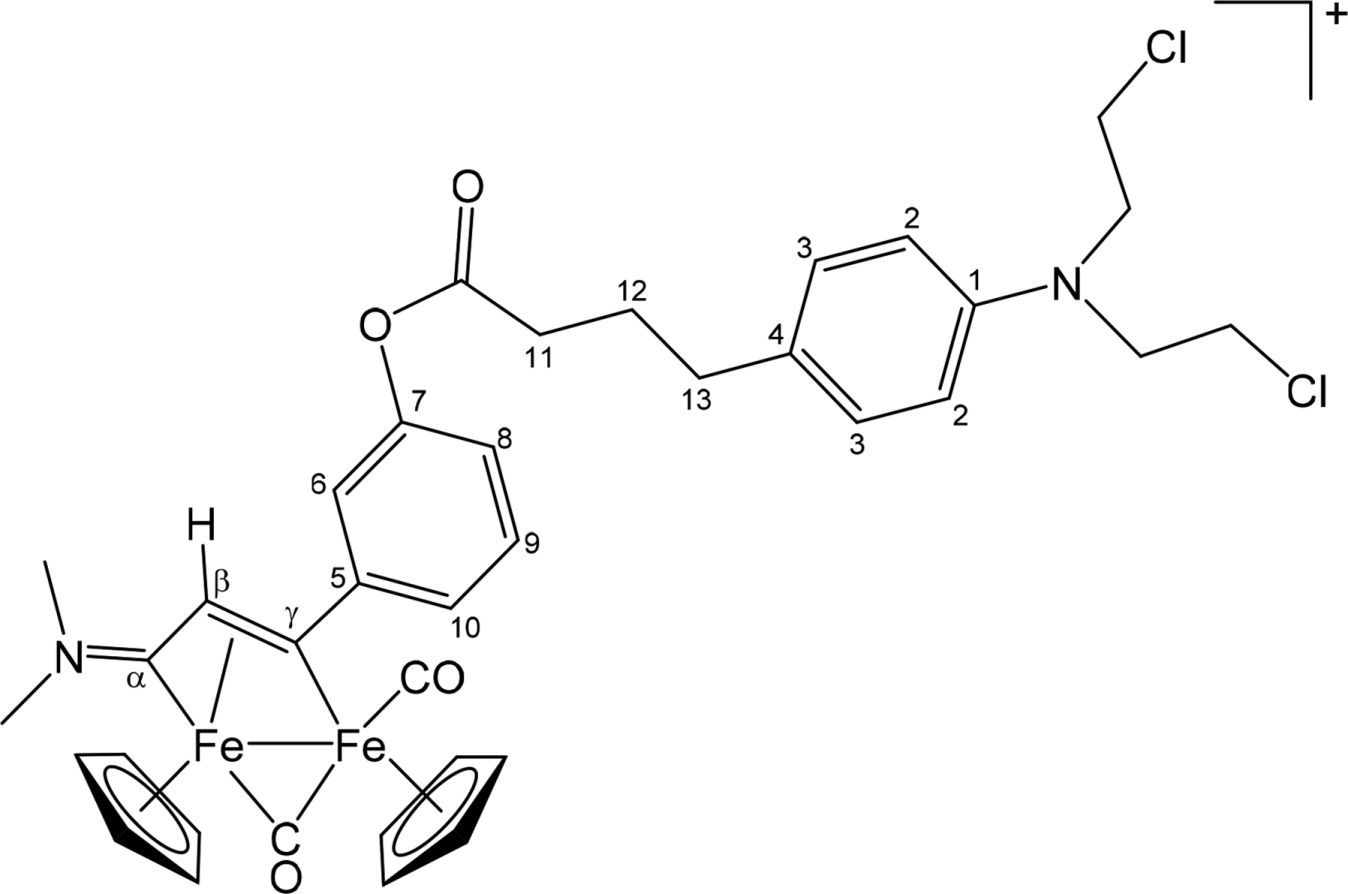
Structure of [**8**]^+^

##### [Fe_2_Cp_2_(CO)(μ-CO){μ-η^1^:η^3^-C_γ_(3-C_6_H_4_NHC(=O)(CH_2_)_3_C_6_H_4_N(CH_2_CH_2_Cl)_2_)C_β_HC_α_NMe_2_}]CF_3_SO_3_ ([**9**]CF_3_SO_3_) (Chart 8)

This compound was obtained from [**1a**]CF_3_SO_3_ and **ALK**^**C2**^: brown solid, yield 81%. Anal. Calcd for C_38_H_40_Cl_2_F_3_Fe_2_N_3_O_6_S: C, 50.35; H, 4.45; N, 4.64; S, 3.54. Found: C, 50.24; H, 4.53; N, 4.57; S, 3.61. IR (CH_2_Cl_2_): ν̃/cm^–1^ 1991vs (CO), 1808s (μ-CO), 1683br-s (C_β_C_α_N + N*C=O*), 1615m (C–C_arom_), 1602m, 1584m (C–C_arom_), 1519vs. ^1^H NMR (acetone-*d*_6_): δ/ppm 9.52 (s, 1 H, NH); 8.53 (s, 1 H, H^6^); 7.52 (d, ^3^*J*_H8–H9_ = 7.9 Hz, 1 H, H^8^); 7.45 (t, ^3^*J* = 7.7 Hz, 1 H, H^9^); 7.38 (d, ^3^*J*_H10–H9_ = 7.1 Hz, 1 H, H^10^); 7.12 (d, ^3^*J*_H3–H2_ = 8.2 Hz, 2 H, H^3^); 6.74 (d, ^3^*J*_H2–H3_ = 8.2 Hz, 2 H, H^2^); 5.41, 5.26 (s, 10 H, Cp); 4.68 (s, 1 H, C_β_H); 4.04, 3.45 (s, 6 H, NMe_2_); 3.78 (m, 4 H, NCH_2_); 3.74 (m, 4 H, CH_2_Cl); 2.61 (t, ^3^*J*_H13–H12_ = 7.4 Hz, 2 H, H^13^); 2.48 (t, ^3^*J*_H11–H12_ = 7.2 Hz, 2 H, H^11^); 1.99 (m, 2 H, H^12^). ^13^C{^1^H} NMR (acetone-*d*_6_): δ/ppm 255.9 (μ-CO); 225.1 (C_α_); 210.0 (CO); 204.1 (C_γ_); 171.8 (C=O); 156.6 (C^5^); 144.7 (C^1^); 139.5 (C^7^); 130.6 (C^4^); 129.5 (C^3^); 128.6 (C^9^); 121.6 (C^10^); 118.5 (C^6^); 117.4 (C^8^); 112.3 (C^2^); 91.7, 87.8 (Cp); 53.1 (NCH_2_); 52.9 (C_β_); 50.8, 44.2 (NMe_2_); 40.8 (CH_2_Cl); 36.4 (C^11^); 34.0 (C^13^); 27.4 (C^12^).

**Chart 8:**
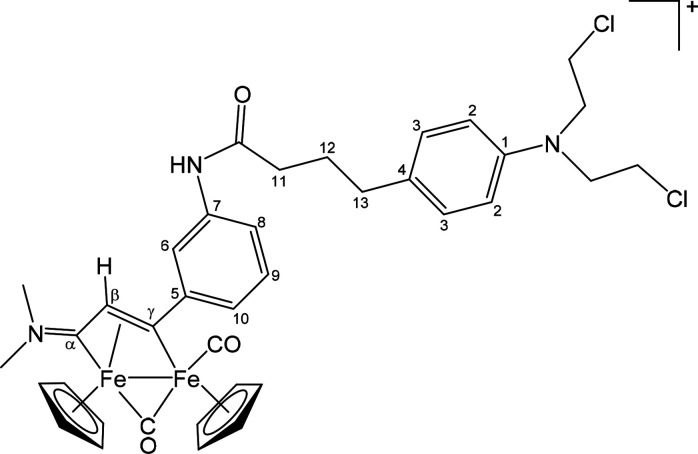
Structure of [**9**]^+^

##### [Fe_2_Cp_2_(CO)(μ-CO){μ-η^1^:η^3^-C_γ_(4-C_6_H_4_NHC(=O)(CH_2_)_3_C_6_H_4_N(CH_2_CH_2_Cl)_2_)C_β_HC_α_NMe_2_}]CF_3_SO_3_ ([**10**]CF_3_SO_3_) (Chart 9)

This compound was obtained from [**1a**]CF_3_SO_3_ and **ALK**^**C3**^: brown solid, yield 64%. Anal. Calcd for C_38_H_40_Cl_2_F_3_Fe_2_N_3_O_6_S: C, 50.35; H, 4.45; N, 4.64; S, 3.54. Found: C, 50.23; H, 4.51; N, 4.69; S, 3.62. IR (CH_2_Cl_2_): ν̃/cm^–1^ = 1992vs (CO), 1808s (μ-CO), 1691br-s (C_β_C_α_N + N*C=O*), 1614m (C–C_arom_), 1606m, 1586m (C–C_arom_), 1518vs. ^1^H NMR (acetone-*d*_6_): δ/ppm 9.38 (s, 1 H, NH); 7.87 (d, ^3^*J*_H7–H6_ = 8.5 Hz, 2 H, H^7^); 7.79 (d, ^3^*J*_H6–H7_ = 8.6 Hz, 2 H, H^6^); 7.14 (d, ^3^*J*_H3–H2_ = 8.6 Hz, 2 H, H^3^); 6.78 (d, ^3^*J*_H2–H3_ = 8.6 Hz, 2 H, H^2^); 5.40, 5.29 (s, 10 H, Cp); 4.63 (s, 1 H, C_β_H); 4.05, 3.47 (s, 6 H, NMe_2_); 3.80 (m, 4 H, NCH_2_); 3.77 (m, 4 H, CH_2_Cl); 2.64 (t, ^3^*J*_H11–H10_ = 7.3 Hz, 2 H, H^11^); 2.46 (t, ^3^*J*_H9–H10_ = 7.5 Hz, 2 H, H^9^); 2.02 (m, 2 H, H^10^). ^13^C{^1^H} NMR (acetone-*d*_6_): δ/ppm 256.2 (μ-CO); 225.4 (C_α_); 210.1 (CO); 203.7 (C_γ_); 171.4 (C=O); 151.6 (C^5^); 144.8 (C^1^); 138.8 (C^8^); 130.7 (C^4^); 129.5 (C^3^); 128.0 (C^6^); 118.7 (C^7^); 112.3 (C^2^); 91.6, 87.7 (Cp); 53.1 (NCH_2_); 52.6 (C_β_); 50.9, 44.2 (NMe_2_); 40.8 (CH_2_Cl); 36.3 (C^9^); 34.0 (C^11^); 27.5 (C^10^).

**Chart 9:**
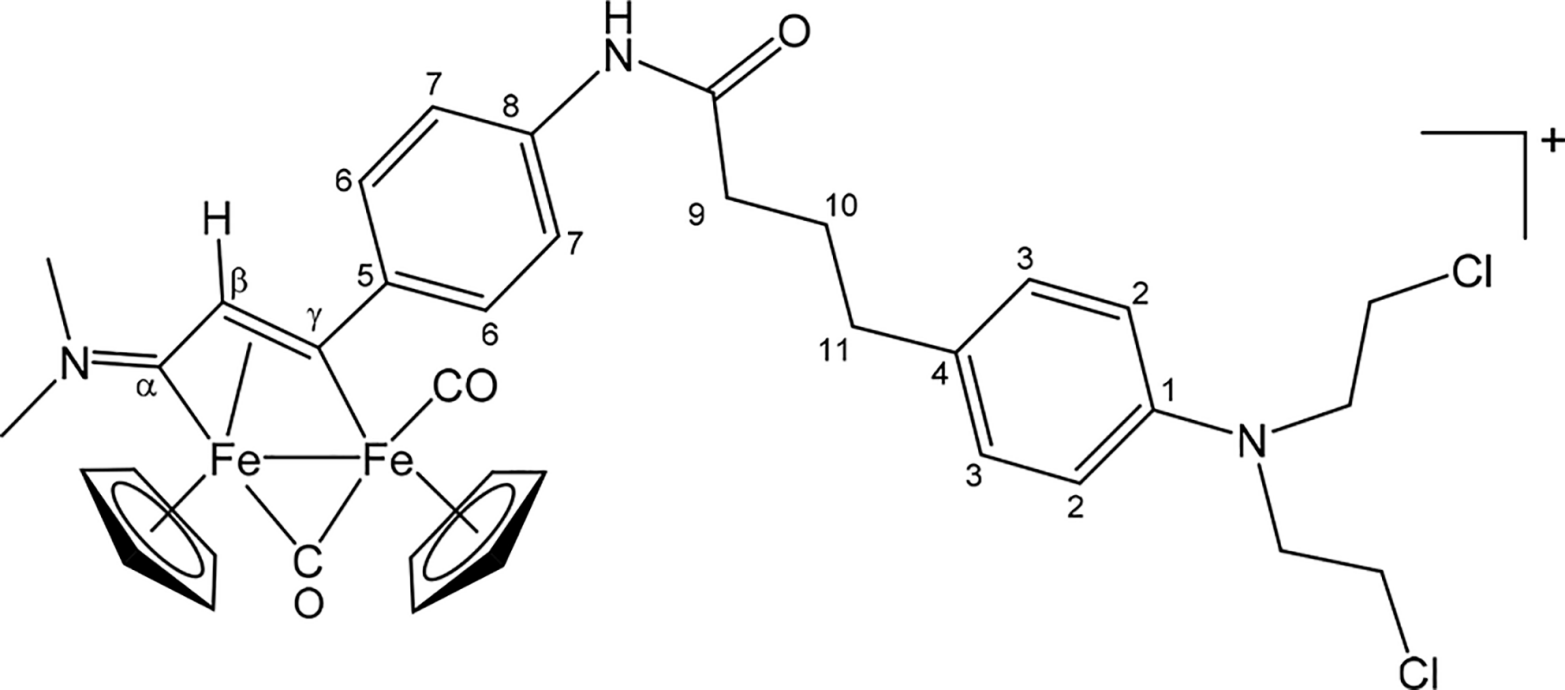
Structure of [**10**]^+^

### Cell Culture and Cytotoxicity Studies

Human ovarian carcinoma (A2780 and A2780cisR) cell lines were obtained from the European Collection of Cell Cultures. The Human Embryonic Kidney 293T (HEK 293T) cell line was obtained from the ATCC (Sigma, Buchs, Switzerland). Penicillin–streptomycin, RPMI 1640 GlutaMAX (where RPMI = Roswell Park Memorial Institute), and DMEM GlutaMAX media (where DMEM = Dulbecco’s modified Eagle medium) were obtained from Life Technologies, and fetal bovine serum (FBS) was obtained from Sigma. The cells were cultured in RPMI 1640 GlutaMAX (A2780 and A2780cisR) and DMEM GlutaMAX (HEK 293T) media containing 10% heat-inactivated FBS and 1% penicillin–streptomycin at 37 °C and CO_2_ (5%). The A2780cisR cell line was routinely treated with cisplatin (2 μM) in the media to maintain cisplatin resistance. The cytotoxicity was determined using the 3-(4,5-dimethyl-2-thiazolyl)-2,5-diphenyl-2*H*-tetrazolium bromide (MTT) assay.^42^ Cells were seeded in flat-bottomed 96-well plates as a suspension in a prepared medium (100 μL aliquots and approximately 4300 cells/well) and preincubated for 24 h. Stock solutions of compounds were prepared in DMSO and were diluted in the medium. The solutions were sequentially diluted to give a final DMSO concentration of 0.5% and a final compound concentration range of 0–200 μM. Cisplatin was tested as a positive (0–100 μM) control. The compounds were placed in the preincubated 96-well plates in 100 μL aliquots, and the plates were incubated for a further 72 h. MTT (20 μL, 5 mg/mL in Dulbecco’s phosphate buffered saline) was placed in the cells, and the plates were incubated for a further 4 h. The culture medium was aspirated, and the purple formazan crystals, formed by the mitochondrial dehydrogenase activity of vital cells, were dissolved in DMSO (100 μL/well). The absorbance of the resulting solutions, directly proportional to the number of surviving cells, was quantified at 590 nm using a SpectroMax M5e multimode microplate reader (using SoftMax Pro software, version 6.2.2). The percentage of surviving cells was calculated from the absorbance of wells corresponding to the untreated control cells. The reported IC_50_ values are based on the means from two independent experiments, each comprising four tests per concentration level.

### ROS Determination

The intracellular production of reactive oxygen species (ROS) upon treatment of the complexes [**4**]CF_3_SO_3_ and [**9**]CF_3_SO_3_ was measured by using the DCFH-DA (2′,7′-dichlorodihydrofluorescein diacetate, Sigma-Aldrich) assay, based on the cellular uptake of the nonfluorescent diacetate following deacetylation by esterases (2′,7′-dichlorodihydrofluorescein, DCFH) and oxidation to the fluorescent dichlorofluorescein (2′,7′-dichlorofluorescein, DCF).^43^ A2780 cells were seeded at a concentration of 4 × 10^4^ cells per well in 90 μL of complete growth medium into 96-well plates. After overnight incubation, the cells were treated following the manufacturer’s protocol. A 100 μL portion of a solution containing the fluorogenic probe was added to the culture medium and, after 1 h of incubation with 5% CO_2_ at 37 °C, cells were exposed with a final concentration of 10 μM of the tested compound; H_2_O_2_ 100 μM was used as a positive control. Stock solutions of compounds were prepared as described above; cells incubated with equal amounts of DMSO in supplemented RPMI were used as a control. The fluorescence was measured up to 24 h with an excitation wavelength of 485 nm and with a 535 nm emission filter by Multilabel Counter (PerkinElmer, Waltham, MA, USA). The measurements were performed in triplicate, and results were reported as mean ± SD. Statistical differences were examined using one-way analysis of variance (ANOVA), and a Tukey test was used for *post hoc* analysis. A *p* value <0.05 was considered statistically significant.

### COX-2 Inhibition Assays

The enzymatic activity of COX-2 (0.25 UN) was fluorimetrically assayed at 576 nm/586 nm at 25 °C by measuring the conversion rate of arachidonic acid (ARA) into resorufin by COX-2 as a function of time (COX-2 assay kit from Cayman Chemical Company, Ann Arbor, MI, USA). The assay mixture contained 25 μM ADHP, 5 μM hemin, and 37.5 μM ARA in 0.1 M Tris-HCl buffer (pH 8). The inhibitory efficacy of the tested compounds was determined by recording the residual activity of COX-2 in the presence of variable concentrations of the analyzed compound (11–705 μM). The IC_50_ value was obtained using GraphPad Prism 7 software. All of the inhibitor assays were performed at least in triplicate.

### Interaction with Biomolecules

Ethidium bromide (EB, ≥98.0%), bovine serum albumin (BSA, lyophilized powder, crystallized, ≥98.0%), and natural double-stranded DNA from calf thymus (DNA, lyophilized powder, Na^+^ salt in the form of fibers) were purchased from Merck, while anhydrous guanosine 5′-monophosphate disodium salt (Na_2_[GMP], >98.0%) was purchased from TCI Chemicals. Prior to use, DNA was sonicated to ca. 500 base pair length (MSE-Sonyprep sonicator, 7 cycles of 10 s sonication + 20 s pause at an amplitude of 14 μm, solution kept in ice bath—the final length was checked by agarose gel electrophoresis tests on the sample using a 100 bp DNA ladder) to produce stock solutions in water (ca. 2 mM). Stock solutions of EB and BSA were prepared by weighing directly in the buffer (NaCl 0.1 M, NaCac 0.01M, pH 7.0—NaCac is sodium cacodylate). A temperature-controlled (±0.1 °C) Shimadzu UV2450 spectrophotometer and PerkinElmer LS55 spectrofluorometer were the instruments used. Molar concentrations of solutions of EB (*C*_EB_), BSA (*C*_BSA_), and DNA (*C*_DNA_) were determined by absorbance measurements (ε_EB_^480 nm^ = 5700 M^–1^ cm^–1^; ε_BSA_^278 nm^ = 44000 M^–1^ cm^–1^; ε_DNA_^260 nm^ = 13200 M^–1^ cm^–1^ for a concentration expressed in base pairs). Solutions of the metal complex were prepared by weighing an appropriate amount of the solid and dissolving it in DMSO (ca. 5 mM). Ultrapure water (Sartorius) was the reaction medium. In EB/DNA exchange experiments, EB was added to DNA until the fluorescence emission at λ_ex_ = 520 nm/λ_em_ = 595 nm (diagnostic wavelengths for the DNA-intercalated EB only) started to reach the plateau (*C*_EB_ = 5.60 × 10^–5^ M, *C*_DNA_ = 1.15 × 10^–4^ M). The concentrated stock solution of the metal complex was then directly added in small amounts to the EB/DNA mixture with a gastight syringe connected to a Mitutoyo micrometric screw (1 total turn of the screw = 8.2 μL). The total maximum amount of DMSO was checked (<3%) as well as the metal complex absorbance at the set wavelengths (*A* < 0.05 to ensure negligible inner filter effects). Note that a blank test was carried out by adding DMSO to the EB/DNA mixture, in order to quantify fluorescence changes due only to dilution/solvent effects. For BSA fluorescence titrations, the working solution of the metal complex (4.42 × 10^–5^ M for [**4**]CF_3_SO_3_, 4.59 × 10^–5^ M for [**9**]CF_3_SO_3_, both in the buffer) was added to a 3.34 × 10^–7^ M BSA solution in the buffer (λ_ex_ = 280 nm, λ_em_ = 345 nm, dilution from stock such that the DMSO content is negligible). Each experiment was performed at least in duplicate; the reported values are mean values over the replicates (error <5%).

In order to analyze the interaction with a model nucleotide, a freshly prepared solution of [**9**]CF_3_SO_3_ (2 mg) in D_2_O/CD_3_OD (5/3 v/v) was treated with 1 equiv of anhydrous Na_2_[GMP]. The resulting mixture was maintained at 37 °C for 24 h, and then the final solution was filtered in order to remove some solid and analyzed by mass spectrometry. HPLC-MS(+): *m*/*z* found 1087.2451 [**9**^**GMP**^ + Na]^+^, calcd for C_47_H_53_Fe_2_N_8_NaO_12_P 1087.2117; *m*/*z* found 555.0666 [**9**^**GMP**^ + 2Na]^2+^, calcd for C_47_H_53_Fe_2_N_8_Na_2_O_12_P 555.1008. The isotopic patterns fit well the calculated patterns.

## Supporting Information Available

Synthesis and characterization of alkynes, X-ray crystallography, solubility and stability in aqueous media, determination of partition coefficients, BSA binding studies, and NMR spectra of products (PDF)

## Accession Codes

CCDC 2076710–2076712 contain the supplementary crystallographic data for this paper. These data can be obtained free of charge via www.ccdc.cam.ac.uk/data_request/cif, or by emailing data_request@ccdc.cam.ac.uk, or by contacting The Cambridge Crystallographic Data Centre, 12 Union Road, Cambridge CB2 1EZ, UK; fax: +44 1223 336033.
