# Supplementary material for: A Strategy to Conjugate Bioactive Fragments to Cytotoxic Diiron Bis(cyclopentadienyl) Complexes
Source: Organometallics. 2021 Jul 2;40(15):2516–28. doi: 10.1021/acs.organomet.1c00270 (PMC8397425; doi:10.1021/acs.organomet.1c00270)
Supplement: Supplementary file 1 — om1c00270_si_001.pdf [file om1c00270_si_001.pdf]

Supporting Information

A Strategy to Conjugate Bioactive Fragments to Cytotoxic Diiron Bis-Cyclopentadienyl Complexes

Silvia Schoch, Mouna Hadiji, Sarah A. P. Pereira, M. Lúcia M. F. S. Saraiva, Simona Braccini, Federica Chiellini, Tarita Biver, Stefano Zacchini, Guido Pampaloni, Paul J. Dyson, Fabio Marchetti

[a] University of Pisa, Dipartimento di Chimica e Chimica Industriale, 56124 Pisa, Italy.

[b] Institut des Sciences et Ingénierie Chimiques, Ecole Polytechnique Fédérale de Lausanne (EPFL), Switzerland.

[c] LAQV, REQUIMTE, Laboratório de Química Aplicada, Faculdade de Farmácia da Universidade do Porto, Portugal.

[d] University of Pisa, Dipartimento di Farmacia, 56126 Pisa, Italy.

[e] University of Bologna, Dipartimento di Chimica Industriale "Toso Montanari", 40136 Bologna, Italy.

<u>**Table of contents**</u> *Pages*

**Synthesis and characterization of bio-derivatized alkynes**	S2-S9
**X-ray crystallography**	S10
**Solubility and stability in aqueous media**	S11-S13
**Determination of partition coefficients (Log P_{ow})**	S14
**Figures S1-S2**: BSA binding	S15-S16
**Figures S3-S42**: ^{1}H and ^{13}C NMR spectra of products	S17-S41
**References**	S42

Synthesis and characterization of bio-derivatized alkynes

Prop-2-yn-1-yl 2-acetoxybenzoate, ALK^{A1} (Chart S1) [1]

Chart S1. Structure of **ALK^{A1}**

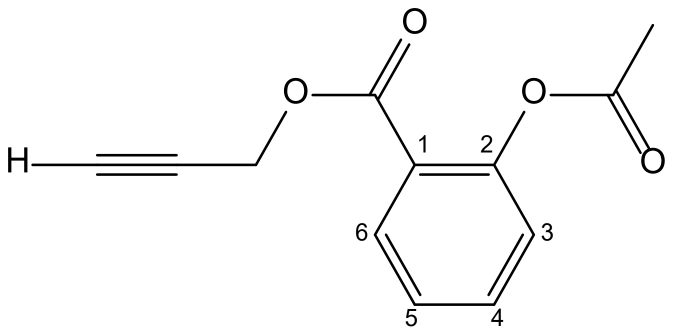

The title compound was prepared by a modified literature procedure. A solution of acetylsalicylic acid (93 mg, 0.516 mmol) in CH_2Cl_2 (8 mL) was treated with propargyl alcohol (37 µL, 0.636 mmol), DMAP (4 mg, 0.033 mmol) and EDCI (155 mg, 0.809 mmol), in the given order. The resulting mixture was stirred overnight at ambient temperature. The complete conversion of acetylsalicylic acid was checked by silica TLC (petroleum ether/diethyl ether 3:1 *v/v*). The reaction solution was concentrated up to ca. 3 mL, and then charged on a silica column. Elution with petroleum ether/diethyl ether mixture (3:1 *v/v*) allowed to separate the fraction corresponding to **ALK^{A1}**. Removal of the solvent under reduced pressure afforded the product as a white solid. Yield 74 mg (66%). Anal. calcd. for $C_{12}H_{10}O_4$: C, 66.05; H, 4.62. Found: C, 65.88; H, 4.75. IR (solid): $\tilde{\upsilon}$/cm^{-1} = 3240m-s, 2962w, 2935w, 2129w (C≡C), 1751s (Me*C=O*), 1721vs (C_6H_4*C=O*), 1607m, 1579w, 1488m, 1446w-m, 1428w-m, 1374m-s, 1299w-m, 1257vs, 1223vs, 1200vs, 1165m-s, 1139m-s, 1080vs, 1039m, 1014m-s, 994m, 968m, 961m, 923m-s, 882m, 852w-m, 813m-s, 798m-s, 755vs, 747s, 701vs, 669m, 653w-m. ^{1}H NMR ($CDCl_3$): δ/ppm = 8.07 (dd, $^3J_{H3-H4}$ = 7.9 Hz, $^4J_{H3-H5}$ = 1.6 Hz, 1 H, H^3); 7.59 (td, ^3J = 7.9 Hz, $^4J_{H5-H3}$ = 1.7 Hz, 1 H, H^5); 7.33 (td, ^3J = 7.8 Hz, $^4J_{H4-H6}$ = 0.9 Hz, 1 H, H^4); 7.13 (dd, $^3J_{H6-H5}$ = 8.1 Hz, $^4J_{H6-H4}$ = 0.8 Hz, 1 H, H^6); 4.89 (d, ^4J = 2.5 Hz, 2 H, CH_2); 2.56 (t, ^4J = 2.5 Hz, 1 H, C≡CH); 2.38 (s, 3 H, O=CMe).

3-Ethynylphenyl 2-acetoxybenzoate, ALK^{A2} (Chart S2)

Chart S2. Structure of **ALK^{A2}**

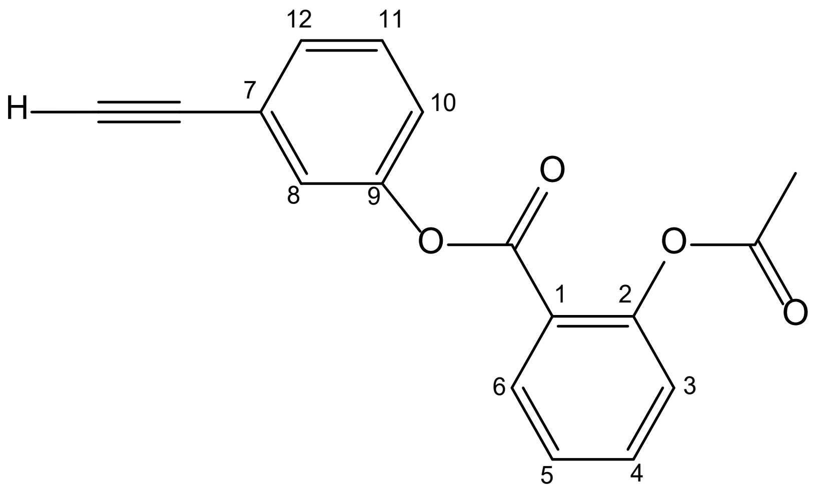

A solution of acetylsalicylic acid (722 mg, 4.01 mmol) in CH_2Cl_2 (12 mL) was treated with 3-hydroxyphenylacetylene (0.3 mL, 2.75 mmol), DMAP (29 mg, 0.237 mmol) and EDCI (920 mg, 4.80 mmol), in the given order. The resulting mixture was stirred overnight at ambient temperature. The complete conversion of 3-hydroxyphenylacetylene was checked by silica TLC (petroleum ether/diethyl ether 5:1 *v/v*). The reaction solution was concentrated up to ca. 5 mL, and then charged on a silica column. Elution with petroleum ether/diethyl ether mixture (5:1 *v/v*) allowed to separate the fraction corresponding to **ALK^{A2}**. Yield 396 mg (51%). Anal. calcd. for $C_{17}H_{12}O_4$: C, 72.85; H, 4.32. Found: C, 73.03; H, 4.53. IR (solid): ũ/cm^{-1} = 3298m, 3069w, 2962w, 2924w, 2111w (C≡C), 1771m-s (Me*C=O*), 1740vs (C_6H_4*C=O*), 1604m, 1574m, 1486sh, 1478m, 1451w, 1422w, 1363w-m, 1300w, 1276sh-w, 1260sh-m, 1249m-s, 1238sh, 1225vs, 1188vs, 1162m-s, 1139vs, 1097br-m, 1081sh, 1054vs, 1044sh-s, 1009sh-s, 999vs, 966m, 920m-s, 910m-s, 900m, 879m, 853m, 824sh, 800sh, 794s, 777s, 759s, 732w-m, 719w-m, 696m-s, 686m-s, 674m, 654s. ^{1}H NMR ($CDCl_3$): δ/ppm = 8.24 (d, $^3J_{H3-H4}$ = 7.8 Hz, 1 H, H^3); 7.67 (t, ^3J = 7.7 Hz, 1 H, H^5); 7.48-7.37 (m, 3 H, H^4 + H^{10} + H^{11}); 7.35 (br s, 1 H, H^8); 7.21 (m, 2 H, H^6 + H^{12}); 3.15 (s, 1 H, C≡CH); 2.34 (s, 3 H, O=CMe). ^{13}C NMR ($CDCl_3$): δ/ppm = 169.7 (O=*C*Me); 162.7 (C_6H_4*C*=O); 151.3, 150.3 (C^2 + C^9); 134.8 (C^5); 132.2 (C^3); 129.9 (C^{10}); 129.5 (C^4); 126.2 (C^{11}); 125.4 (C^8); 124.1 (C^6); 123.7 (C^7); 122.6 (C^{12}); 122.3 (C^1); 82.5 (*C*≡CH); 78.2 (C≡*C*H); 21.0

(O=C*Me*). Colorless X-ray quality crystals of **ALK**^{A2} were obtained by slow evaporation of the solvent from a petroleum ether/diethyl ether solution.

2-[(3-Ethynylphenyl)carbamoyl]phenyl acetate, ALK^{A3} (Chart S3)

Chart S3. Structure of **ALK^{A3}**

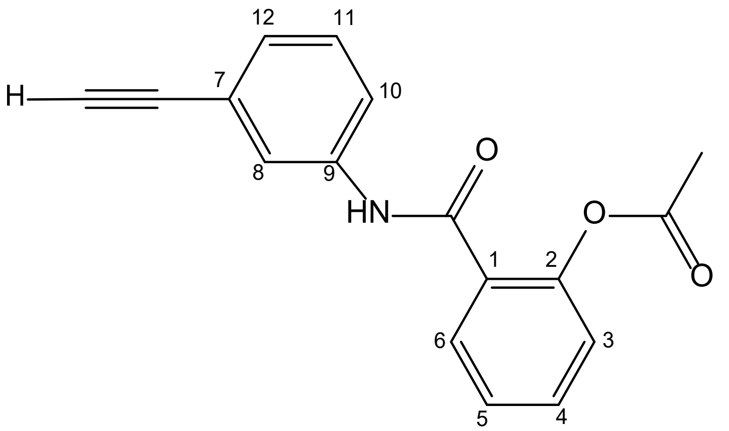

A solution of acetylsalicylic acid (1.176 g, 6.53 mmol) in CH_2Cl_2 (25 mL) was treated with oxalyl chloride (1.70 mL, 19.8 mmol) and then with one drop of dimethylformamide. The resulting solution was stirred for 1.5 hours at ambient temperature. The volatiles were removed under reduced pressure at 60 °C, thus affording acetylsalicylic acid chloride as a light-yellow oil.[2] This oil was dissolved in CH_2Cl_2 (20 mL), then 3-ethynylaniline (0.6 mL, 5.33 mmol) and triethylamine (2.5 mL, 17.9 mmol) were added to the solution. The resulting mixture was stirred overnight. The complete conversion of 3-ethynylaniline was checked by silica TLC (petroleum ether/diethyl ether 1:1 *v/v*). The reaction solution was concentrated up to ca. 5 mL, and then charged on a silica column. Elution with petroleum ether/diethyl ether mixture (1:1 *v/v*) allowed to separate impurities. The fraction corresponding to the title compound was collected by using petroleum ether/diethyl ether mixture (2:3 *v/v*) as eluent; slow evaporation of the solvent afforded colorless X-ray quality crystals of **ALK^{A3}**. Yield 1.175 g (79%). Anal. calcd. for $C_{17}H_{13}NO_3$: C, 73.11; H, 4.69; N, 5.02. Found: C, 72.95; H, 4.61; N, 5.15. IR (solid): $\tilde{\upsilon}$/cm^{-1} = 3302m, 3247m (NH), 3053w-br, 2087w (C≡C), 1750m-s, 1740m-s (Me*C=O*), 1673vs (N*C=O*), 1602m, 1584w, 1532s, 1482s, 1445w, 1427w, 1407m, 1374m, 1305s, 1230vs, 1207vs, 1171w, 1160m, 1130vw, 1085m, 1047w, 1018m, 969vw, 955w-m, 934w, 911w-m, 895w, 872s,

827w-m, 781s, 759s, 740m, 674vs-br, 657s. ^{1}H NMR ($CDCl_3$): δ/ppm = 8.05 (s, 1 H, NH); 7.86 (dd, $^3J_{H3-H4}$ = 7.8 Hz, $^4J_{H3-H5}$ = 1.2 Hz, 1 H, H^3); 7.74 (br s, 1 H, H^8); 7.66 (d, $^3J_{H10-H11}$ = 7.9 Hz, 1 H, H^{10}); 7.55 (ddd, $^3J_{H5-H4}$ = 8.2 Hz, $^3J_{H5-H6}$ = 7.5 Hz, $^4J_{H5-H3}$ = 1.7 Hz, 1 H, H^5); 7.39 (td, ^3J = 7.7 Hz, $^4J_{H4-H6}$ = 1.1 Hz, 1 H, H^4); 7.34 (t, ^3J = 7.9 Hz, 1 H, H^{11}); 7.30 (dt, $^3J_{H12-H11}$ = 7.7 Hz, ^4J = 1.4 Hz, 1 H, H^{12}); 7.19 (dd, $^3J_{H6-H5}$ = 8.2 Hz, $^4J_{H6-H4}$ = 0.8 Hz, 1 H, H^6); 3.08 (s, 1 H, C≡CH); 2.31 (s, 3 H, O=CMe). ^{13}C NMR ($CDCl_3$): δ/ppm = 169.3 (O=*C*Me); 163.9 (NC=O); 147.8 (C^2); 137.9 (C^9); 132.3, 129.8, 129.2, 128.4, 126.5, 123.3, 120.5 (C^3 + C^4 + C^5 + C^6 + C^8 + C^{10} + C^{11} + C^{12}); 128.5 (C^1); 123.0 (C^7); 83.1 (*C*≡CH); 77.7 (C≡*C*H); 21.0 (O=C*Me*).

2-[(4-Ethynylphenyl)carbamoyl]phenyl acetate, ALK^{A4} (Chart S4)

Chart S4. Structure of **ALK^{A4}**

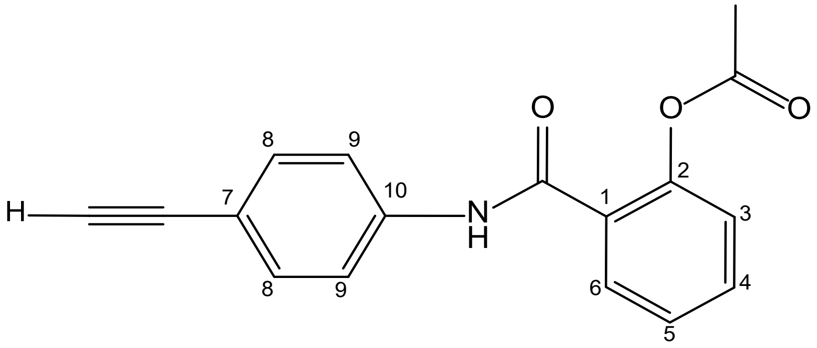

The title compound was prepared by a procedure analogous to that described for the synthesis of **ALK^{A3}**, from acetylsalicylic acid (123 mg, 0.683 mmol) and 4-ethynylaniline (69 mg, 0.589 mmol). Yellow solid, yield 156 mg (95%). Anal. calcd. for $C_{17}H_{13}NO_3$: C, 73.11; H, 4.69; N, 5.02. Found: C, 73.25; H, 4.57; N, 5.21. IR (solid): ṽ/cm^{-1} = 3290w-m, 3262w-m (NH), 3178w, 3107w, 3056w, 2963w-m, 2927 w-br, 2855w, 2105w (C≡C), 1759m, 1748m (Me*C=O*), 1657m-s (N*C=O*), 1593m-s, 1506m-s, 1444w-m, 1402m, 1371w-m, 1324m-s, 1296w, 1259s, 1240w, 1217w, 1190sh, 1173m-s, 1082vs, 1014vs-br, 953m, 912m, 897m, 865m, 832s, 796vs-br, 781sh, 754m, 730w, 707w, 675w-m, 664m. ^{1}H NMR ($CDCl_3$): δ/ppm = 8.29 (s, 1 H, NH); 7.87 (dd, $^3J_{H3-H4}$ = 7.7 Hz, $^4J_{H3-H5}$ = 1.5 Hz, 1 H, H^3); 7.63 (d, $^3J_{H9-H8}$ = 8.6 Hz, 2 H, H^9); 7.54 (ddd, $^3J_{H5-H6}$ = 8.1 Hz, $^3J_{H5-H4}$ = 7.5 Hz, $^4J_{H5-H3}$ = 1.7 Hz, 1 H, H^5); 7.50 (d, $^3J_{H8-H9}$ = 8.7 Hz, 2 H, H^8); 7.38 (td, ^3J = 7.6 Hz, $^4J_{H4-H6}$ = 1.1 Hz, 1 H, H^4); 7.17 (dd, $^3J_{H6-H5}$ = 8.1 Hz, $^4J_{H6-H4}$ = 1.0 Hz, 1 H, H^6); 3.08 (s, 1 H, C≡CH); 2.34 (s, 3 H, O=CMe). ^{13}C NMR ($CDCl_3$):

δ/ppm = 169.3 (O=*C*Me); 163.6 (NC=O); 147.8 (C^2); 138.2 (C^{10}); 133.1 (C^9); 132.4, 130.0, 126.7, 123.4 (C^3 + C^4 + C^5 + C^6); 128.6 (C^1); 119.4 (C^8); 118.2 (C^7); 83.3 (*C*≡CH); 21.1 (O=C*Me*). C≡*C*H overlapped with solvent signal.

3-Ethynylphenyl 4-[4-(bis(2-chloroethyl)amino)phenyl]butanoate, ALK^{C1} (Chart S5)

Chart S5. Structure of **ALK^{C1}**

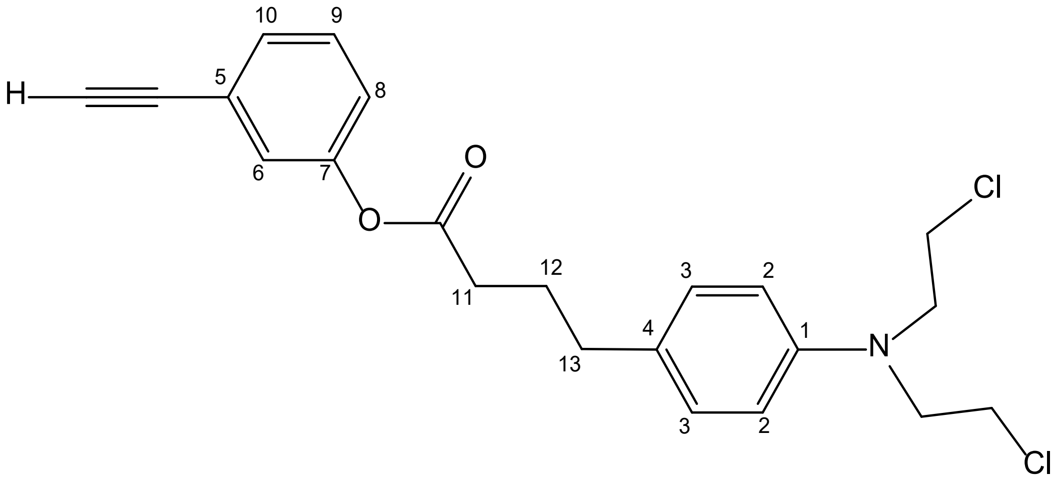

A solution of 4-[4-(bis(2-chloroethyl)amino)phenyl]butanoic acid (chlorambucil; 195 mg, 0.641 mmol) in CH_2Cl_2 (10 mL) was added of 3-hydroxyphenylacetylene (48 µL, 0.440 mmol), DMAP (4 mg, 0.0327 mmol) and EDCI (143 mg, 0.746 mmol), in the given order. The resulting mixture was stirred overnight, thus the complete conversion of 3-hydroxyphenylacetylene was checked by NMR spectroscopy. The reaction solution was concentrated up to ca. 2 mL, and then charged on a silica column. Elution with petroleum ether/diethyl ether mixture (5:2 *v/v*) allowed to collect a fraction corresponding to **ALK^{C1}**, which was isolated as a colorless oil upon removal of the solvent under vacuum. Yield 167 mg (94%). Anal. calcd. for $C_{22}H_{23}Cl_2NO_2$: C, 65.35; H, 5.73; N, 3.46. Found: C, 65.18; H, 5.78; N, 3.31. IR (solid): $\tilde{\nu}$/cm^{-1} = 3290br-w-m, 3096w, 3073w, 3034w, 3011w, 2953w-m, 2929w-m, 2857w-m, 2109w (C≡C), 1757s (C=O), 1615m-s, 1578m, 1517vs, 1479m, 1446w-m, 1425w-m, 1387w, 1353m-s, 1307w, 1276w-m, 1250sh, 1234m-s, 1206m-s, 1178s, 1136vs, 1114vs, 1035w, 1000w, 985vw, 947w-m, 915w-m, 894m, 825sh, 796s, 739m-s, 684s, 657s. ^{1}H NMR ($CDCl_3$): δ/ppm = 7.38 (m, 1 H, H^{10}); 7.34 (t, ^3J = 7.7 Hz, 1 H, H^9); 7.22 (br, m, 1 H, H^6); 7.13 (d, $^3J_{H3\text{-}H2}$ = 8.6 Hz, 2 H, H^3); 7.09 (dt, $^3J_{H8\text{-}H9}$ = 7.6 Hz, ^4J = 1.8, 1 H, H^8); 6.67 (d, $^3J_{H2\text{-}H3}$ = 8.7 Hz, 2 H, H^2);

3.74 (m, 4 H, NCH_2); 3.65 (m, 4 H, CH_2Cl); 3.12 (s, 1 H, C≡CH); 2.68 (t, $^3J_{H13-H12}$ = 7.5 Hz, 2 H, H^{13}); 2.59 (t, $^3J_{H11-H12}$ = 7.4 Hz, 2 H, H^{11}); 2.06 (p, ^3J = 7.4 Hz, 2 H, H^{12}). ^{13}C NMR ($CDCl_3$): δ/ppm = 171.8 (C=O); 150.5 (C^7); 144.6 (C^1); 130.3 (C^4); 129.8 (C^3); 129.6 (C^{10}); 129.4 (C^9); 125.3 (C^6); 123.5 (C^5); 122.5 (C^8); 112.3 (C^2); 82.7 (*C*≡CH); 78.3 (C≡*C*H); 53.6 (NCH_2); 40.7 (CH_2Cl); 34.0 (C^{13}); 33.6 (C^{11}); 26.7 (C^{12}).

4-[4-(Bis(2-chloroethyl)amino)phenyl]-N-(3-ethynylphenyl)butanamide, ALK^{C2} (Chart S6)

Chart S6. Structure of **ALK^{C2}**

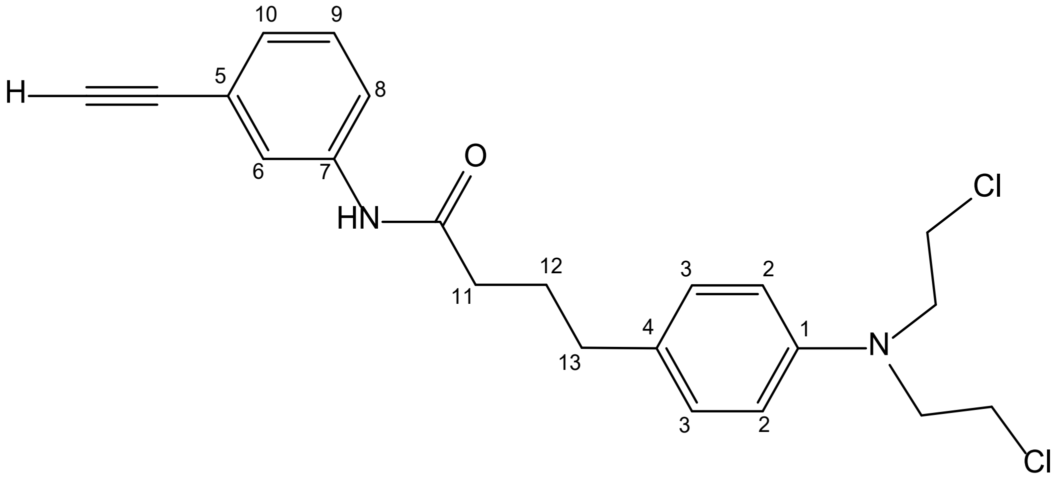

A solution of 4-[4-(bis(2-chloroethyl)amino)phenyl]butanoic acid (chlorambucil; 246 mg, 0.809 mmol) in CH_2Cl_2 (10 mL) was treated with oxalyl chloride (0.21 mL, 2.45 mmol) and then with dimethylformamide (1 µL, 0.0129 mmol). The resulting solution was stirred for 3 hours at ambient temperature, then the volatiles were removed under reduced pressure at 60 °C; thus 4-[4-(bis(2-chloroethyl)amino)phenyl]butanoic acid chloride was obtained as a light-green oil.[3] This oil was dissolved in CH_2Cl_2 (10 mL), and the solution was treated with 3-ethynylaniline (74 µL, 0.657 mmol) and triethylamine (0.280 mL, 2.01 mmol). The resulting mixture was stirred overnight, and the complete conversion of 3-ethynylaniline was checked by silica TLC (petroleum ether/diethyl ether 2:1 *v/v*). The reaction solution was concentrated up to ca. 4 mL, and then charged on a silica column. Elution with petroleum ether/diethyl ether mixtures at increasing polarity (from 2:1 to 1:2 *v/v*) allowed to separate impurities. The fraction corresponding to the title compound was collected using petroleum ether/diethyl ether mixture (3:4 *v/v*) as eluent, then **ALK^{C2}** was isolated as a crystalline white solid upon evaporation of the solvent under vacuum. Yield 167 mg (63%). Anal. calcd. for

$C_{22}H_{24}Cl_2N_2O$: C, 65.51; H, 6.00; N, 6.95. Found: C, 65.38; H, 6.10; N, 7.04. IR (solid): ῦ/cm^{-1} = 3292br-w-m, 3275w-m (NH), 2963m, 2923sh, 2867sh, 2109w (C≡C), 1652m (C=O), 1614w, 1603w, 1518m-s, 1485w-m, 1473w, 1454w, 1405w-m, 1364w, 1352w, 1259s, 1215w, 1194vw, 1176w-m, 1144w, 1084br-s, 1014br-vs, 967sh-w, 920vw, 878m, 793br-vs, 744m-s, 677m-s. ^{1}H NMR ($CDCl_3$): δ/ppm = 7.63 (s, 1 H, H^6); 7.55 (d, $^3J_{H8-H9}$ = 7.6 Hz, 1 H, H^8); 7.35 (s, br, 1 H, NH); 7.28 (m, 1 H, H^9); 7.24 (m, 1 H, H^{10}); 7.09 (d, $^3J_{H3-H2}$ = 8.6 Hz, 2 H, H^3); 6.64 (d, $^3J_{H2-H3}$ = 8.7 Hz, 2 H, H^2); 3.71 (m, 4 H, NCH_2); 3.63 (m, 4 H, CH_2Cl); 3.08 (s, 1 H, C≡CH); 2.63 (t, $^3J_{H13-H12}$ = 7.4 Hz, 2 H, H^{13}); 2.36 (t, $^3J_{H11-H12}$ = 7.4 Hz, 2 H, H^{11}); 2.03 (p, ^3J = 7.4 Hz, 2 H, H^{12}). ^{13}C NMR ($CDCl_3$): δ/ppm = 171.2 (C=O); 144.5 (C^1); 138.0 (C^7); 130.4 (C^4); 129.8 (C^3); 129.0 (C^9); 127.9 (C^{10}); 123.1 (C^6); 122.8 (C^5); 120.3 (C^8); 112.3 (C^2); 83.2 (*C*≡CH); 77.5 (C≡*C*H); 53.6 (NCH_2); 40.6 (CH_2Cl); 36.8 (C^{11}); 34.0 (C^{13}); 27.0 (C^{12}).

4-[4-(Bis(2-chloroethyl)amino)phenyl]-N-(4-ethynylphenyl)butanamide, ALK^{C3} (Chart S7)

Chart S7. Structure of **ALK^{C3}**

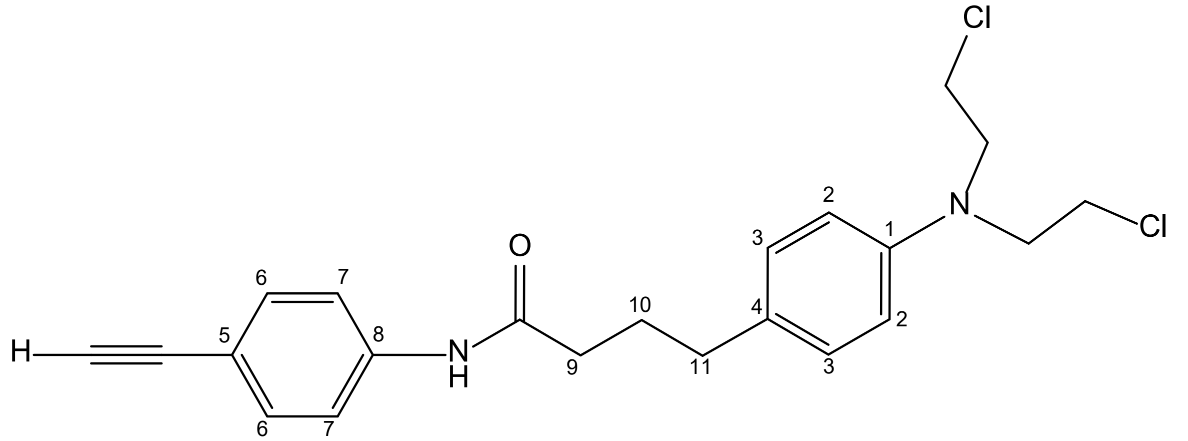

The title compound was prepared by a procedure analogous to that described for the synthesis of **ALK^{C2}**, from chlorambucil (198 mg, 0.651 mmol) and 4-ethynylaniline (60 mg, 0.512 mmol). White solid, yield 137 mg (66%). Anal. calcd. for $C_{22}H_{24}Cl_2N_2O$: C, 65.51; H, 6.00; N, 6.95. Found: C, 65.31; H, 6.11; N, 7.06. IR (solid): ῦ/cm^{-1} = 3273m, 3249sh (NH), 3177w, 3106w, 3038w, 2959w-m, 2923m, 2855w-m, 2103w (C≡C), 1737br, 1680w, 1658vs (C=O), 1615m, 1595vs, 1539sh, 1517vs, 1508sh, 1469m, 1453m-s, 1445sh, 1402m-s, 1387m, 1359s, 1343m-s, 1305m, 1274m, 1245s, 1213m-s, 1177vs, 1144vs, 1110w-m, 1027br-m, 966m, 832vs, 810vs, 767m, 734br-vs, 674vs. ^{1}H NMR (acetone-d_6): δ/ppm = 9.30 (s, 1 H, NH); 7.70 (d, $^3J_{H7-H6}$ = 8.5

Hz, 2 H, H^7); 7.42 (d, $^3J_{H6\text{-}H7}$ = 8.4 Hz, 2 H, H^6); 7.10 (d, $^3J_{H3\text{-}H2}$ = 8.4 Hz, 2 H, H^3); 6.73 (d, $^3J_{H2\text{-}H3}$ = 8.4 Hz, 2 H, H^2); 3.77 (m, 4 H, NCH_2); 3.74 (m, 4 H, CH_2Cl); 3.56 (s, 1 H, C≡CH); 2.59 (t, $^3J_{H11\text{-}H10}$ = 7.5 Hz, 2 H, H^{11}); 2.40 (t, $^3J_{H9\text{-}H10}$ = 7.4 Hz, 2 H, H^9); 1.96 (p, ^3J = 7.4 Hz, 2 H, H^{10}). ^{13}C NMR (acetone-d_6): δ/ppm = 171.2 (C=O); 144.7 (C^1); 140.2 (C^8); 132.5 (C^6); 130.5 (C^4); 129.5 (C^3); 118.9 (C^7); 116.6 (C^5); 112.2 (C^2); 83.4 (*C*≡CH); 77.3 (C≡*C*H); 53.1 (NCH_2); 40.8 (CH_2Cl); 36.2 (C^9); 33.9 (C^{11}); 27.2 (C^{10}).

X-ray crystallography

Crystal data and collection details for **ALK^{A2}**, **ALK^{A3}** and [**2**]CF_3SO_3 are reported in Table S1. Data were recorded on a Bruker APEX II diffractometer equipped with a PHOTON100 detector using Mo–Kα radiation. Data were corrected for Lorentz polarization and absorption effects (empirical absorption correction SADABS).[4] The structures were solved by direct methods and refined by full-matrix least-squares based on all data using F^2.[5] Hydrogen atoms were fixed at calculated positions and refined by a riding model, except the N-bonded hydrogen of **ALK^{A3}** which was located in the Fourier map and refined isotropically. All non-hydrogen atoms were refined with anisotropic displacement parameters. The unit cell off [**2**]CF_3SO_3 contains an additional total potential solvent accessible void of 199 Å^3, which is likely to be occupied by a highly disordered solvent molecule. These voids have been treated using the SQUEEZE routine of PLATON.[6]

Table S1. Crystal data and measurement details for **ALK^{A2}**, **ALK^{A3}** and [**2**]CF_3SO_3.

	**ALK^{A2}**	**ALK^{A3}**	[**2**]CF_3SO_3
Formula	$C_{17}H_{12}O_4$	$C_{17}H_{13}NO_3$	$C_{35}H_{32}F_3Fe_2NO_9S$
FW	280.27	279.28	811.37
T, K	100(2)	100(2)	100(2)
λ, Å	0.71073	0.71073	0.71073
Crystal system	Triclinic	Orthorhombic	Triclinic
Space group	$P\bar{1}$	*Pbca*	$P\bar{1}$
a, Å	8.3748(2)	14.6788(10)	10.5063(6)
b, Å	8.3839(2)	8.9225(7)	10.9552(6)
c, Å	10.2531(3)	20.9573(15)	16.9954(10)
α,°	103.9120(10)	90	75.508(2)
β,°	97.1590(10)	90	82.286(2)
γ,°	102.1450(10)	90	74.655(2)
Cell Volume, Å^3	671.40(3)	2744.8(3)	1728.3(2)
Z	2	8	2
D_c, g·cm^{-3}	1.386	1.352	1.479
μ, mm^{-1}	0.099	0.093	0.922
F(000)	292	1168	832
Crystal size, mm	0.25×0.21×0.18	0.22×0.19×0.15	0.16×0.13×0.12
θ limits,°	2.082-25.096	2.388-25.997	1.978-24.997
Reflections collected	8136	45779	32392
Independent reflections	2358 [R_{int} = 0.0173]	2687 [R_{int} = 0.0749]	6265 [R_{int} = 0.0654]
Data / restraints /parameters	2358 / 0 / 191	2687 / 1 / 194	6265 / 102 / 462
Goodness on fit on F^2	1.038	1.108	1.061
R_1 ($I > 2\sigma(I)$)	0.0301	0.0322	0.0838
wR_2 (all data)	0.0790	0.0883	0.2343
Largest diff. peak and hole, e Å^{-3}	0.203 / –0.229	0.255 / –0.257	1.501 / –0.914

Solubility and stability in aqueous media

a) Solubility in D_2O. Each diiron compound was added to a D_2O solution (0.7 mL) of Me_2SO_2 (c = $7.1 \cdot 10^{-3}$ mol·L^{-1}), and the resulting mixture was stirred at 21 °C for 30 minutes. The saturated solution was filtered to remove some solid, and then transferred into an NMR tube and analyzed by ^1H NMR spectroscopy. The concentration (i.e., solubility) was calculated by the relative integral with respect to Me_2SO_2 as internal standard (δ = 3.14 ppm in D_2O). Solubility data in D_2O are compiled in Table 1.

b) Stability in DMSO-d_6/D_2O. The selected iron compound (*ca*. 4 mg) was added to D_2O/DMSO-d_6 mixture (2/1 v/v; 1 mL) containing Me_2SO_2 ($3.47 \cdot 10^{-3}$ M), and the resulting mixture was stirred at room temperature for 15 minutes. The final mixture was filtered over celite, and the filtrated solution was transferred into an NMR tube. The solution was analyzed by ^1H NMR (delay time = 3 s; number of scans = 20), and subsequently heated at 37 °C for 24-72 hours. After cooling to room temperature, the final solution was separated from a minor amount of brown precipitate by celite filtration and then analyzed by ^1H NMR. The amount of starting material in solution (% with respect to the initial spectrum) was calculated by the relative integral with respect to Me_2SO_2 as internal standard.

From [**2**]CF_3SO_3: [**2**]$^+$, [**2'**]$^+$ and aspirin (ratio 7:1:1.7). [**2**]$^+$: ^1H NMR (DMSO-d_6/D_2O = 1:2), δ/ppm = 8.09 (d, $^3J_{H3-H4}$ = 7.8 Hz, 1 H, H^3); 7.84 (t, ^3J = 7.5 Hz, 1 H, H^5); 7.58 (t, ^3J = 7.7 Hz, 1 H, H^4); 7.36 (d, $^3J_{H6-H5}$ = 8.1 Hz, 1 H, H^6); 7.30 (t, ^3J = 7.6 Hz, 1 H, C_6H_3); 7.23 (d, ^3J = 7.3 Hz, 1 H, C_6H_3); 7.10 (d, ^3J = 6.9 Hz, 1 H, C_6H_3); 6.71 (d, ^2J = 14.4 Hz, 1 H, CH_2); 6.57 (d, ^2J = 14.9 Hz, 1 H, CH_2); 5.65, 5.33 (s, 10 H, Cp); 4.42 (s, 1 H, $C_\beta H$); 4.24 (s, 3 H, NMe); 2.18, 1.84 (s, 6 H, C_6H_3*Me*$_2$); 2.08 (s, 3 H, O=CMe). [**2'**]$^+$: ^1H NMR (DMSO-d_6/D_2O = 1:2), δ/ppm = 6.02 (d, ^2J = 15.9 Hz, 1 H, CH_2); 5.82 (d, ^2J = 15.3 Hz, 1 H, CH_2); 5.58, 5.29 (s, 10 H, Cp); 2.26, 1.92 (s, 6 H, C_6H_3*Me*$_2$).

From [**3**]CF_3SO_3: [**3**]$^+$. ^1H NMR (DMSO-d_6/D_2O = 1:2): δ/ppm = 8.30 (dd, $^3J_{H3-H4}$ = 8.0 Hz, $^4J_{H3-H5}$ = 1.6 Hz, 1 H, H^3); 7.83 (td, ^3J = 7.8 Hz, $^4J_{H5-H3}$ = 1.5 Hz, 1 H, H^5); 7.70, 7.51, 7.29 (m, 4 H, H^8 + H^{10} + H^{11} + H^{12}); 7.57 (t, ^3J = 7.7 Hz, 1 H, H^4); 7.37 (d, $^3J_{H6-H5}$ = 8.2 Hz, 1 H, H^6); 5.25, 5.16 (s, 10 H, Cp); 4.62 (s, 1 H, $C_\beta H$); 3.86, 3.29 (s, 6 H, NMe_2); 2.31 (s, 3 H, O=CMe).

From [**4**]CF_3SO_3: [**4**]$^+$. ^{1}H NMR (DMSO-d$_6$/D_2O = 1:2): δ/ppm = 8.22, 7.85-7.00 (m, 11 H, C_6H_3 + C_6H_4); 5.49, 5.29, *5.22*, *4.95* (s, 10 H, Cp); 4.24 (s, 3 H, NMe); 4.17 (s, 1 H, C_βH); 2.23, 2.20, 1.80 (s, 9 H, C_6H_3*Me*$_2$ + O=CMe).

From [**5**]CF_3SO_3: [**5**]$^+$. ^{1}H NMR (DMSO-d$_6$/D_2O = 1:2): δ/ppm = 8.07, 7.80-7.10 (m, 9 H, NH + C_6H_4); 5.19, 5.08 (s, 10 H, Cp); 4.57 (s, 1 H, C_βH); 3.80, 3.24 (s, 6 H, NMe_2); 2.21 (s, 3 H, O=CMe).

From [**6**]CF_3SO_3: [**6**]$^+$. ^{1}H NMR (DMSO-d$_6$/D_2O = 1:2): δ/ppm = 7.89, 7.80-7.00 (m, 12 H, NH + C_6H_3 + C_6H_4); 5.48, 5.27, *5.20*, *4.94* (s, 10 H, Cp); 4.23, *3.74* (s, 3 H, NMe); 4.13 (s, 1 H, C_βH); 2.19, 2.18, 1.80 (s, 9 H, C_6H_3*Me*$_2$ + O=CMe); E/Z ratio = 10.

From [**7**]CF_3SO_3: [**7**]$^+$. ^{1}H NMR (DMSO-d$_6$/D_2O = 1:2), δ/ppm = 8.36, 7.80-7.20 (m, 9 H, NH + C_6H_4); 5.18, 5.08 (s, 10 H, Cp); 4.54 (s, 1 H, C_βH); 3.80, 3.24 (s, 6 H, NMe_2); 2.28 (s, 3 H, O=CMe).

From [**8**]CF_3SO_3: [**8**]$^+$ and [**8**$^{\mathbf{OH}}$]$^+$ + [**8**$^{\mathbf{2OH}}$]$^+$ (ratio 1:5). [**8**]$^+$: ^{1}H NMR (DMSO-d$_6$/D_2O = 1:1), δ/ppm = 7.70-7.50, 7.38 (m, 3 H, H^6 + H^9 + H^{10}); 7.20-6.90 (m, 3 H, H^8 + H^3); 6.73 (m, 2 H, H^2); 5.22, 5.11 (s, 10 H, Cp); 4.80 (s, 1 H, C_βH); 3.81, 3.25 (s, 6 H, NMe_2); 3.70 (m, 8 H, NCH_2 + CH_2Cl); 2.66 (m, 4 H, H^{11} + H^{13}); 1.98 (m, 2 H, H^{12}). [**8**$^{\mathbf{OH}}$]$^+$ + [**8**$^{\mathbf{2OH}}$]$^+$ (overlapped signals): ^{1}H NMR (DMSO-d$_6$/D_2O = 1:1), δ/ppm = 7.65-7.50, 7.39 (m, 3 H, H^6 + H^9 + H^{10}); 7.27, 7.15-6.95 (m, 5 H, H^2 + H^3 + H^8); 5.22, 5.11 (s, 10 H, Cp); 4.80 (s, 1 H, C_βH); 3.81, 3.25 (s, 6 H, NMe_2); 3.56 (m, 8 H, NCH_2 + CH_2OH + CH_2Cl); 2.67 (m, 4 H, H^{11} + H^{13}); 2.00 (m, 2 H, H^{12}).

From [**8**]CF_3SO_3, after 72 h: [**8**$^{\mathbf{2OH}}$]$^+$. ^{1}H NMR (DMSO-d$_6$/D_2O = 1:1), δ/ppm = 7.65-7.50, 7.38 (m, 3 H, H^6 + H^9 + H^{10}); 7.15-7.00, 6.70 (m, 5 H, H^2 + H^3 + H^8); 5.22, 5.11 (s, 10 H, Cp); 4.80 (s, 1 H, C_βH); 3.82, 3.25 (s, 6 H, NMe_2); 3.61, 3.44 (t, $^3J_{HH}$ ≈ 6 Hz, 8 H, NCH_2 + CH_2OH); 2.65 (m, 4 H, H^{11} + H^{13}); 1.97 (m, 2 H, H^{12}).

From [**9**]CF_3SO_3: [**9**]$^+$ and [**9**$^{\mathbf{OH}}$]$^+$ + [**9**$^{\mathbf{2OH}}$]$^+$ (ratio 1:4). [**9**]$^+$: ^{1}H NMR (DMSO-d$_6$/D_2O = 1:1), δ/ppm = 8.13 (s, 1 H, H^6); 7.50 (d, $^3J_{H8-H9}$ = 7.4 Hz, 1 H, H^8); 7.38, 7.34 (m, 2 H, H^9 + H^{10}); 7.10 (d, $^3J_{H3-H2}$ = 8.1 Hz, 2 H, H^3); 6.68 (d, $^3J_{H2-H3}$ = 7.9 Hz, 2 H, H^2); 5.22, 5.10 (s, 10 H, Cp); 3.82, 3.25 (s, 6 H, NMe_2); 3.67 (m, 8 H, NCH_2 + CH_2Cl); 2.56 (m, 2 H, H^{13}); 2.42 (t, $^3J_{H11-H12}$ = 7.1 Hz, 2 H, H^{11}); 1.92 (m, 2 H, H^{12}). [**9**$^{\mathbf{OH}}$]$^+$ + [**9**$^{\mathbf{2OH}}$]$^+$ (overlapped signals): ^{1}H NMR (DMSO-d$_6$/D_2O = 1:1), δ/ppm = 8.13 (s, 1 H, H^6); 7.60-7.05 (m, 7 H, H^8 + H^9 + H^{10} + H^2 + H^3); 5.23, 5.11

(s, 10 H, Cp); 3.82, 3.25 (s, 6 H, NMe_2); 3.55 (m, 8 H, NCH_2 + CH_2Cl + CH_2OH); 2.66 (m, 2 H, H^{13}); 2.45 (m, 2 H, H^{11}); 1.95 (m, 2 H, H^{12}).

From [**10**]CF_3SO_3: [**10**]$^+$ and [**10^{OH}**]$^+$ + [**10^{2OH}**]$^+$ (ratio 1:7). [**10**]$^+$: ^{1}H NMR (DMSO-d_6/D_2O = 1:1), δ/ppm = 7.85-7.50 (m, 4 H, H^7 + H^6); 7.14 (m, 2 H, H^3); 6.73 (m, 2 H, H^2); 5.21, 5.11 (s, 10 H, Cp); 3.81, 3.25 (s, 6 H, NMe_2); 3.70 (m, 8 H, NCH_2 + CH_2Cl); 2.42 (m, 2 H, H^9); 1.94 (m, 2 H, H^{10}). [**10^{OH}**]$^+$ + [**10^{2OH}**]$^+$ (overlapped signals): ^{1}H NMR (DMSO-d_6/D_2O = 1:1), δ/ppm = 7.75-7.55 (m, 4 H, H^7 + H^6); 7.20 (m, 2 H, H^3); 6.92 (m, 2 H, H^2); 5.21, 5.11 (s, 10 H, Cp); 3.82, 3.25 (s, 6 H, NMe_2); 3.61 (m, 8 H, NCH_2 + CH_2Cl + CH_2OH); 2.44 (m, 2 H, H^9); 1.94 (m, 2 H, H^{10}).

From [**10**]CF_3SO_3, after 72 h: [**10^{2OH}**]$^+$. ^{1}H NMR (DMSO-d_6/D_2O = 1:1), δ/ppm = 7.75-7.55 (m, 4 H, H^7 + H^6); 7.09 (d, $^3J_{H3-H2}$ = 8.3 Hz, 2 H, H^3); 6.70 (2, $^3J_{H2-H3}$ = 7.8 Hz, 2 H, H^2); 5.21, 5.11 (s, 10 H, Cp); 3.82, 3.25 (s, 6 H, NMe_2); 3.57 (m, 8 H, NCH_2 + CH_2OH); 2.57 (m, 2 H, H^{11}); 2.43 (m, 2 H, H^9); 1.92 (m, 2 H, H^{10}).

*c) Stability of [**8**]CF_3SO_3 in DMSO/H_2O (HPLC-MS).* Compound [**8**]CF_3SO_3 (2 mg) was dissolved in 0.5 mL of DMSO, and H_2O (0.5 mL) was added. The resulting solution was stirred at 37 °C for variable times, then mass spectra were as follows. After 24 h: [**8**]$^+$ (m/z calcd. for $C_{37}H_{39}Cl_2Fe_2N_2O_4$ 757.0986, found 757.0980) + [**8^{OH}**]$^+$ (m/z calcd. for $C_{37}H_{40}ClFe_2N_2O_5$ 739.1324, found 739.1319) + [**8^{2OH}**]$^+$ (m/z calcd. for $C_{37}H_{41}Fe_2N_2O_6$ 721.1663, found 721.1660), comparable amounts. After 72 h: [**8^{2OH}**]$^+$ (m/z calcd. for $C_{37}H_{41}Fe_2N_2O_6$ 721.1663, found 721.1653). All the isotopic patterns fit well the corresponding calculated ones.

d) Stability in DMSO/cell culture medium. The selected diiron compound (*ca*. 5 mg) was dissolved in DMSO-d_6, then a buffered D_2O solution of DMEM (Dulbecco's Modified Eagle's Medium - low glucose with 1000 mg/L glucose and L-glutamine, without sodium bicarbonate and phenol red) and Me_2SO_2 were added. The resulting mixture was stirred at room temperature for 15 minutes. The final mixture was filtered over celite, and the filtrated solution was transferred into a NMR tube. The solution was analyzed by ^{1}H NMR spectroscopy, and subsequently heated at 37 °C. After cooling to room temperature, the final solution was separated from a minor amount of brown precipitate by celite filtration and then analyzed by ^{1}H NMR spectroscopy. Samples were collected after 24 and 72 hours. From [**3**]CF_3SO_3, after 24 h: [**3**]$^+$, [**3'**]$^+$ and

aspirin (ratio 10:5:3). From [**3**]CF_3SO_3, after 72 h: [**3**]$^+$, [**3'**]$^+$ and aspirin (ratio 10:25:13). From [**5**]CF_3SO_3, after 24 h: [**5**]$^+$, [**5'**]$^+$ and aspirin (ratio 1:10:5). From [**5**]CF_3SO_3, after 72 h: [**5'**]$^+$ and aspirin (ratio 1:1). From [**8**]CF_3SO_3, after 24 h: [**3'**]$^+$ and chlorambucil species ([**8**^{OH}]$^+$ + [**8**^{2OH}]$^+$) (ratio 5:2). From [**8**]CF_3SO_3, after 72 h: [**3'**]$^+$ and chlorambucil species ([**8**^{2OH}]$^+$) (ratio 7:2).

Determination of partition coefficients (Log P_{ow})

Partition coefficients (P_{ow}; IUPAC: K_D partition constant,[7,8] defined as $P_{ow} = c_{org}/c_{aq}$, where c_{org} and c_{aq} are the molar concentrations of the selected compound in the organic and aqueous phases, respectively, were determined by the shake-flask method and UV-Vis measurements.[9] Values of Log P_{ow} for diiron complexes are compiled in Table 2. All the operations were carried out at 21±1 °C. De-ionised water and 1-octanol were mixed and vigorously stirred for 24 hours at room temperature to allow saturation of both phases, then separated by centrifugation and used for the following experiments. A solution of the selected Fe compound in the organic phase (V = 5 mL) was prepared and its UV-Vis spectrum was recorded. An aliquot of the solution (V_{org} = 1.5 mL) was then transferred into a test tube and the aqueous phase ($V_{org} = V_{aq}$ = 1.5 mL) was added. The mixture was vigorously stirred for 20 minutes and the resulting emulsion was centrifuged (5000 rpm, 10’). Hence, the UV-Vis spectrum of the organic phase was recorded. The procedure was repeated three times for each compound. The partition coefficient was then calculated as $P_{ow} = \frac{A_{org}}{A_{0,org} - A_{org}}$, where $A_{0,org}$ and A_{org} are the absorbance values in the organic phase, respectively before and after mixing with the aqueous phase.[9] UV-Vis measurements were carried out using 1 cm PMMA cuvettes. The wavelength of the maximum absorption of each compound was used for UV-Vis quantification.

Figure S1. Stern-Volmer plots for the fluorescence emission changes observed upon addition of [**4**]CF_3SO_3 or [**9**]CF_3SO_3 to BSA. C_{BSA} = 3.34×10^{-7} M, $C_{[4]}$ from 0 to 1.73×10^{-5} M, $C_{[9]}$ from 0 to 6.89×10^{-6} M, NaCl 0.1M, NaCac 0.01 M, pH 7.0, λ_{ex} = 280 nm, λ_{em} = 345 nm, 25°C.

The Stern-Volmer equation used is:

$$\frac{F°}{F} = 1 + K_{SV}[Q]$$

where F° is the fluorescence read in the absence of added quencher, F is read at each addition (corrected for dilution effects), K_{SV} is the Stern-Volmer constant and [Q] is the quencher (metal complex) concentration.

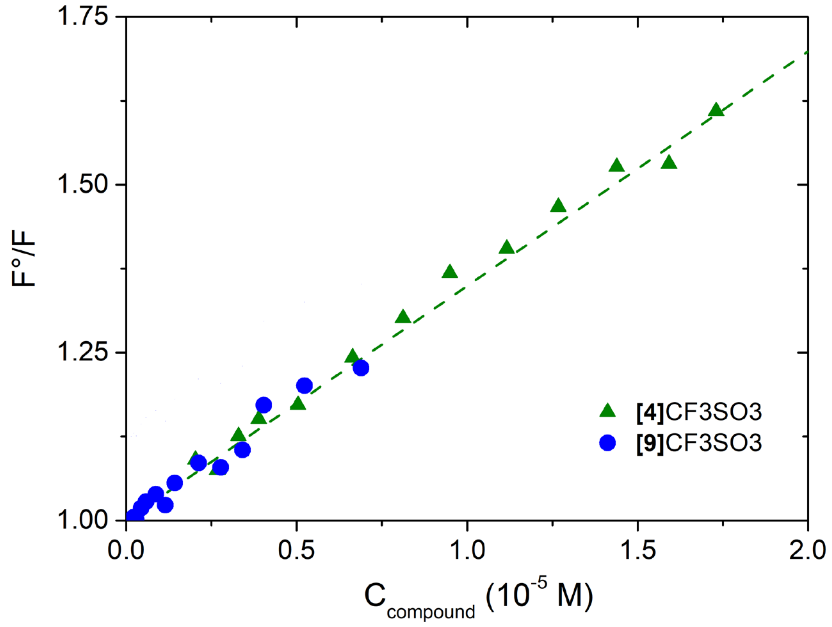

Figure S2. HypSpec2014 analysis (http://www.hyperquad.co.uk) of the fluorescence emission changes observed upon addition of [**4**]CF_3SO_3 (upper panel) or [**9**]CF_3SO_3 (bottom panel) to BSA.

For both panels: on the left, left titration curve (open diamond = experimental, cross = calculated) and species distribution (green = free BSA, blue = compound/BSA adduct); on the right, fluorescence emission spectrum (open diamond = experimental, dashed red line = calculated) and relevant spectrum deconvolution (green = free BSA, blue = compound/BSA adduct). The bottom panels are the residuals. C_{BSA} = 3.34×10^{-7} M, $C_{[4]}$ from 0 to 1.73×10^{-5} M, $C_{[9]}$ from 0 to 6.89×10^{-6} M, NaCl 0.1M, NaCac 0.01 M, pH 7.0, λ_{ex} = 280 nm, 25°C.

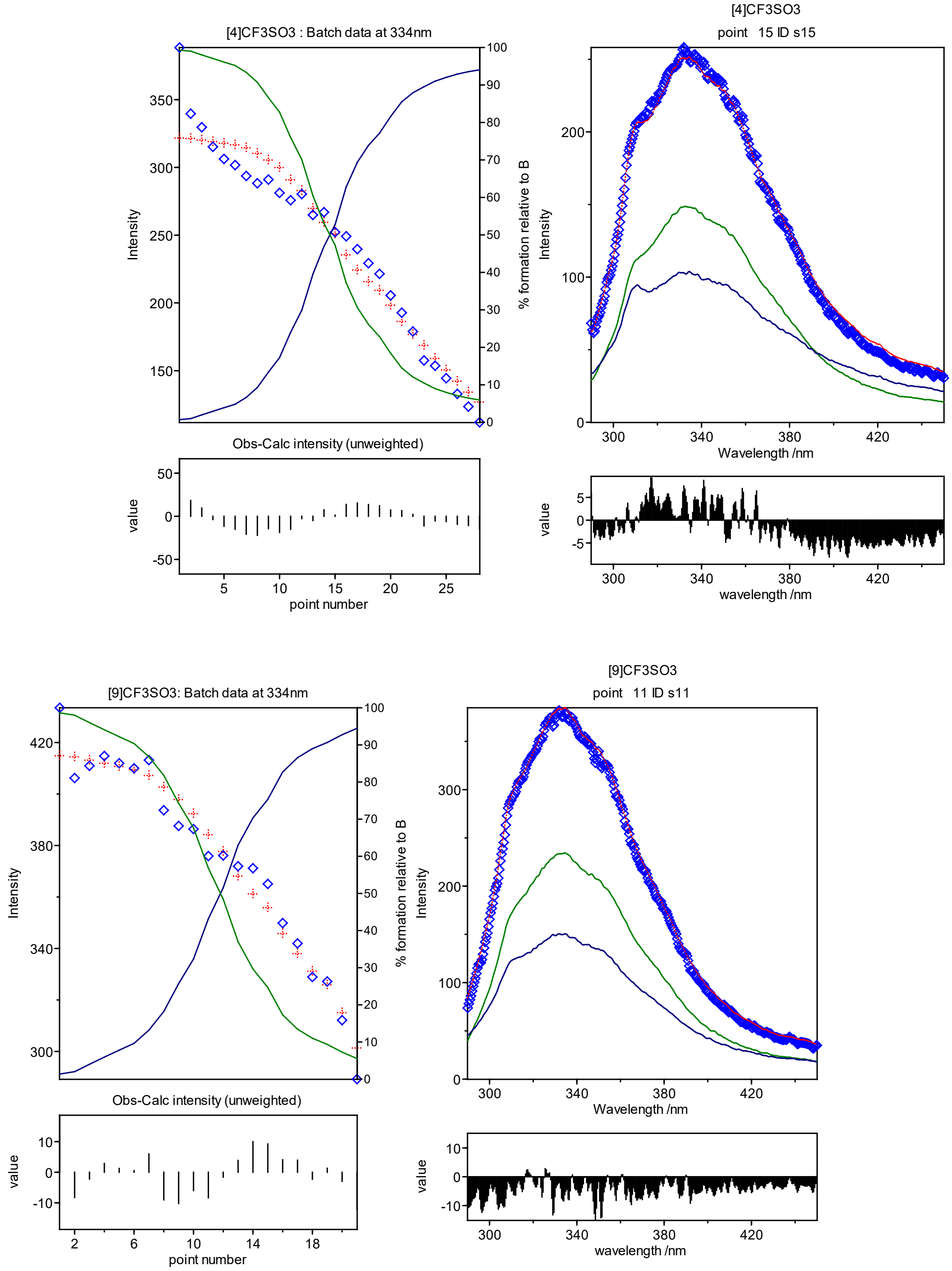

Figure S3. ^{1}H NMR spectrum (401 MHz, $CDCl_3$) of **ALK^{A1}**.

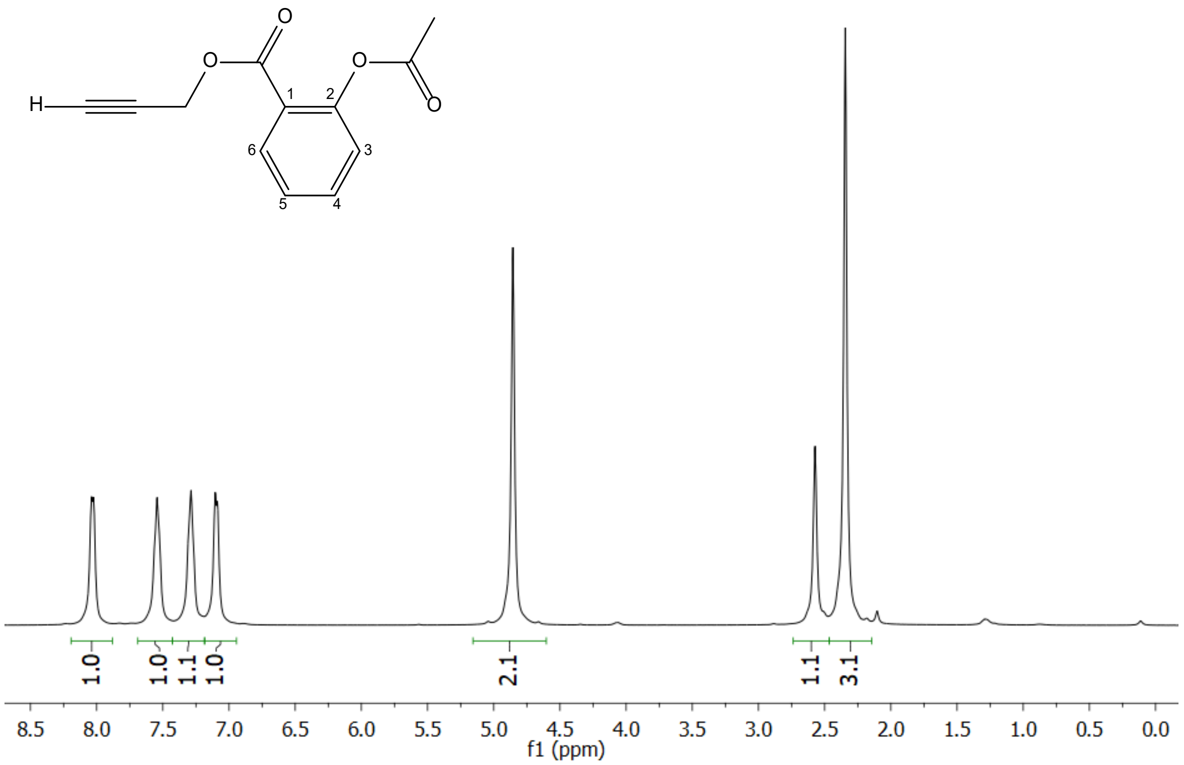

Figure S4. ^{1}H NMR spectrum (401 MHz, $CDCl_3$) of **ALK^{A2}**.

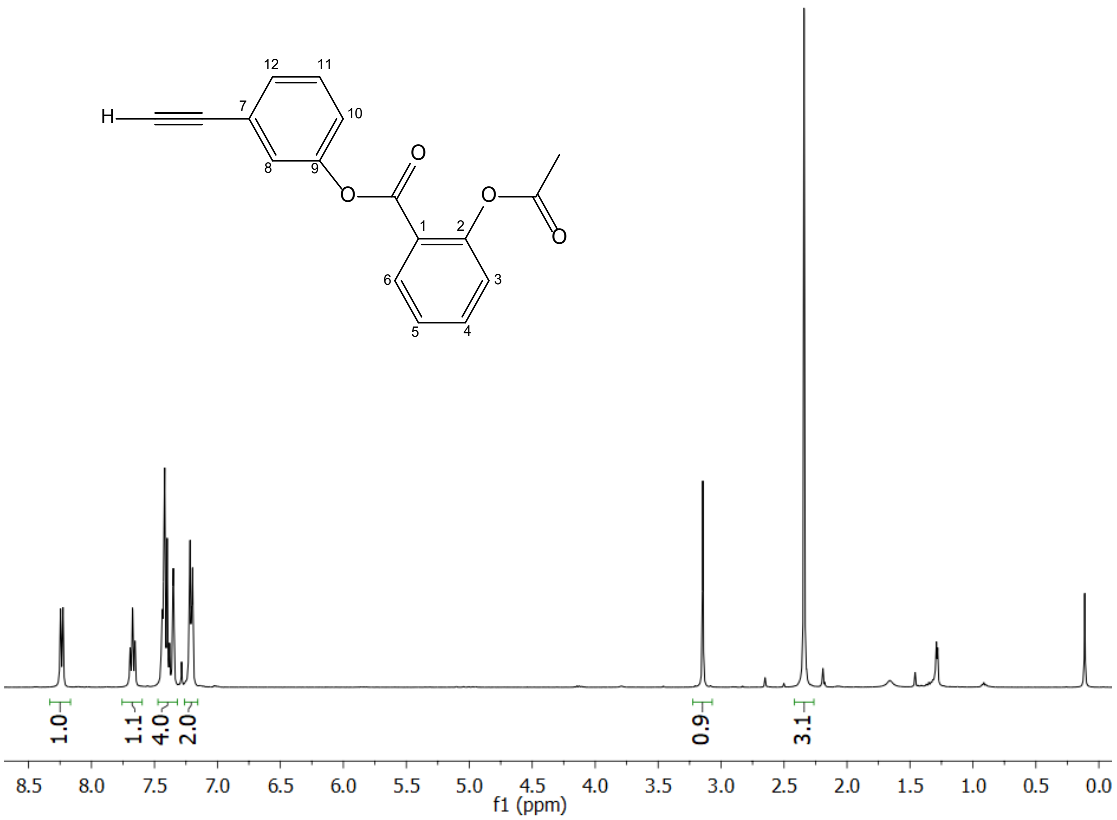

Figure S5. $^{13}C\{^{1}H\}$ NMR spectrum (101 MHz, $CDCl_3$) of **ALK^{A2}**.

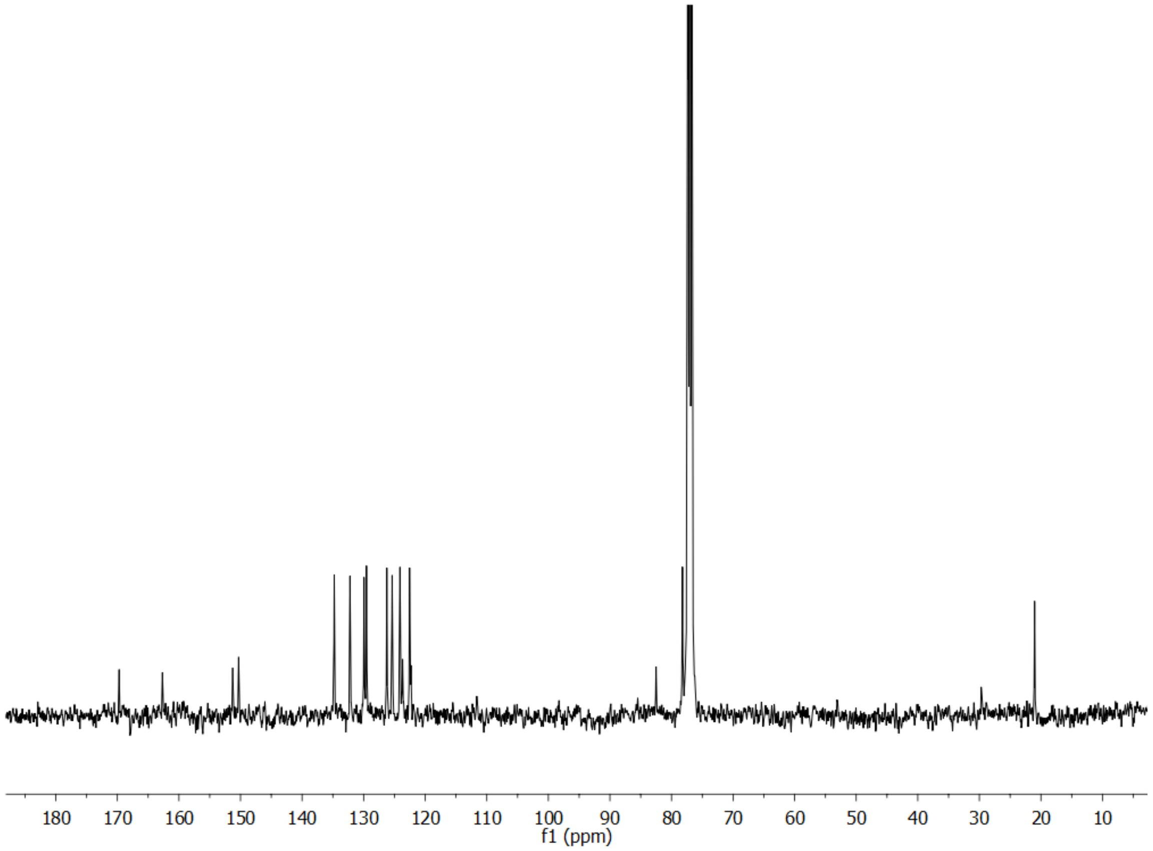

Figure S6. ^{1}H NMR spectrum (401 MHz, $CDCl_3$) of **ALK^{A3}**.

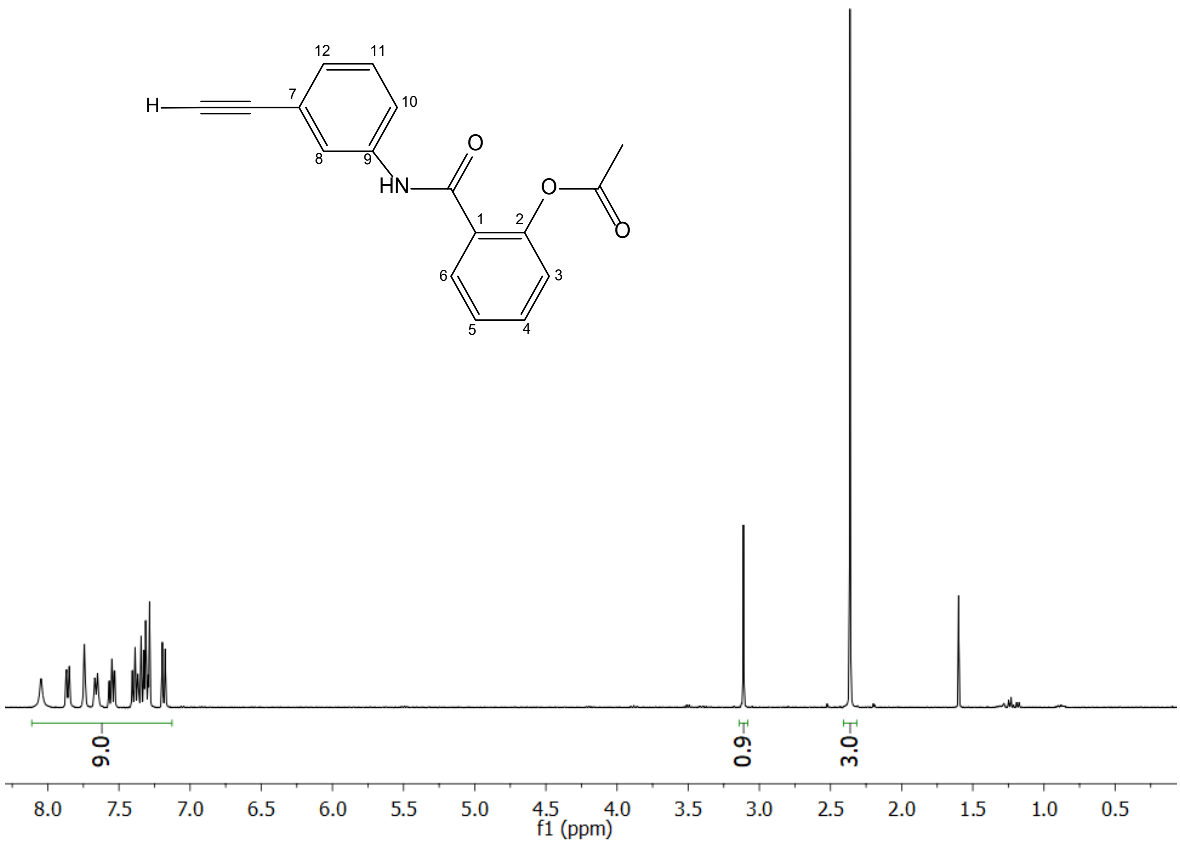

Figure S7. $^{13}C\{^{1}H\}$ NMR spectrum (101 MHz, $CDCl_3$) of **ALK^{A3}**.

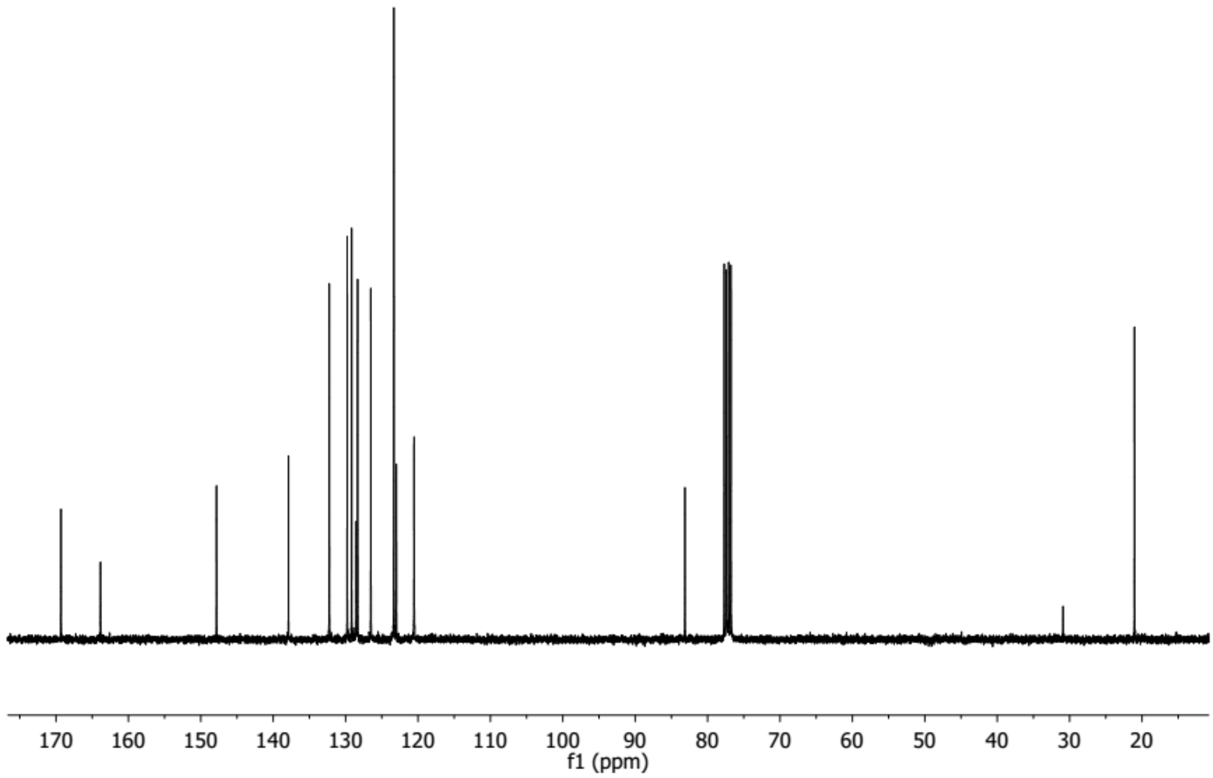

Figure S8. ^{1}H NMR spectrum (401 MHz, $CDCl_3$) of **ALK^{A4}**.

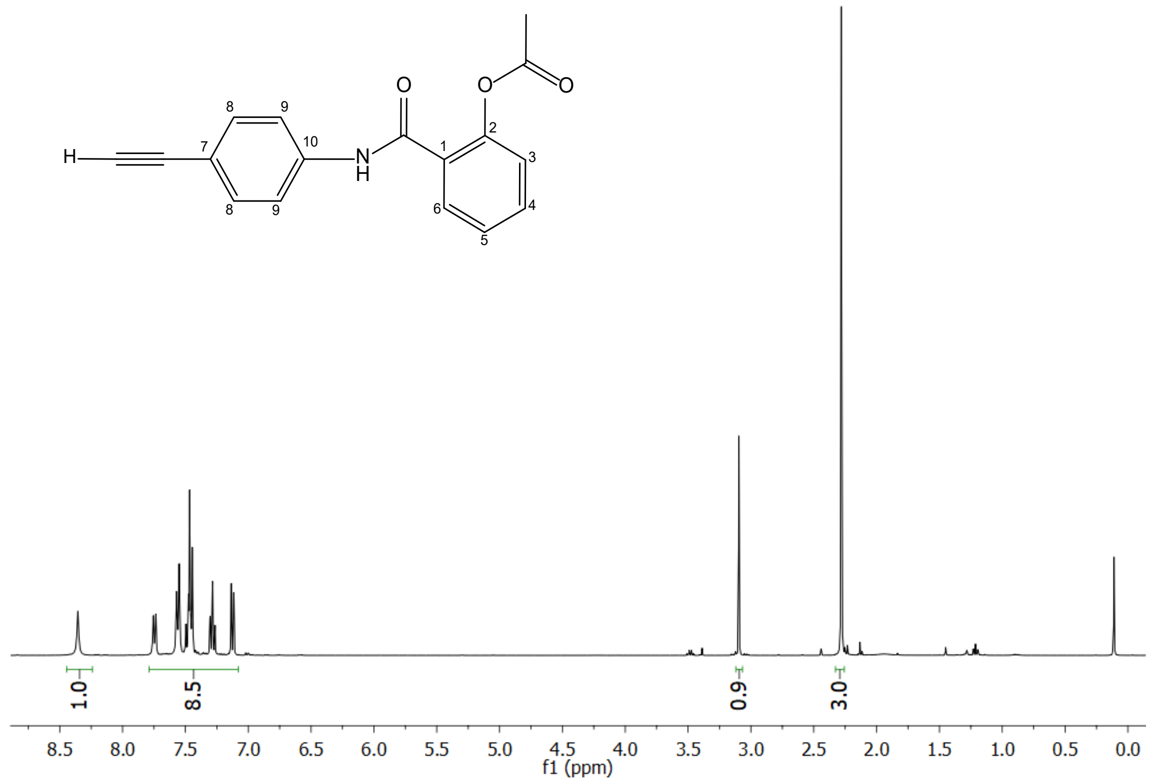

Figure S9. ^{13}C{^{1}H} NMR spectrum (101 MHz, $CDCl_3$) of **ALK^{A4}**.

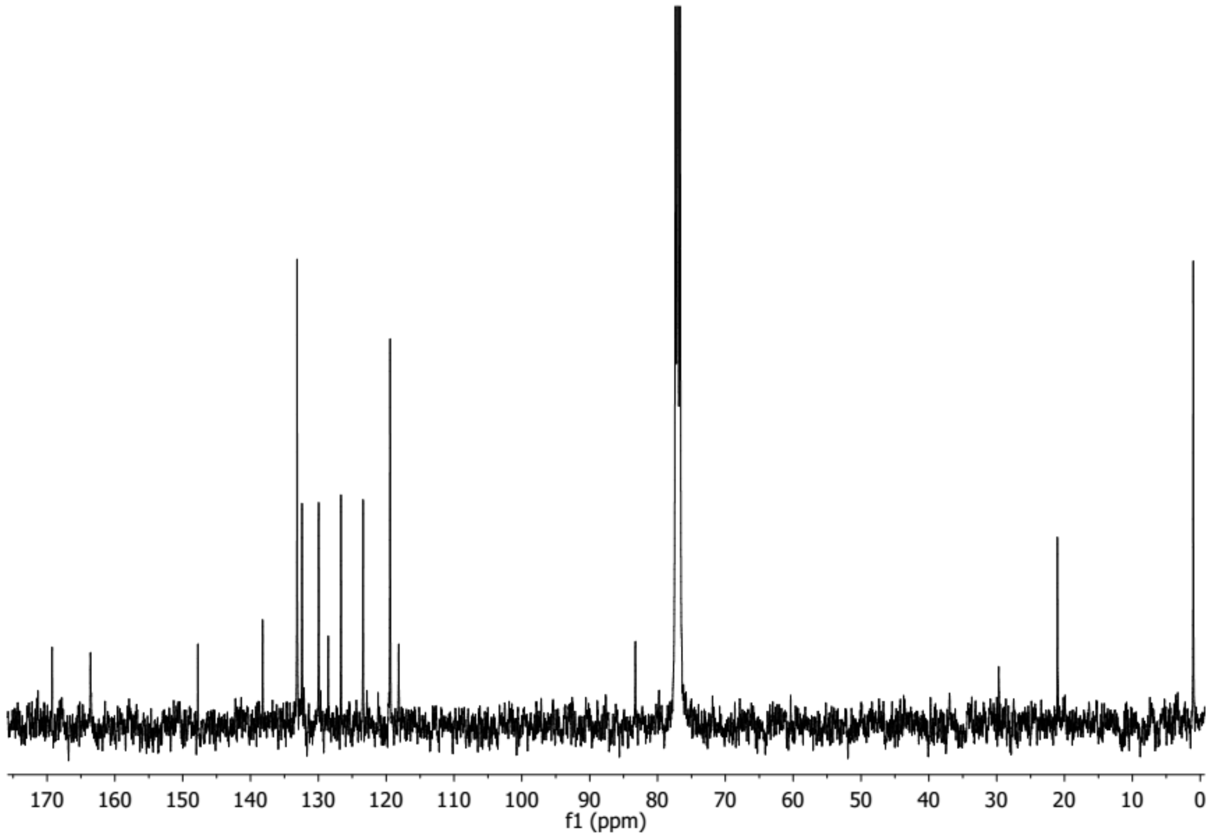

Figure S10. ^{1}H NMR spectrum (401 MHz, $CDCl_3$) of **ALK^{C1}**.

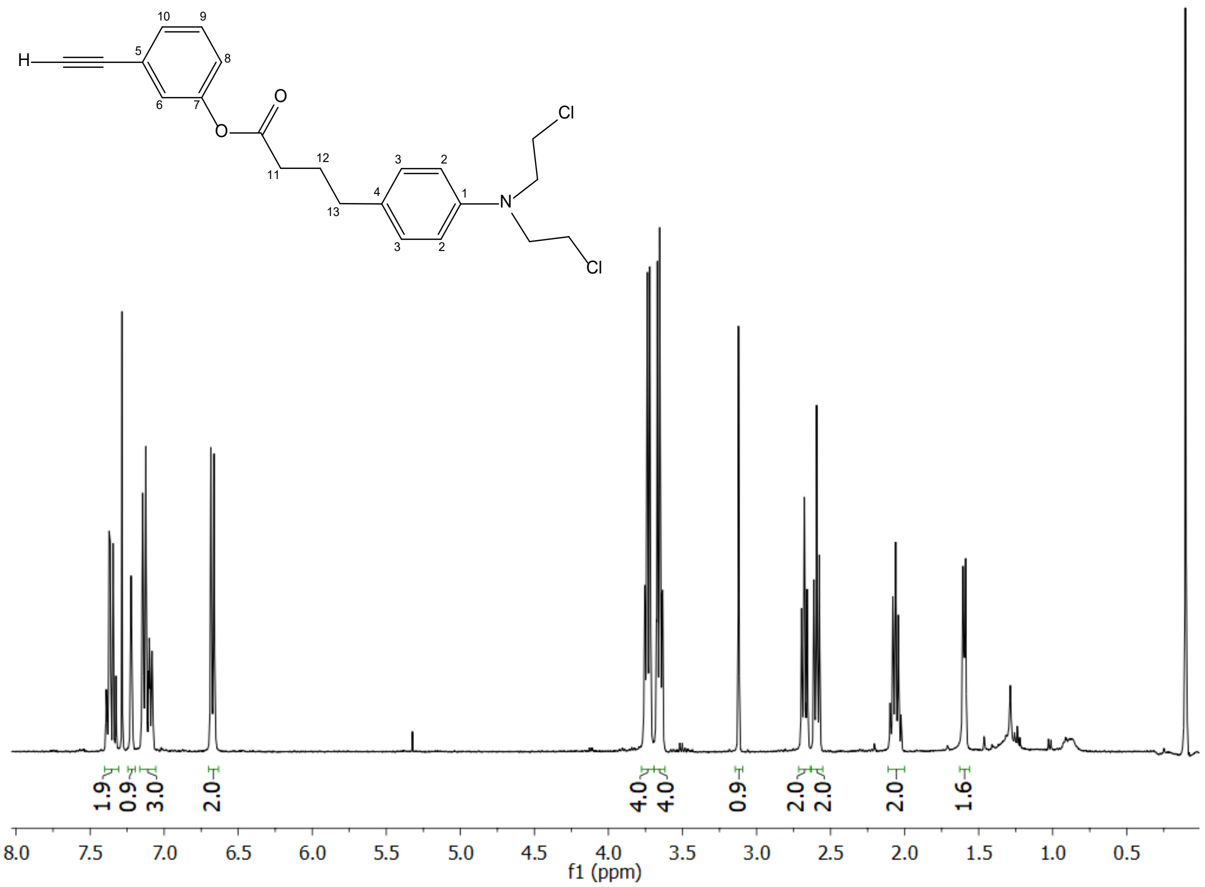

Figure S11. $^{13}C\{^{1}H\}$ NMR spectrum (101 MHz, $CDCl_3$) of **ALK^{C1}**.

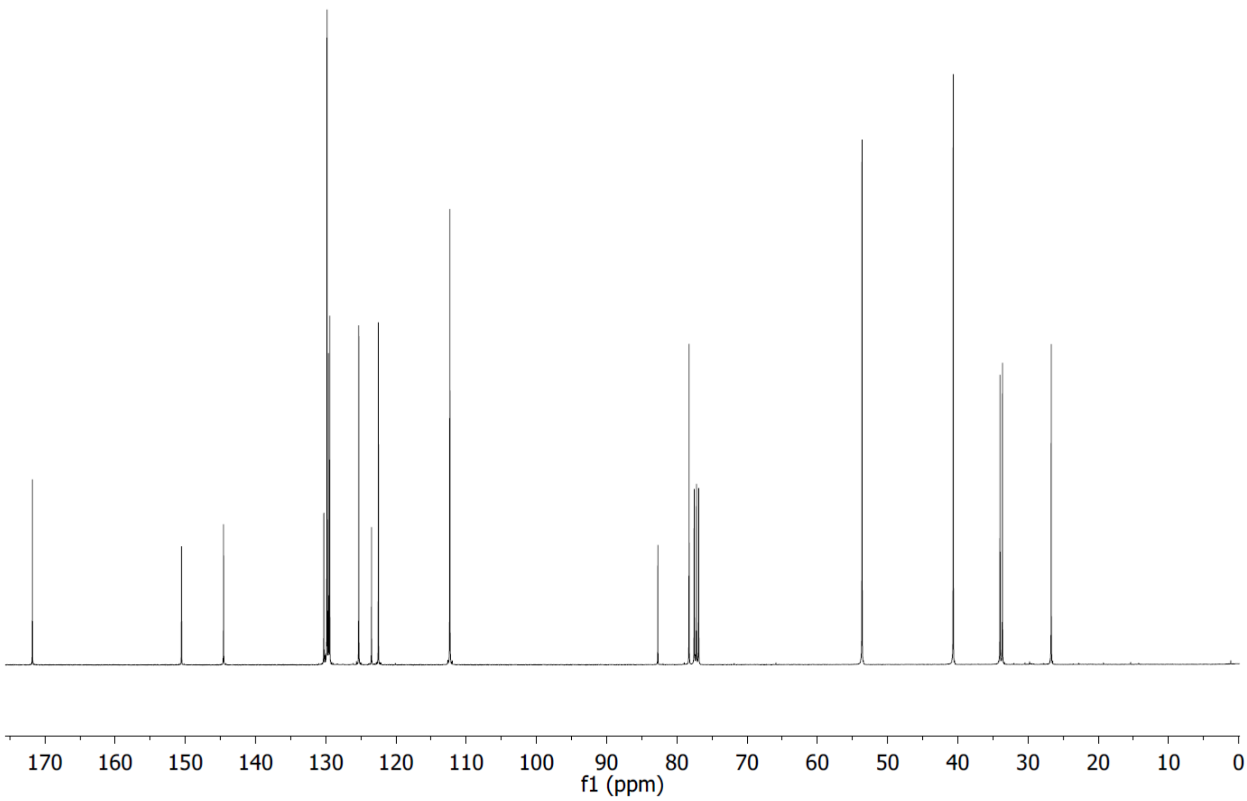

Figure S12. ^{1}H NMR spectrum (401 MHz, $CDCl_3$) of **ALK^{C2}**.

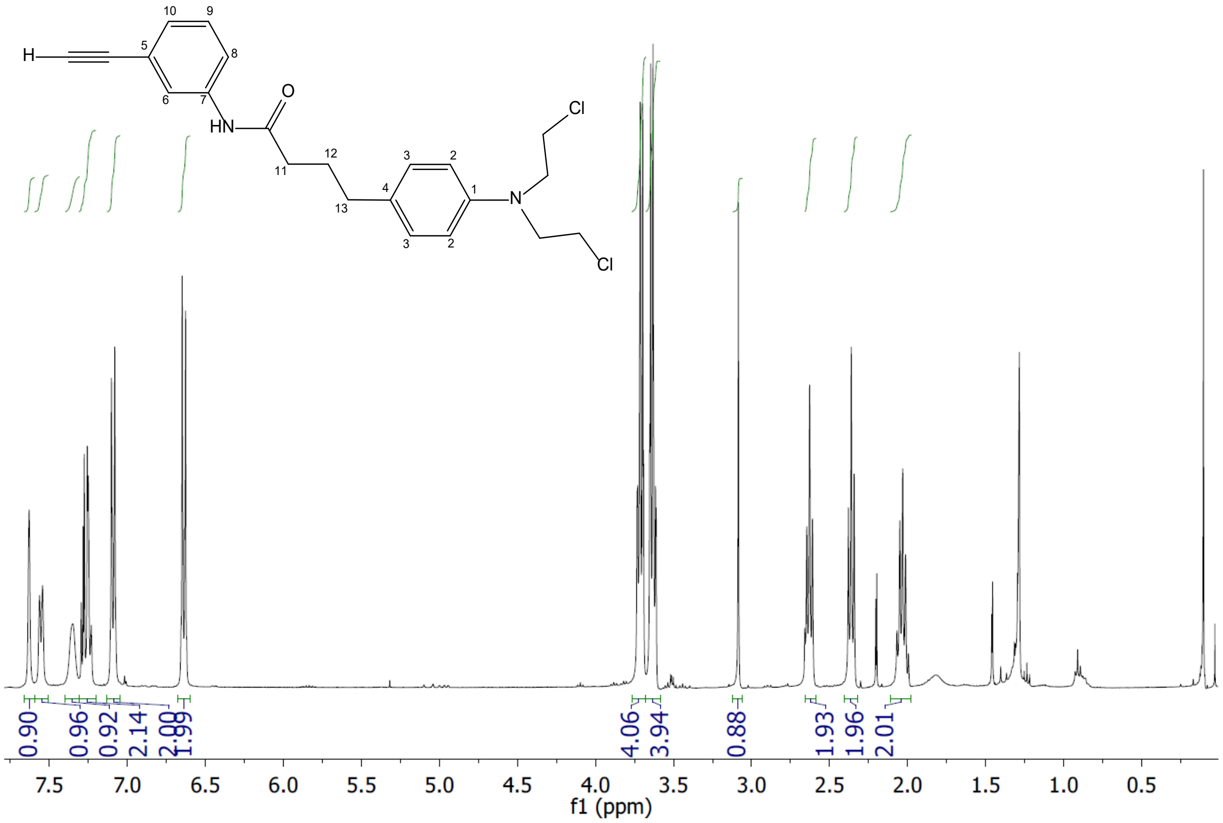

Figure S13. $^{13}C\{^{1}H\}$ NMR spectrum (101 MHz, $CDCl_3$) of **ALK^{C2}**.

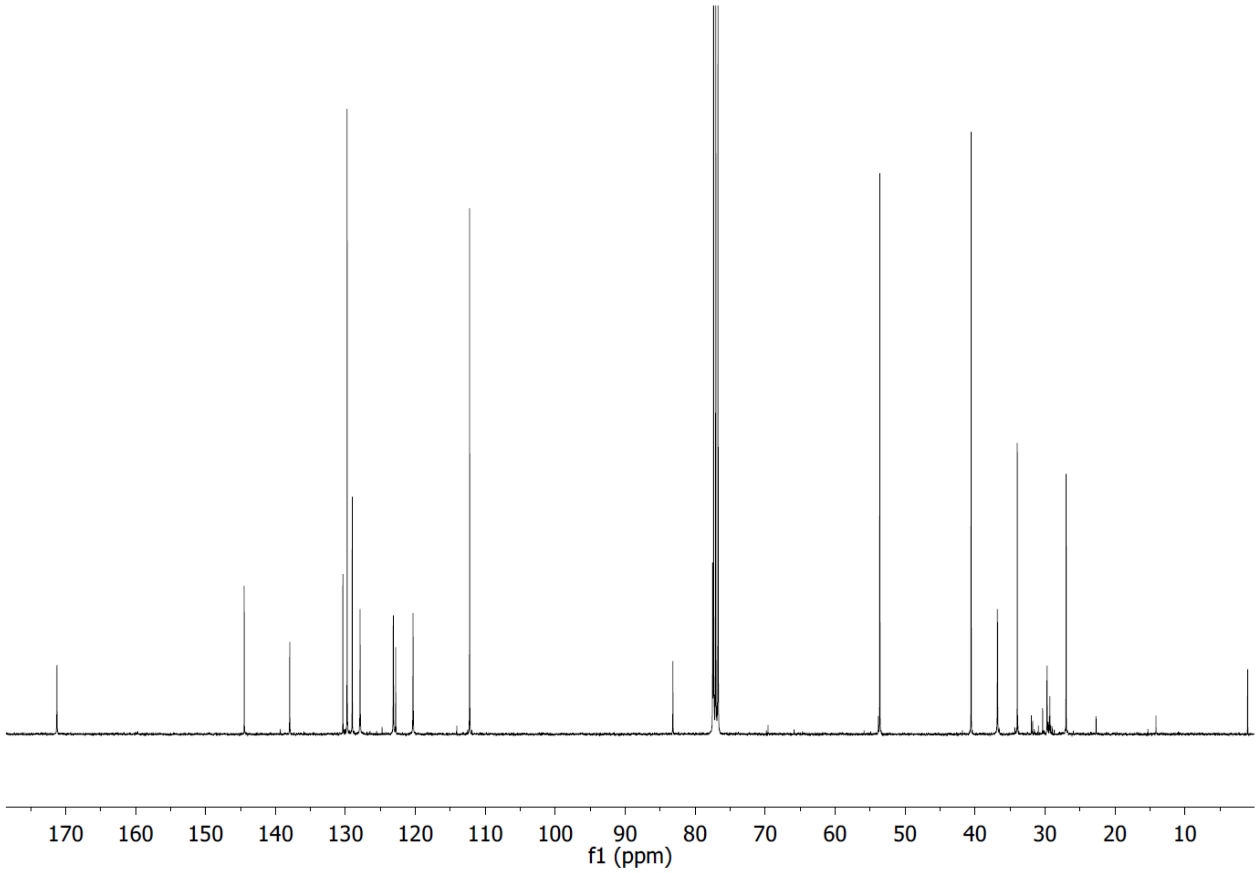

Figure S14. ^{1}H NMR spectrum (401 MHz, acetone-d_6) of **ALK^{C3}**.

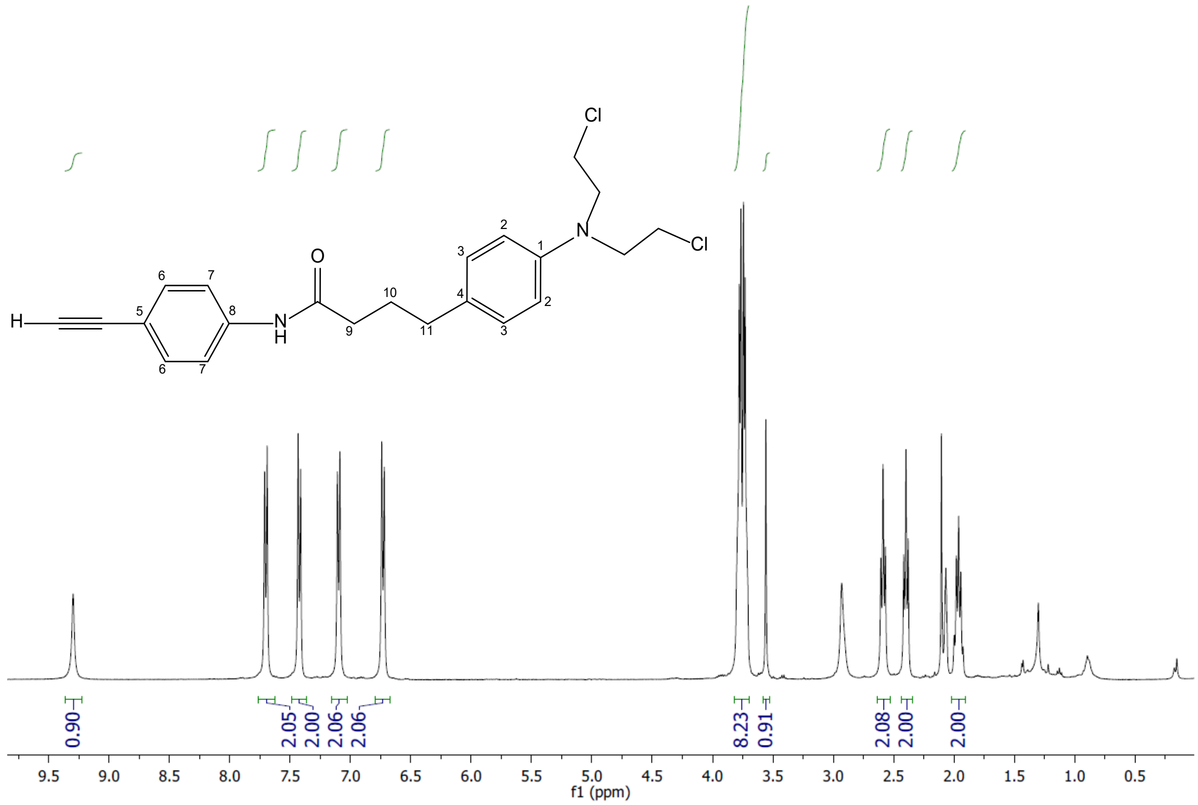

Figure S15. $^{13}C\{^{1}H\}$ NMR spectrum (101 MHz, acetone-d_6) of **ALK^{C3}**.

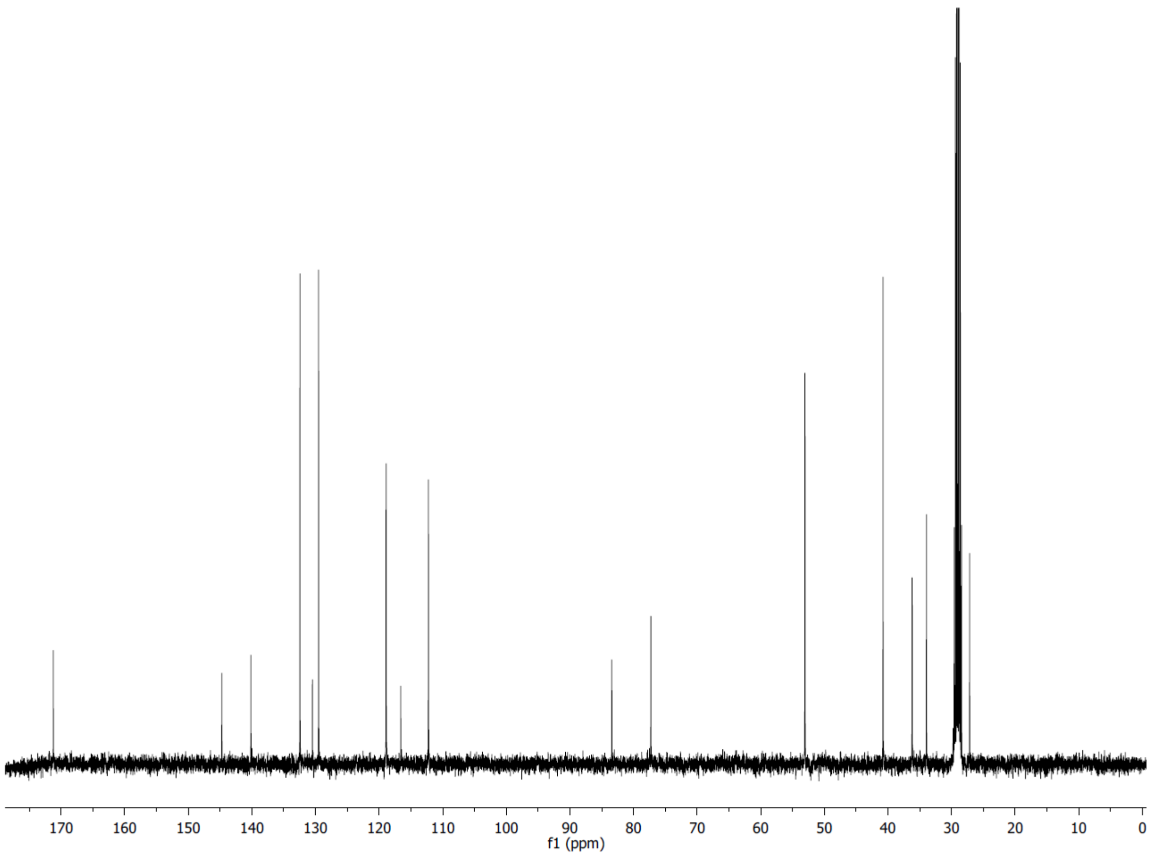

Figure S16. ^{1}H NMR spectrum (401 MHz, $CDCl_3$) of [**2**]CF_3SO_3.

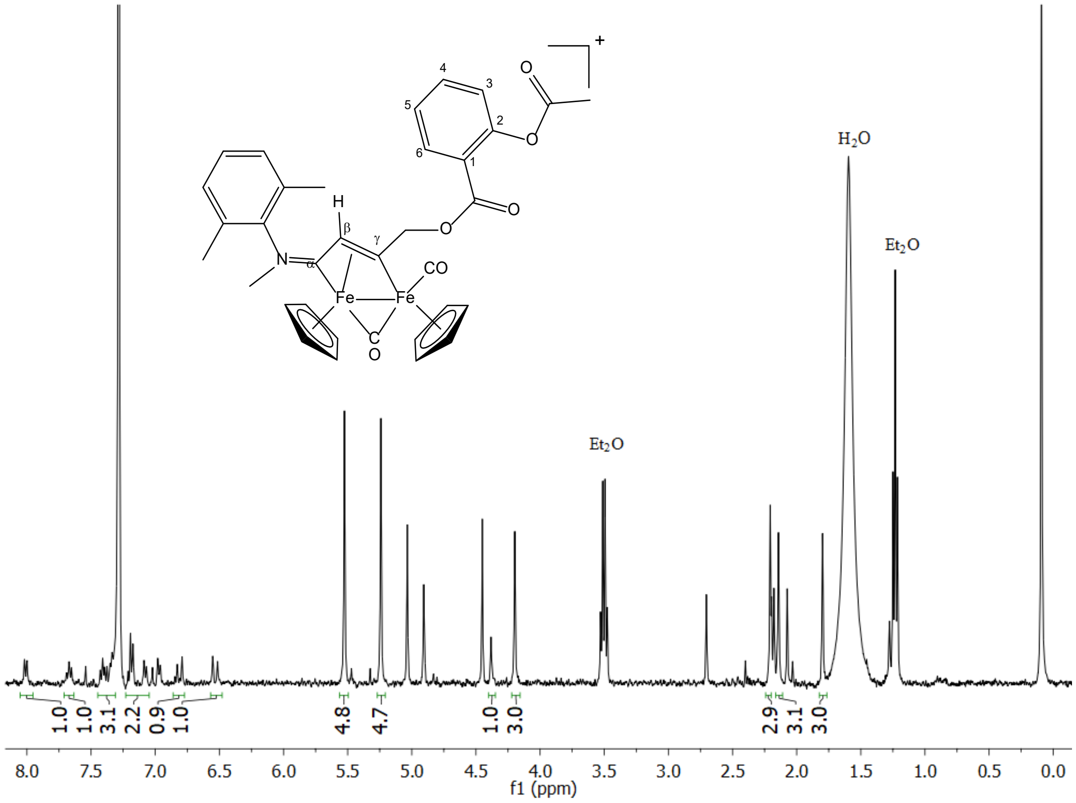

Figure S17. $^{13}C\{^{1}H\}$ NMR spectrum (101 MHz, acetone-d_6) of [**2**]CF_3SO_3.

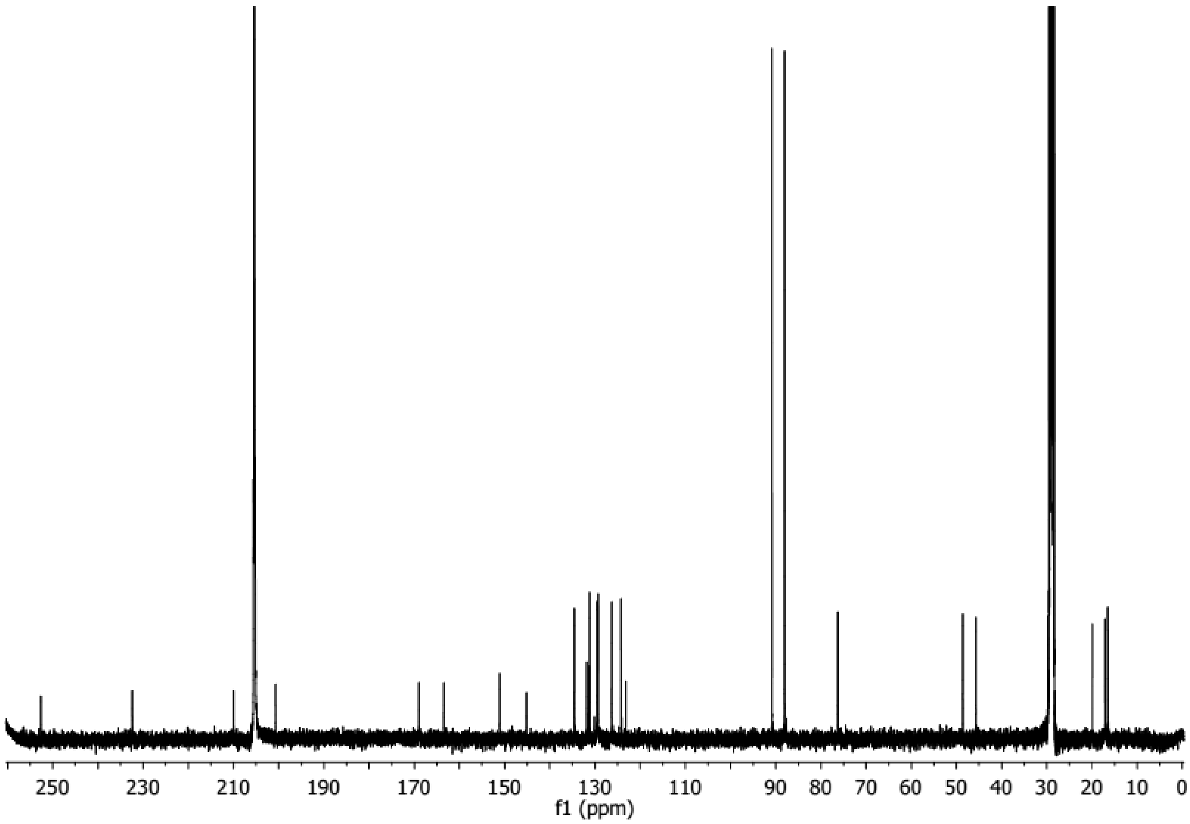

Figure S18. ^{1}H NMR spectrum (401 MHz, D_2O/DMSO) of [**2**]CF_3SO_3.

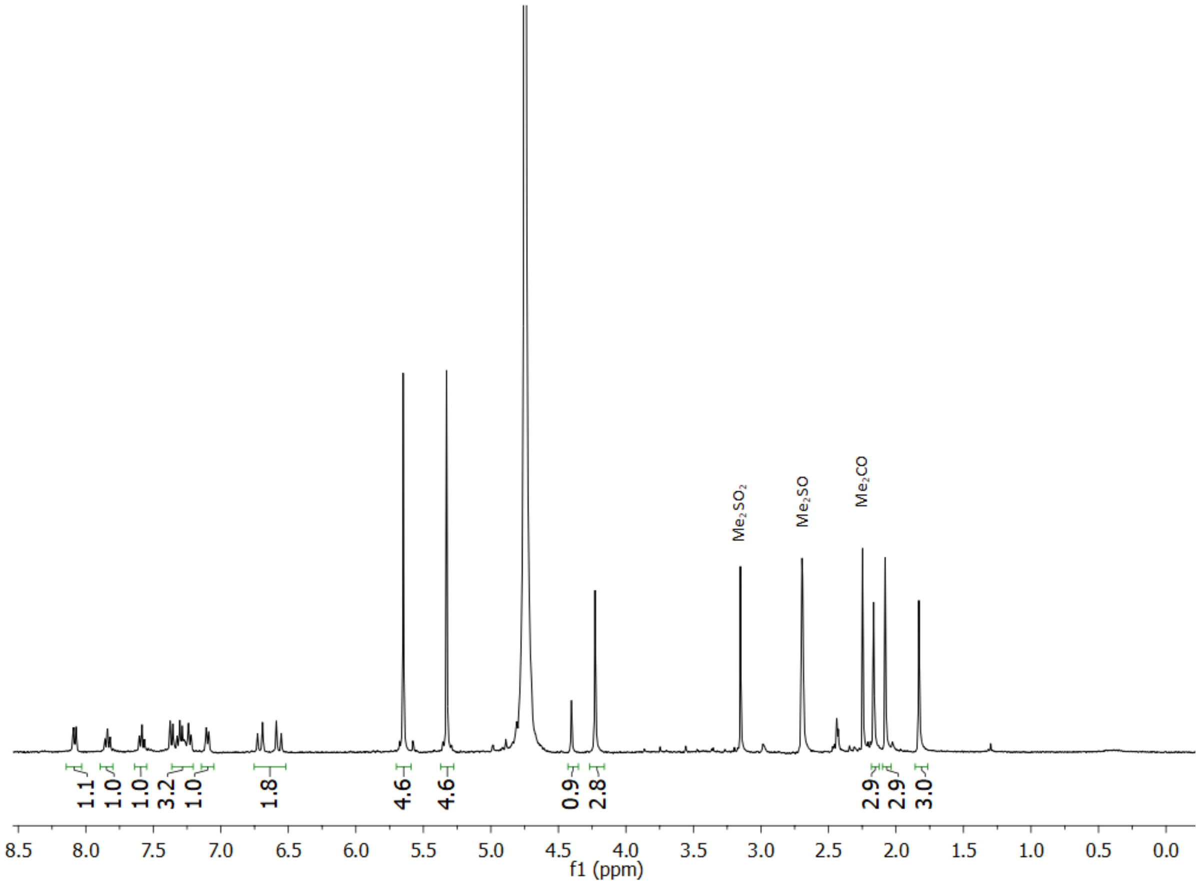

Figure S19. ^{1}H NMR spectrum (401 MHz, $CDCl_3$) of [**3**]CF_3SO_3.

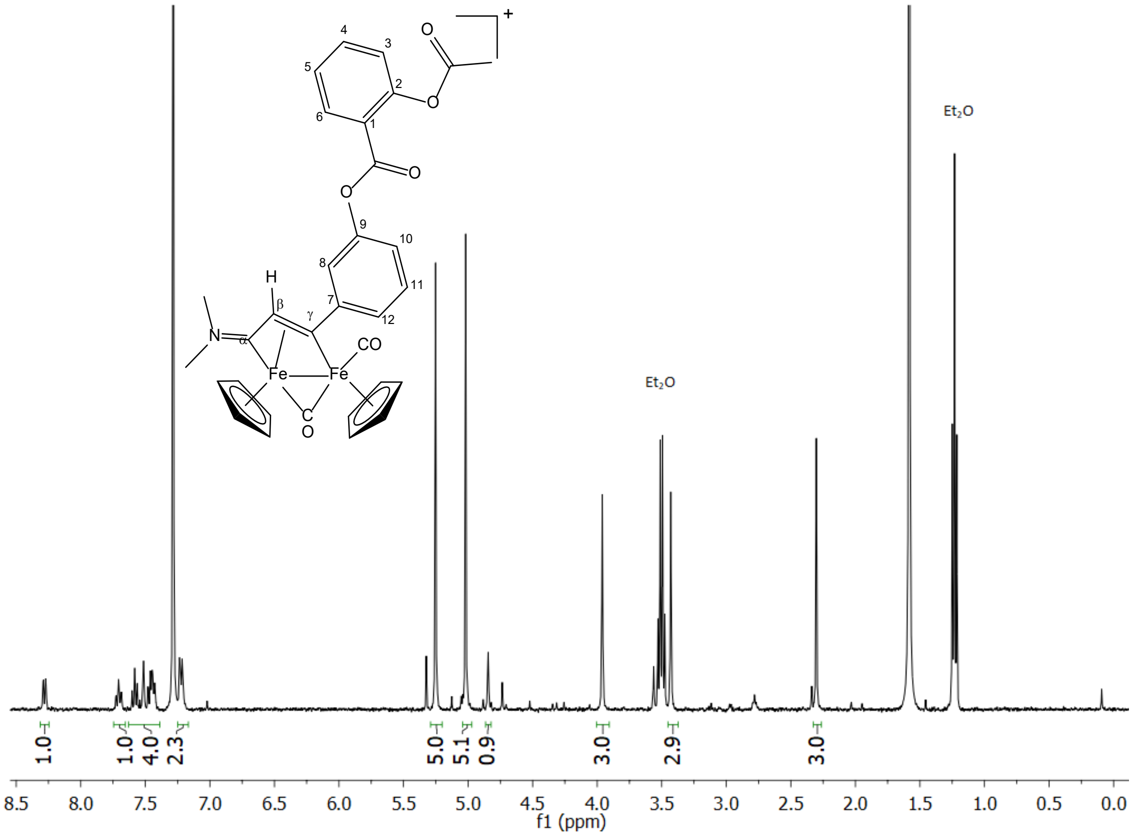

Figure S20. $^{13}C\{^{1}H\}$ NMR spectrum (101 MHz, $CDCl_3$) of [**3**]CF_3SO_3.

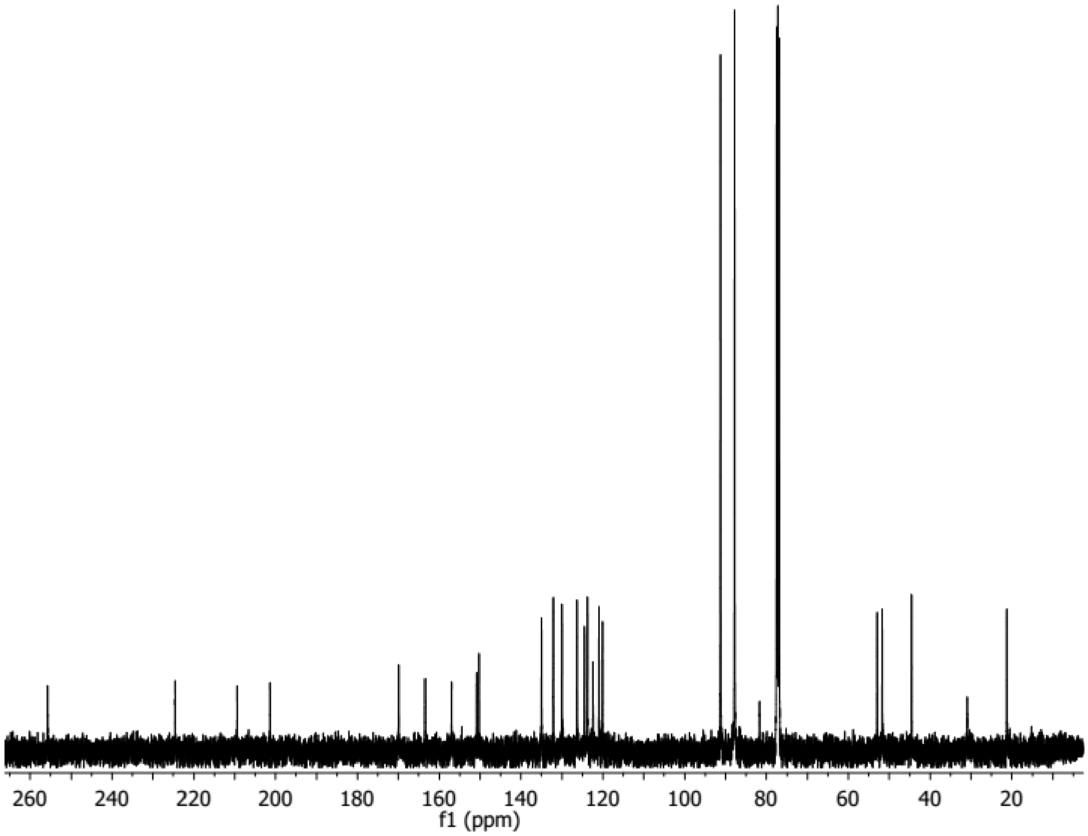

Figure S21. ^{1}H NMR spectrum (401 MHz, D_2O/DMSO) of [**3**]CF_3SO_3.

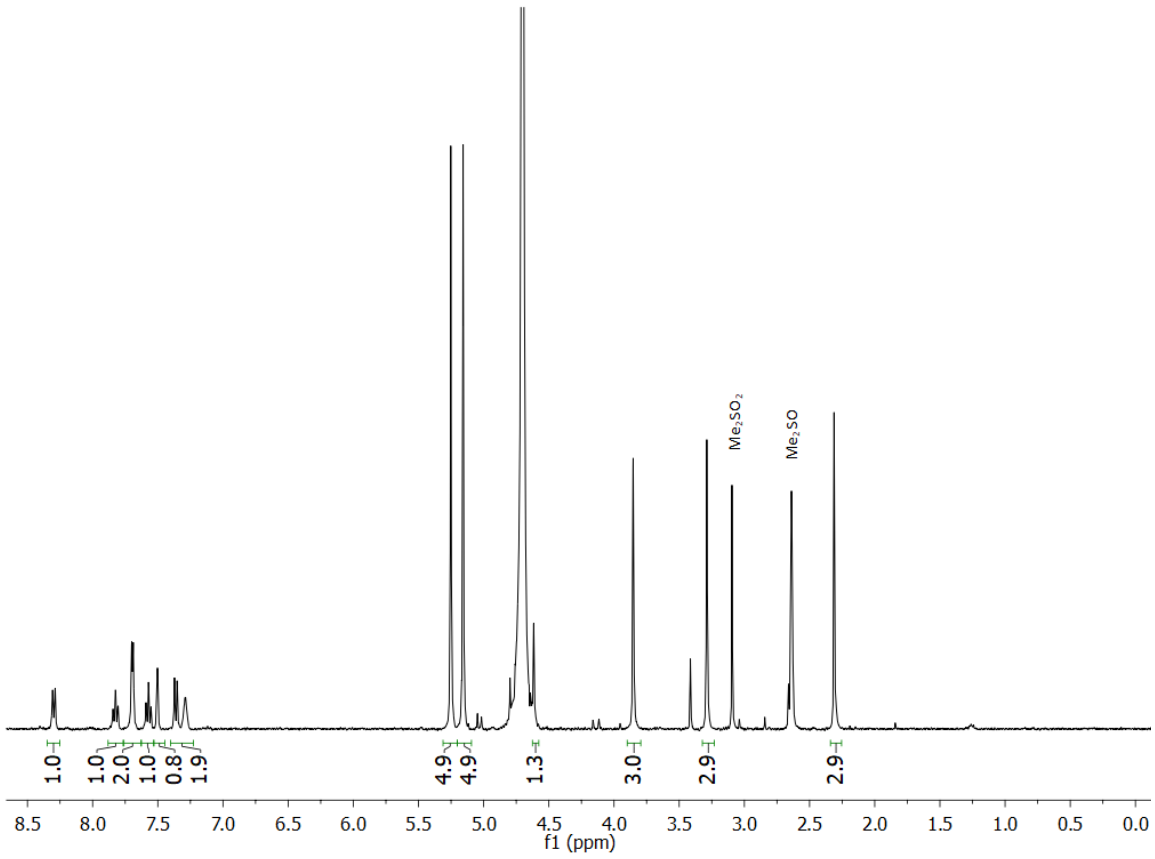

Figure S22. ^{1}H NMR spectrum (401 MHz, acetone-d$_6$) of [**4**]CF_3SO_3.

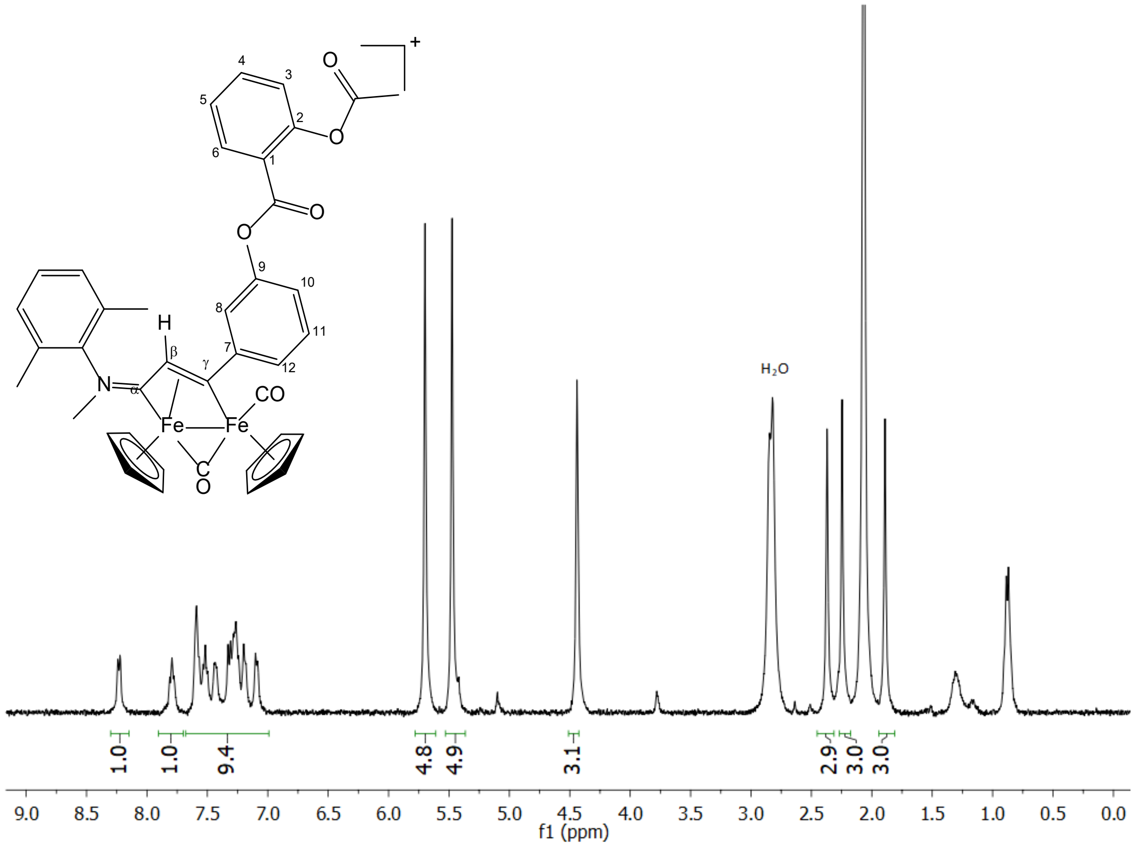

Figure S23. $^{13}C\{^{1}H\}$ NMR spectrum (101 MHz, acetone-d$_6$) of [**4**]CF_3SO_3.

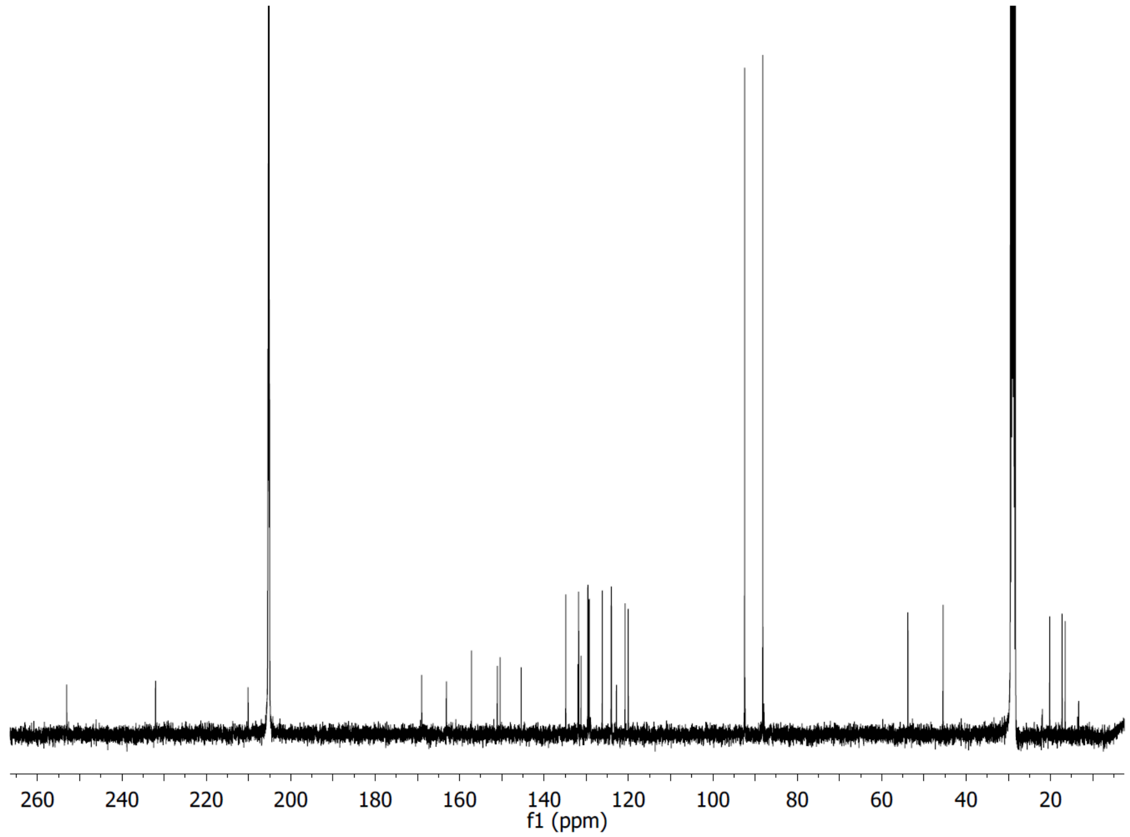

Figure S24. ^{1}H NMR spectrum (401 MHz, D_2O/DMSO) of [**4**]CF_3SO_3.

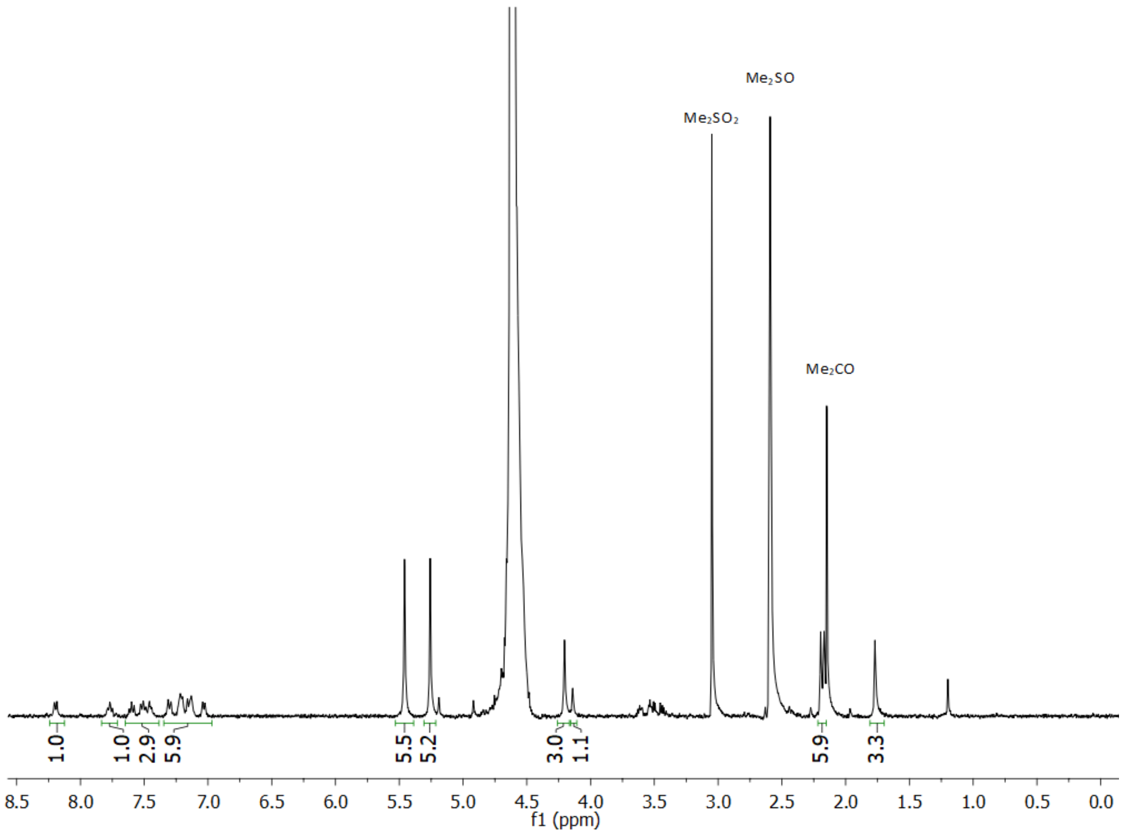

Figure S25. ^1H NMR spectrum (401 MHz, acetone-d$_6$) of [**5**]CF_3SO_3.

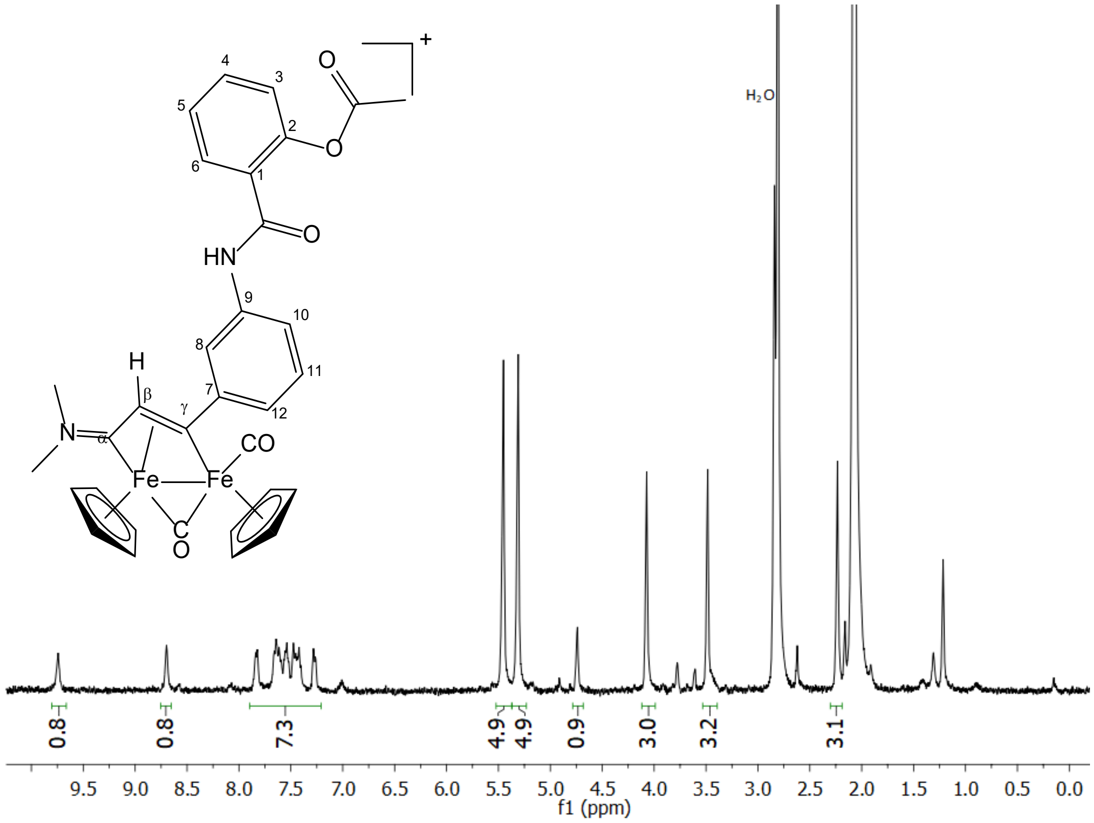

Figure S26. $^{13}C\{^1H\}$ NMR spectrum (101 MHz, acetone-d$_6$) of [**5**]CF_3SO_3.

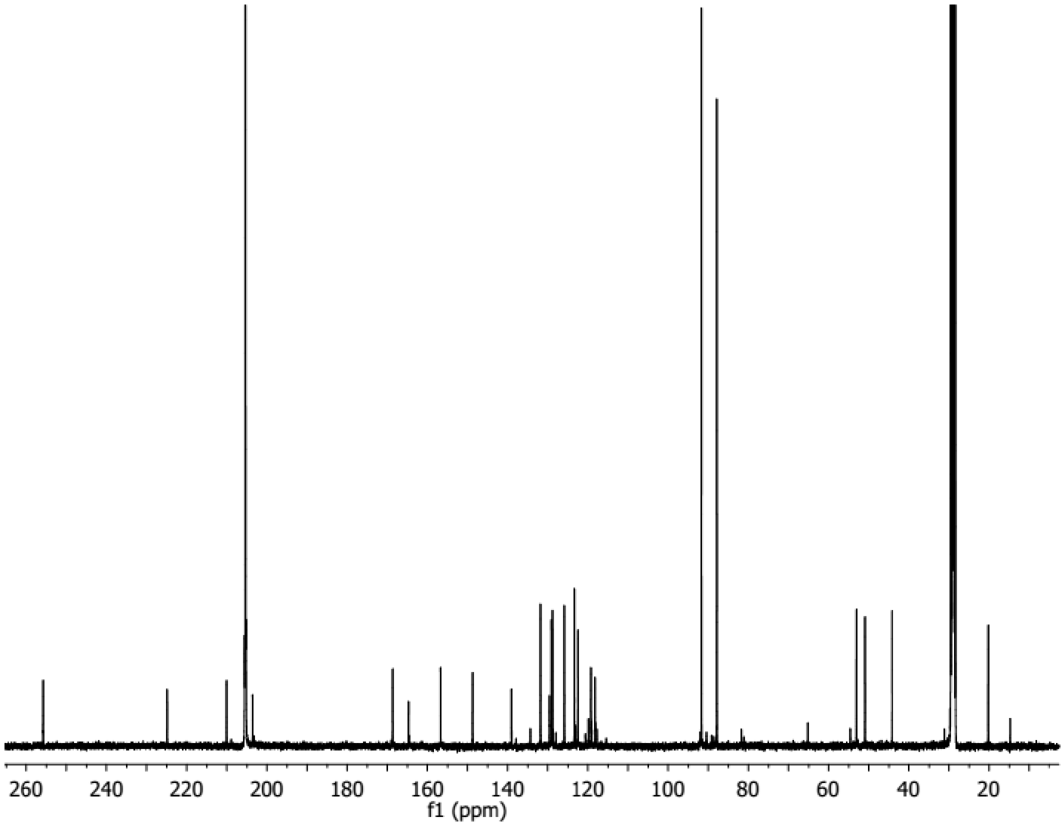

Figure S27. ^{1}H NMR spectrum (401 MHz, D_2O/DMSO) of [**5**]CF_3SO_3.

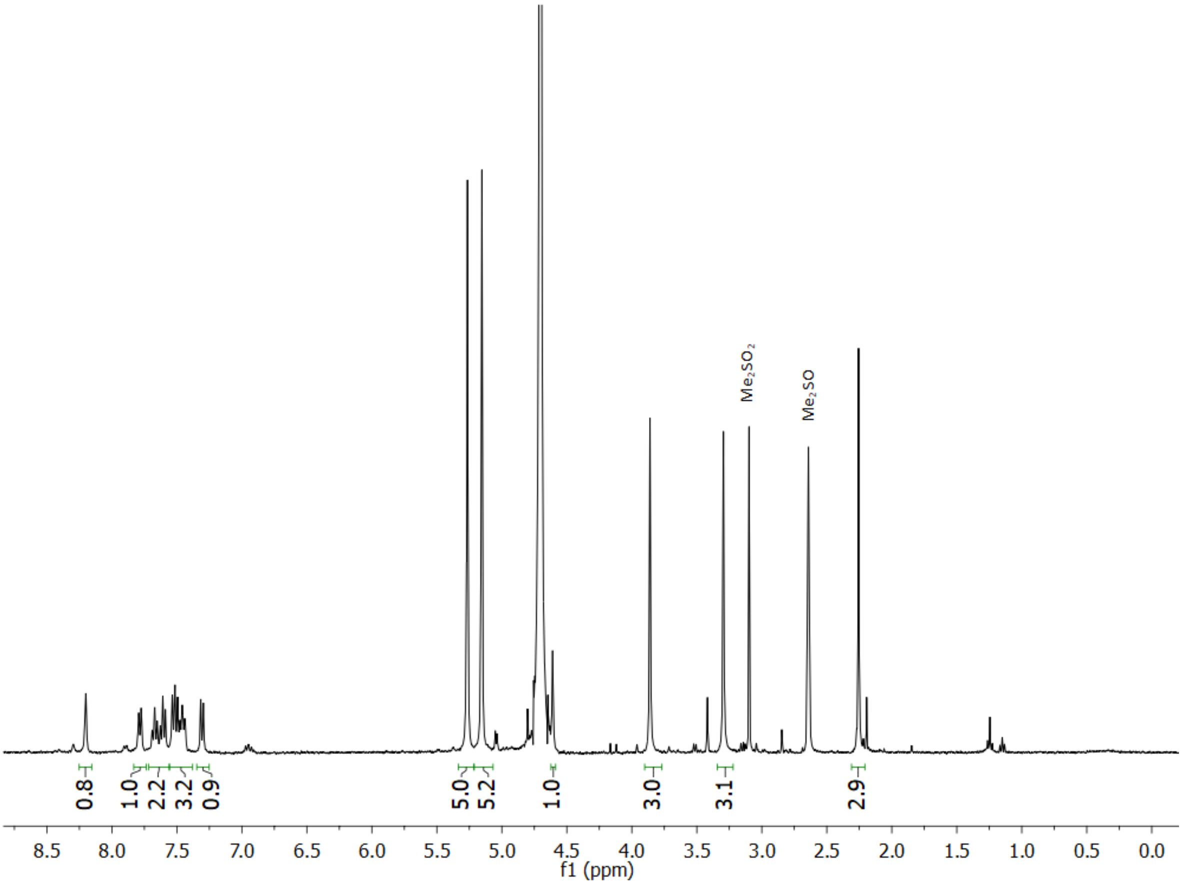

Figure S28. ^{1}H NMR spectrum (401 MHz, acetone-d$_6$) of [**6**]CF_3SO_3.

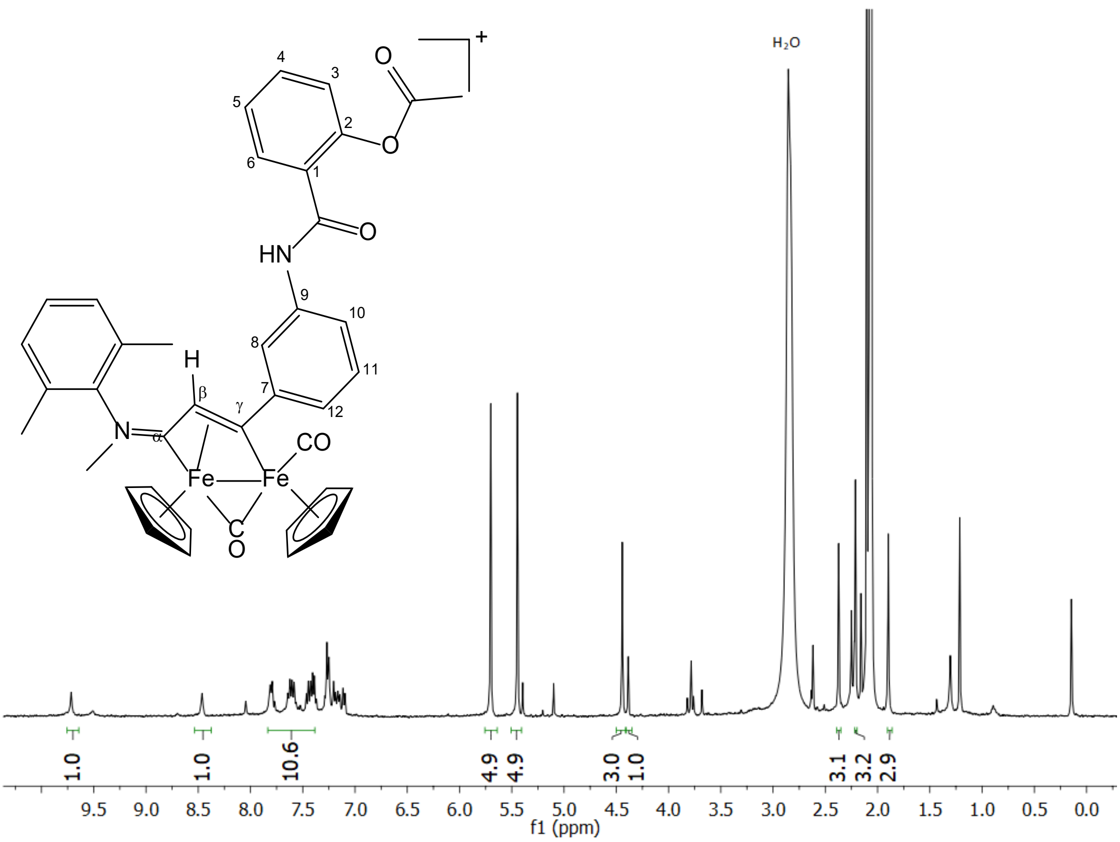

Figure S29. $^{13}C\{^{1}H\}$ NMR spectrum (101 MHz, acetone-d$_6$) of [**6**]CF_3SO_3.

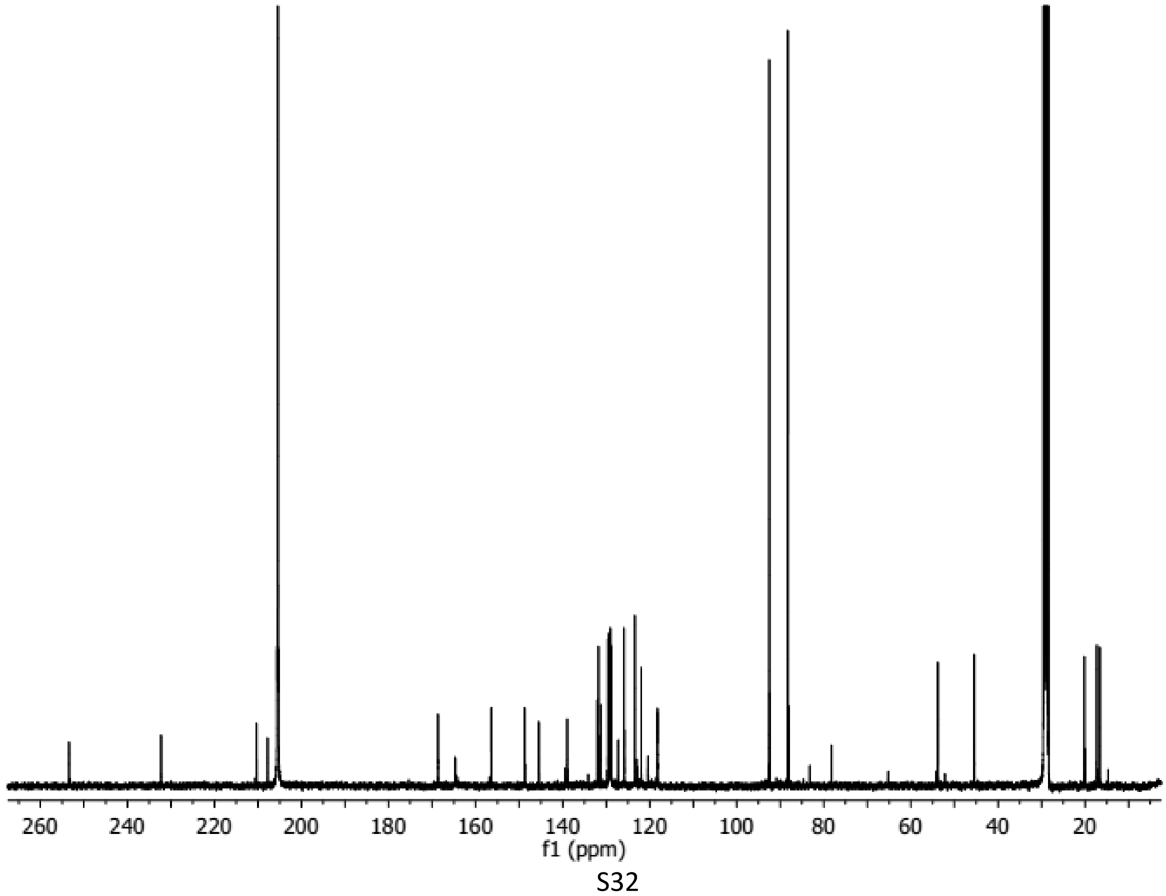

Figure S30. ^{1}H NMR spectrum (401 MHz, D_2O/DMSO) of [**6**]CF_3SO_3.

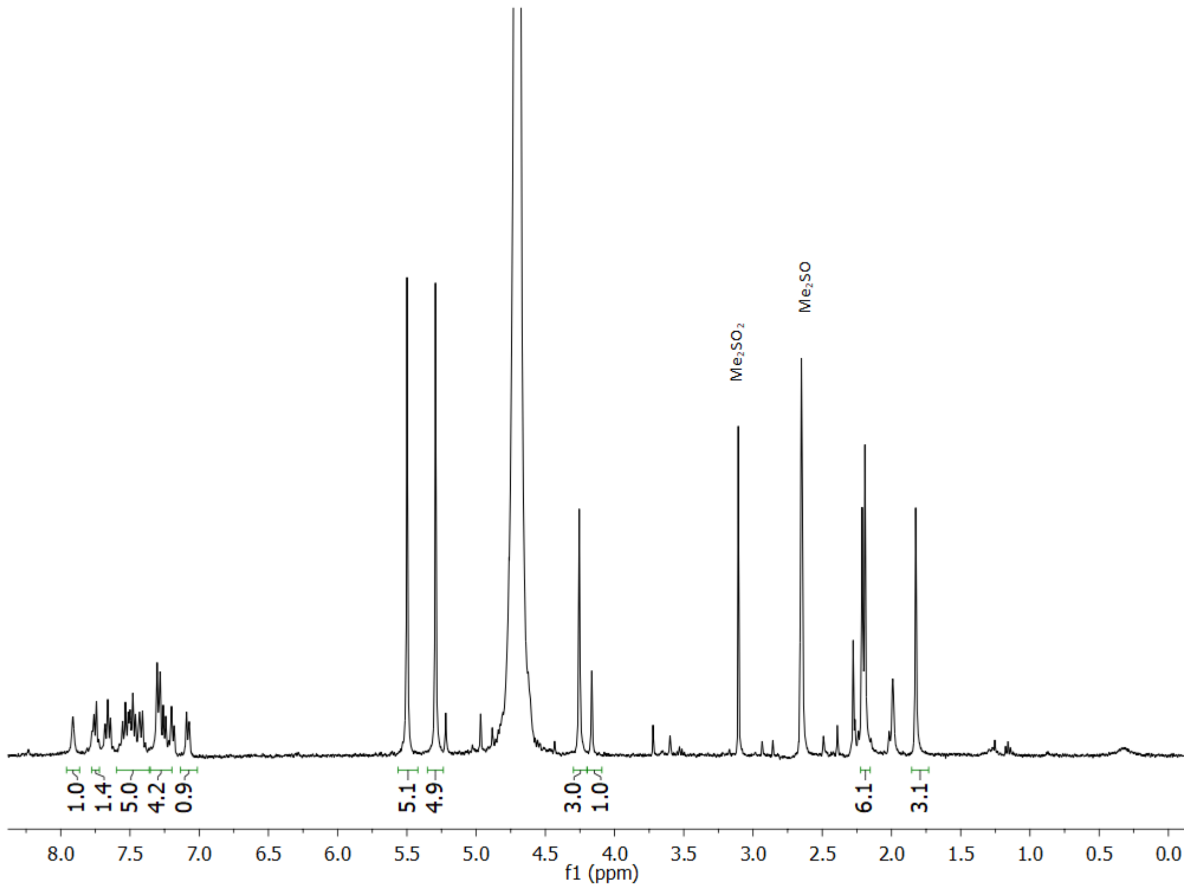

Figure S31. ^{1}H NMR spectrum (401 MHz, acetone-d$_6$) of [**7**]CF_3SO_3.

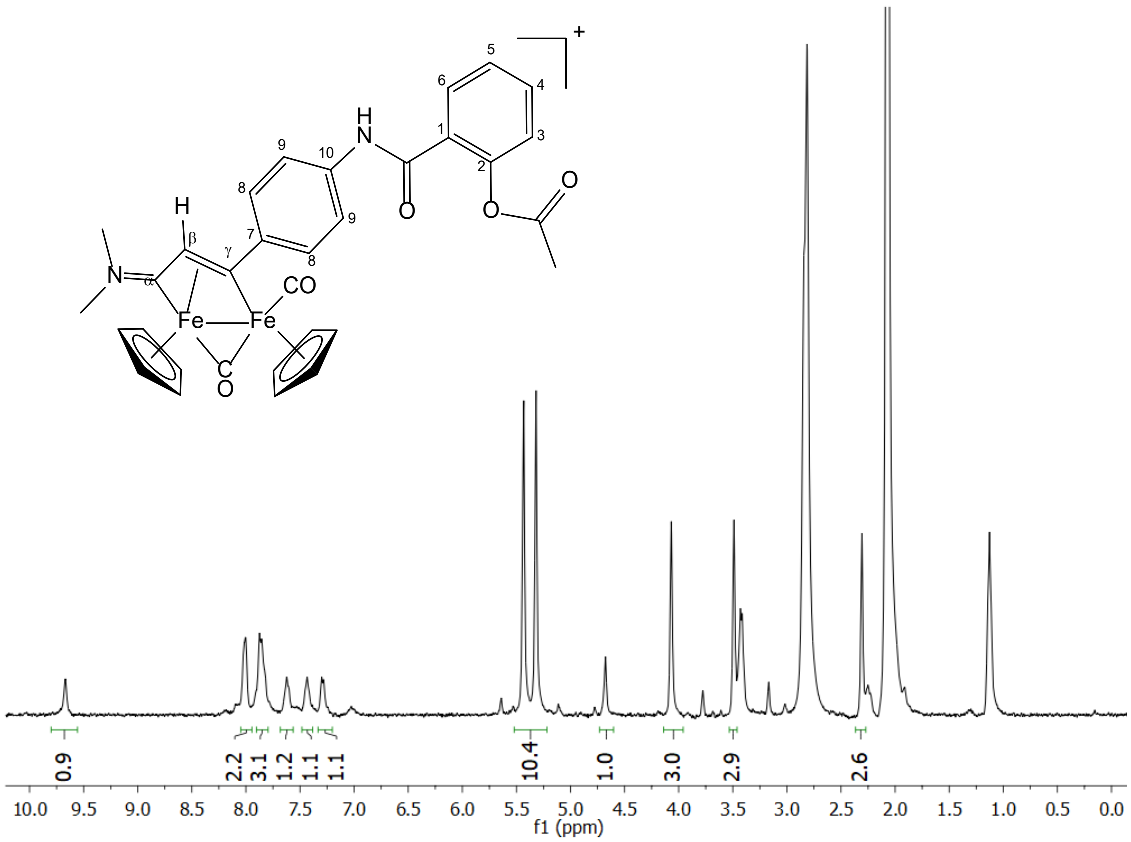

Figure S32. $^{13}C\{^{1}H\}$ NMR spectrum (101 MHz, acetone-d$_6$) of [**7**]CF_3SO_3.

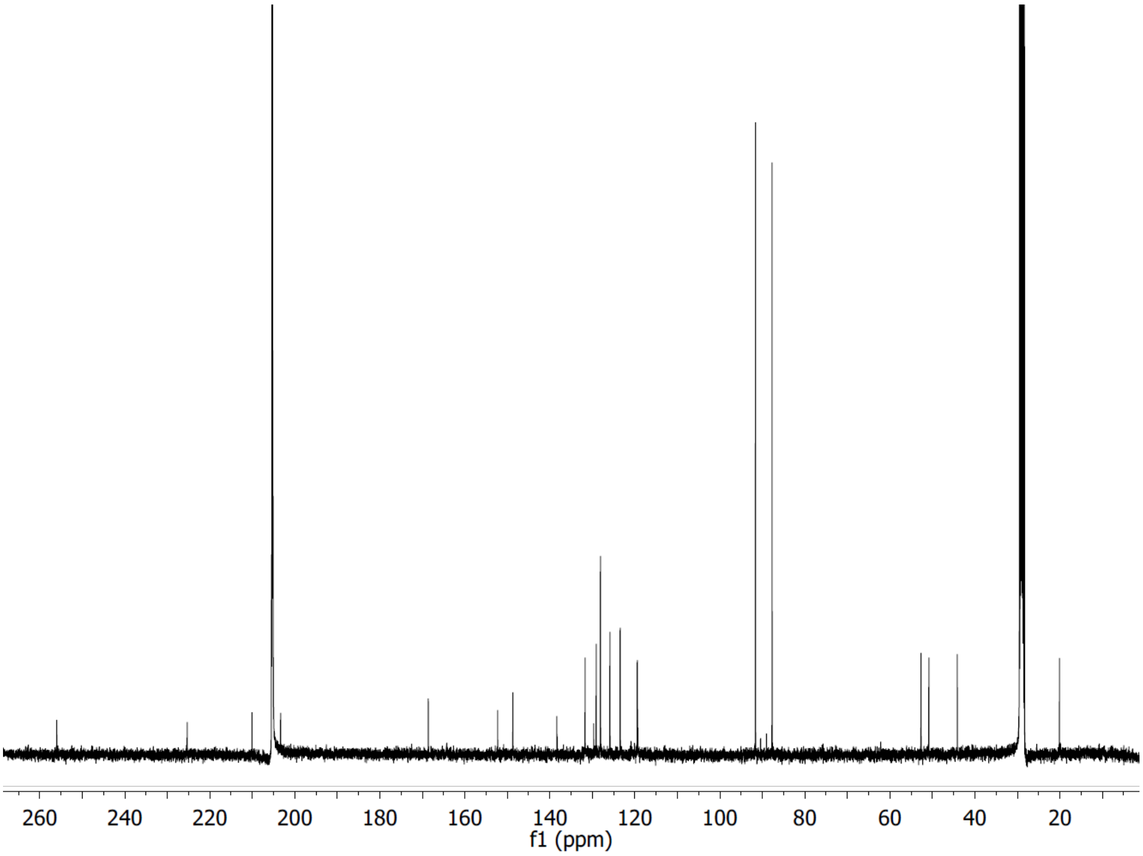

Figure S33. ^{1}H NMR spectrum (401 MHz, D_2O/DMSO) of [**7**]CF_3SO_3.

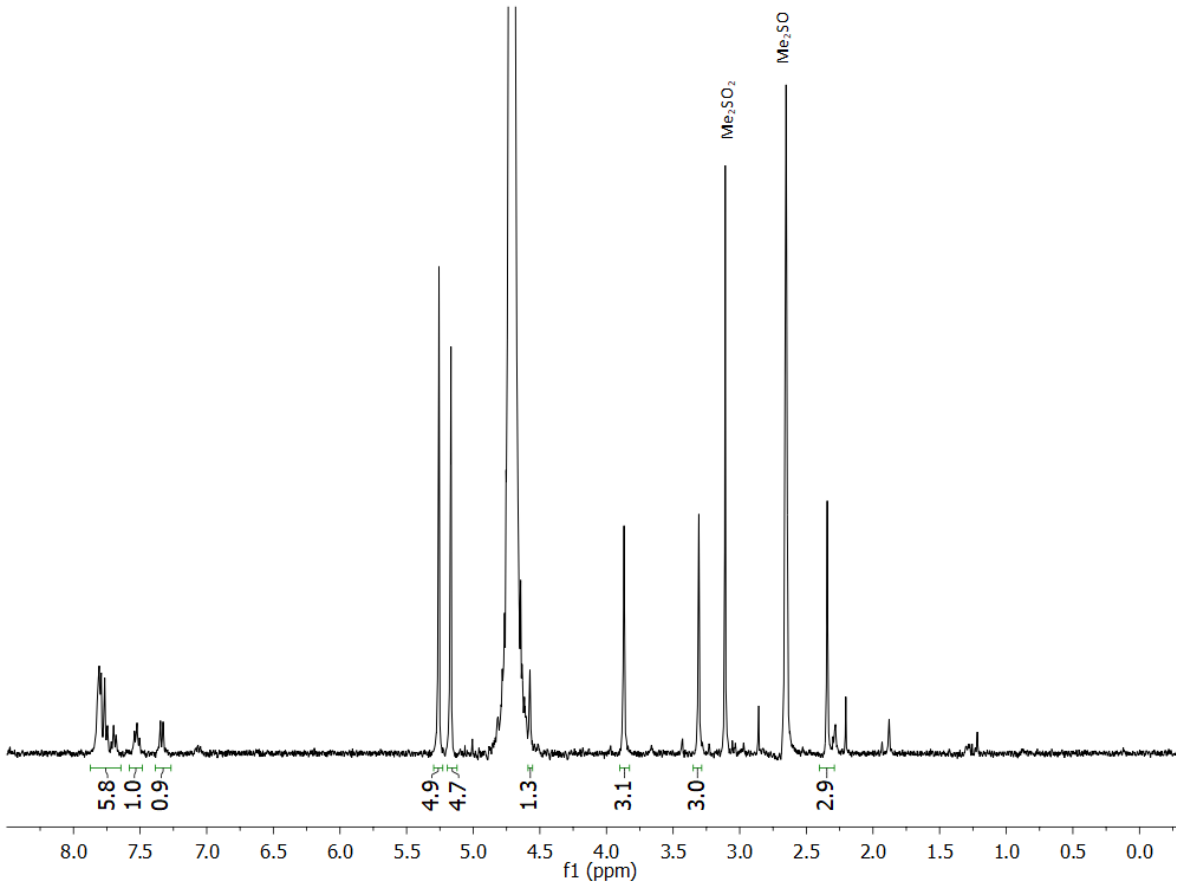

Figure S34. ^{1}H NMR spectrum (401 MHz, acetone-d$_6$) of [**8**]CF_3SO_3.

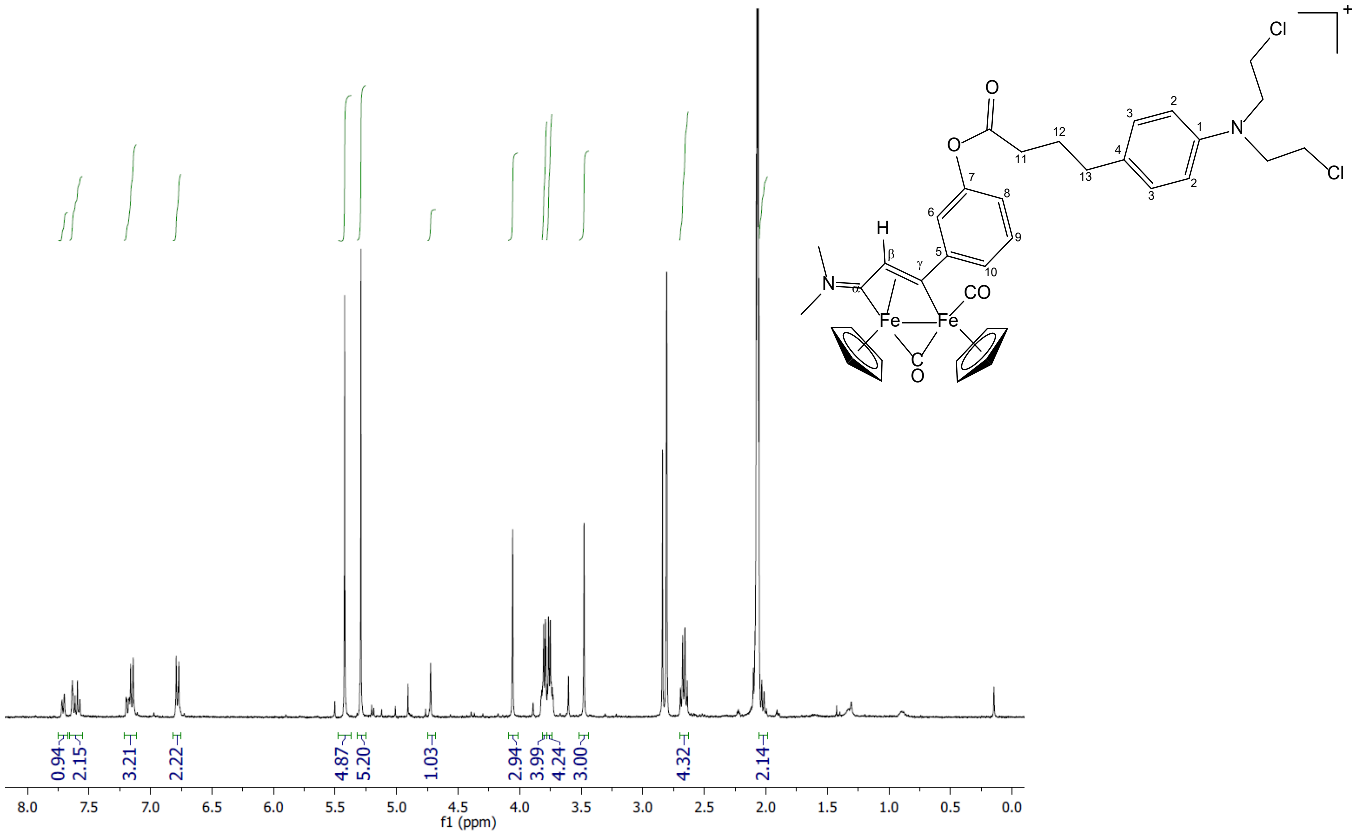

Figure S35. $^{13}C\{^{1}H\}$ NMR spectrum (101 MHz, $CDCl_3$) of [**8**]CF_3SO_3.

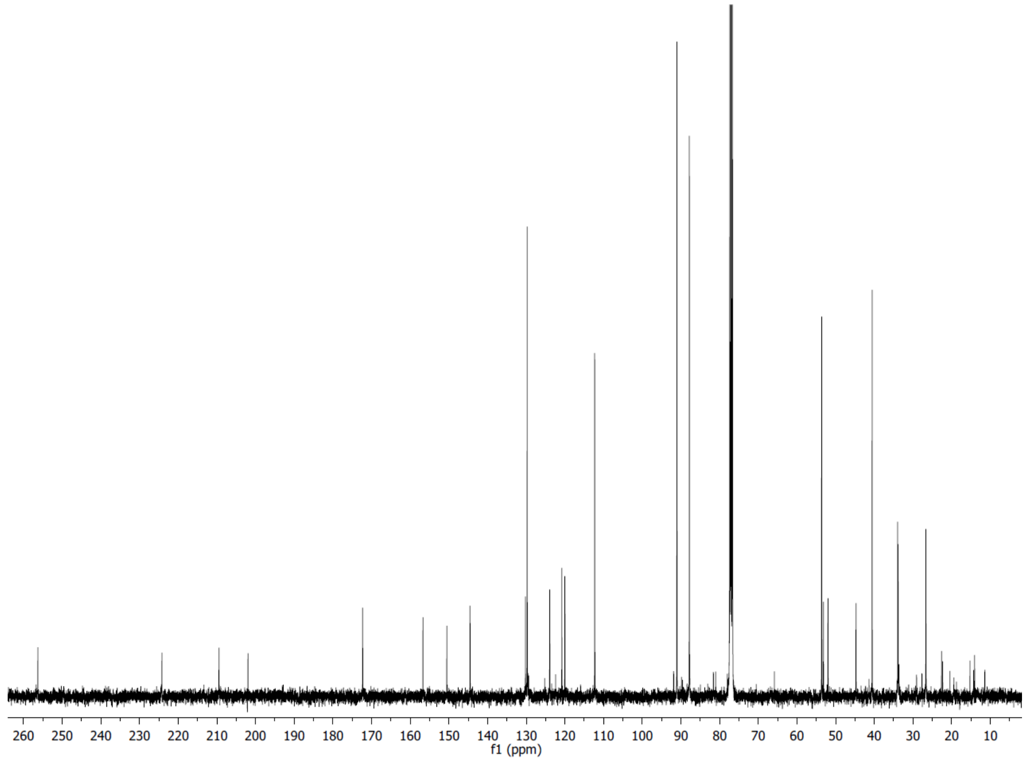

Figure S36. ^{1}H NMR spectrum (401 MHz, acetone-d_6) of [**9**]CF_3SO_3.

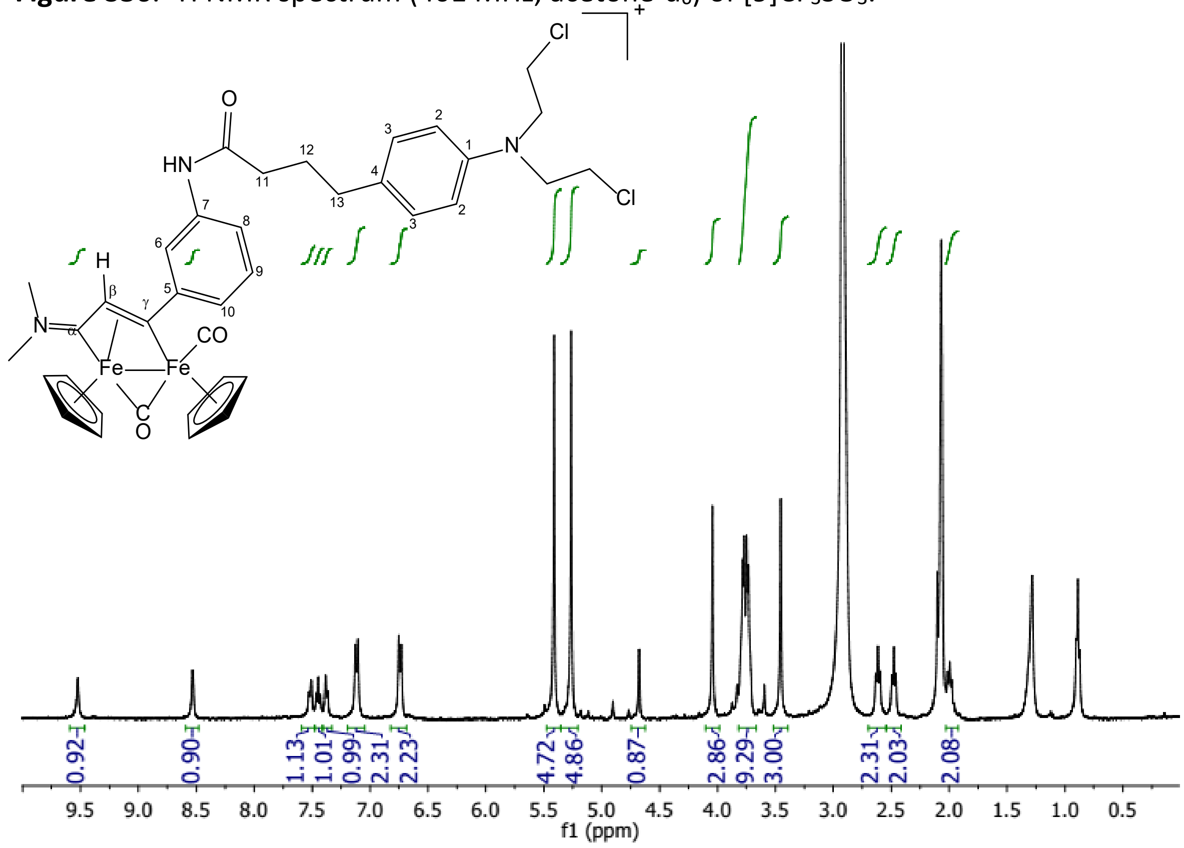

Figure S37. $^{13}C\{^{1}H\}$ NMR spectrum (101 MHz, acetone-d_6) of [**9**]CF_3SO_3.

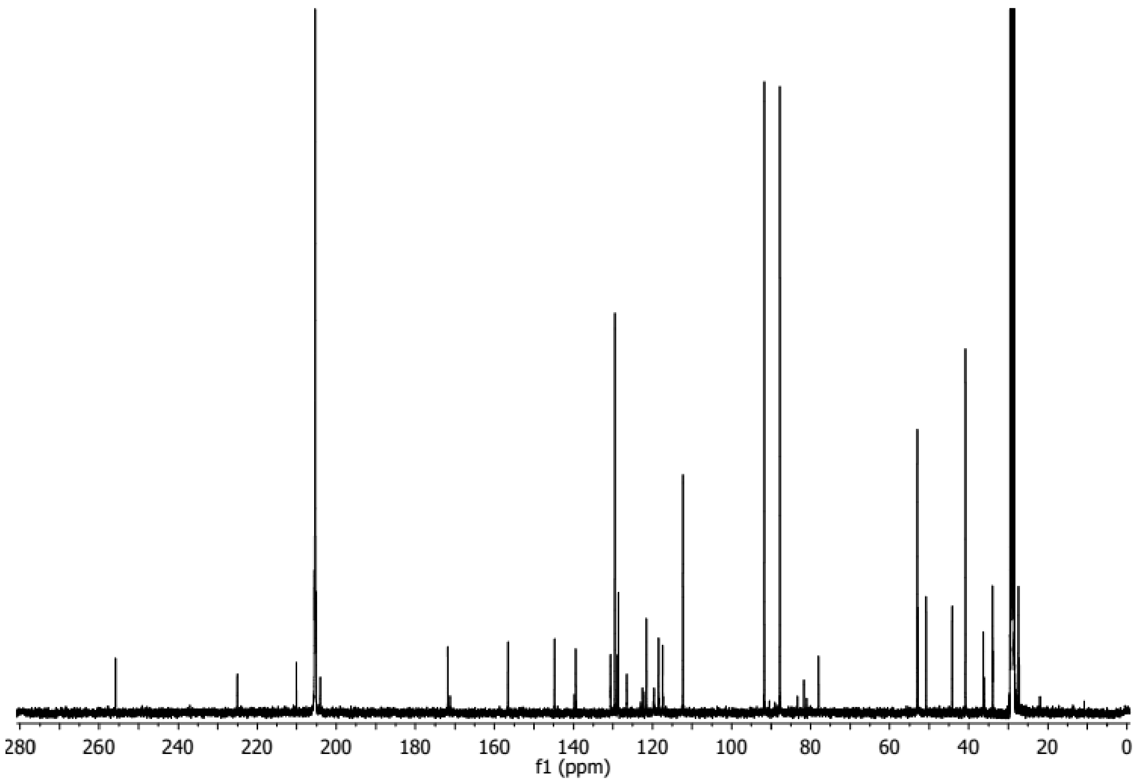

Figure S38. ^{1}H NMR spectrum (401 MHz, acetone-d$_6$) of [**10**]CF_3SO_3.

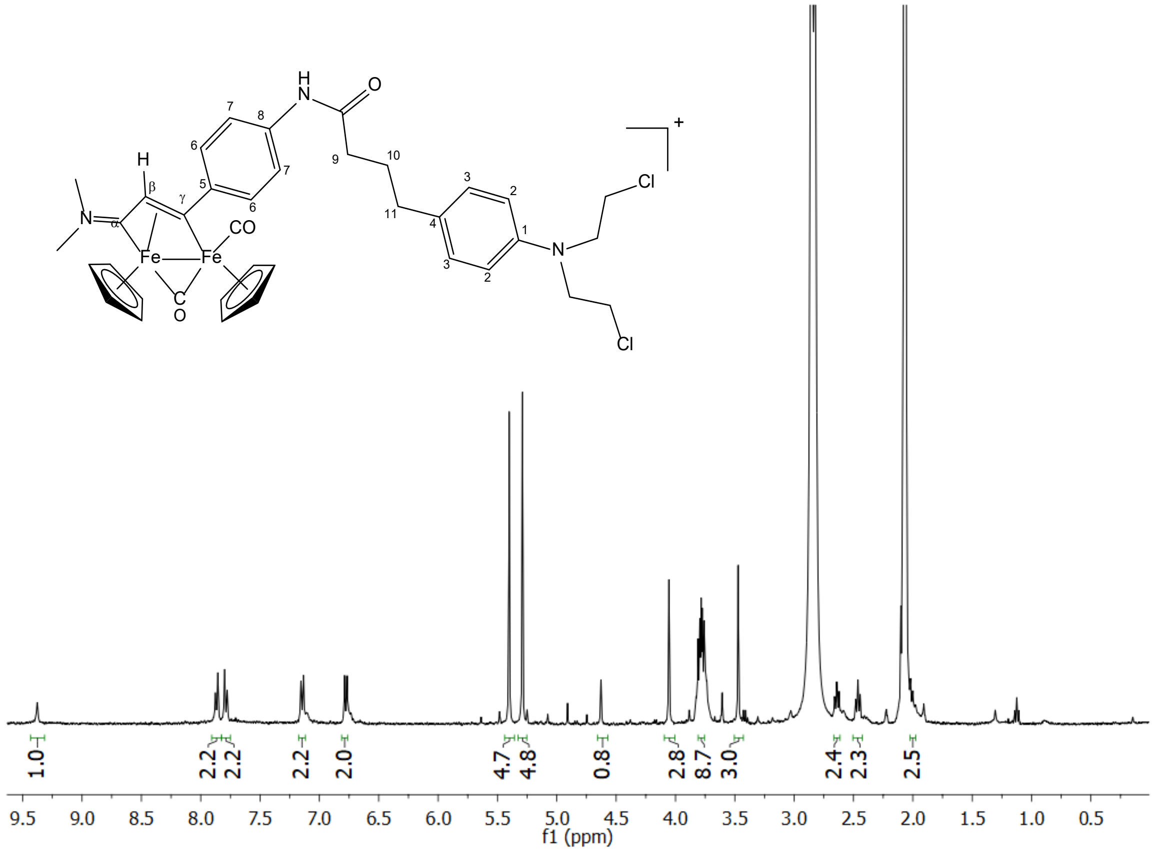

Figure S39. $^{13}C\{^{1}H\}$ NMR spectrum (101 MHz, acetone-d$_6$) of [**10**]CF_3SO_3.

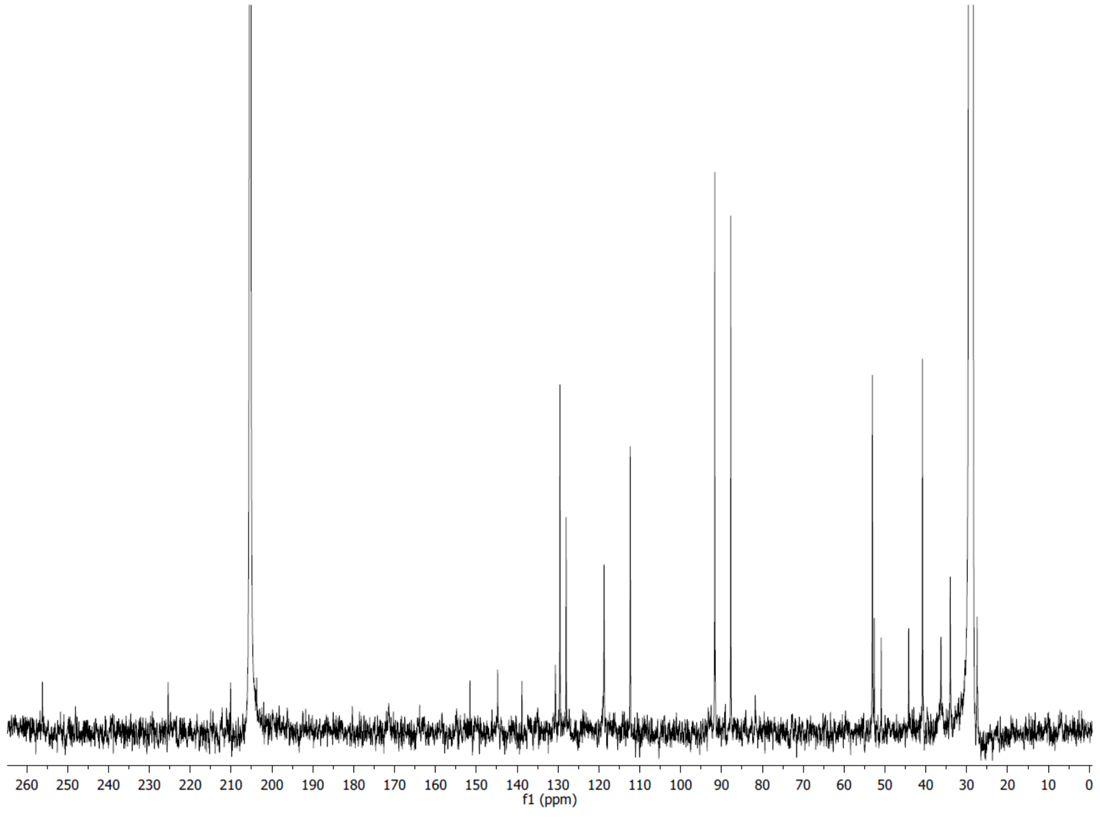

Figure S40. ^1H NMR spectrum (401 MHz, acetone-d$_6$) of [**2**]CF_3SO_3 after 24 hours in D_2O/DMSO 2:1 v/v mixture at 37 °C.

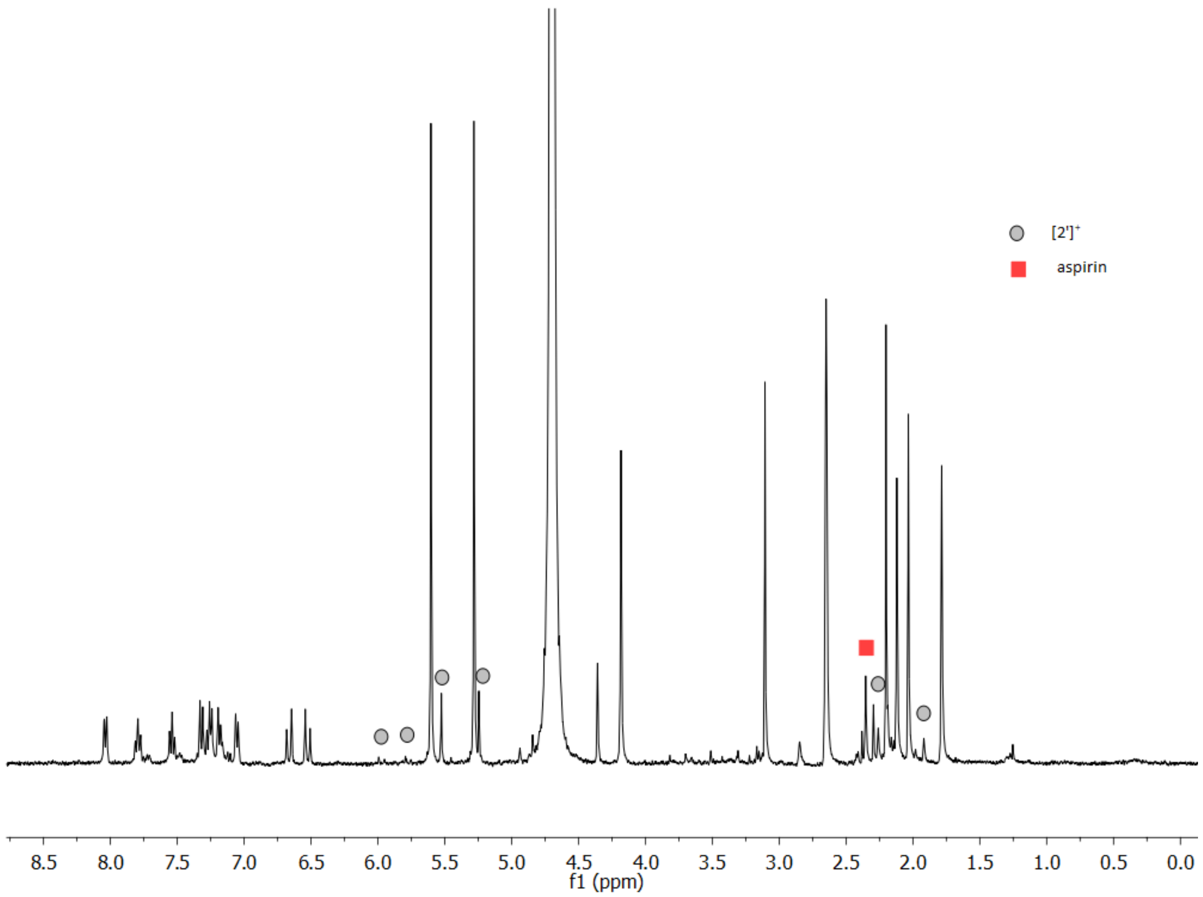

Figure S41. ^{1}H NMR spectrum (401 MHz, acetone-d_6) of [**3**]CF_3SO_3 after 72 hours in DMSO-d_6-DMEM at 37 °C.

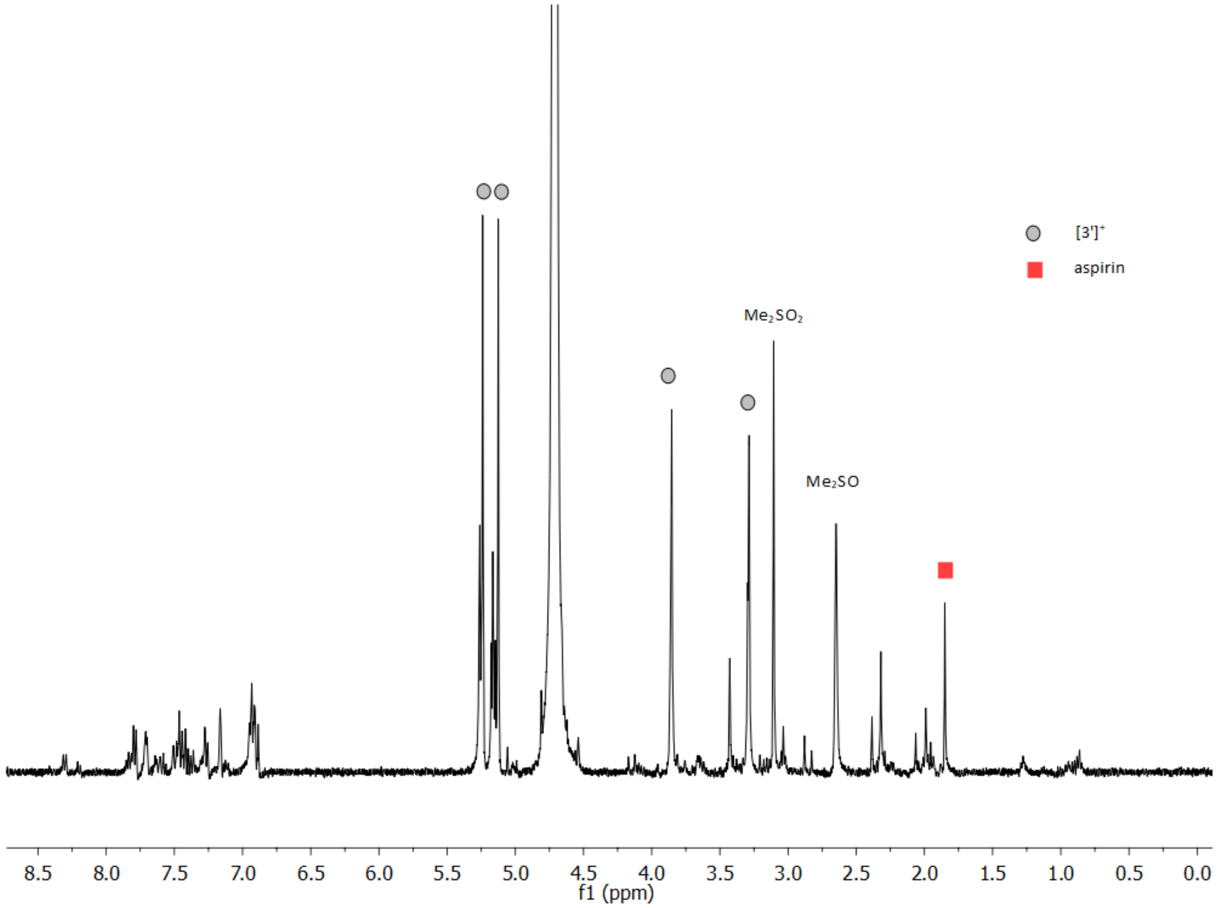

Figure S42. ^{1}H NMR spectrum (401 MHz, acetone-d_6) of [**5**]CF_3SO_3 after 72 hours in DMSO-d_6-DMEM at 37 °C.

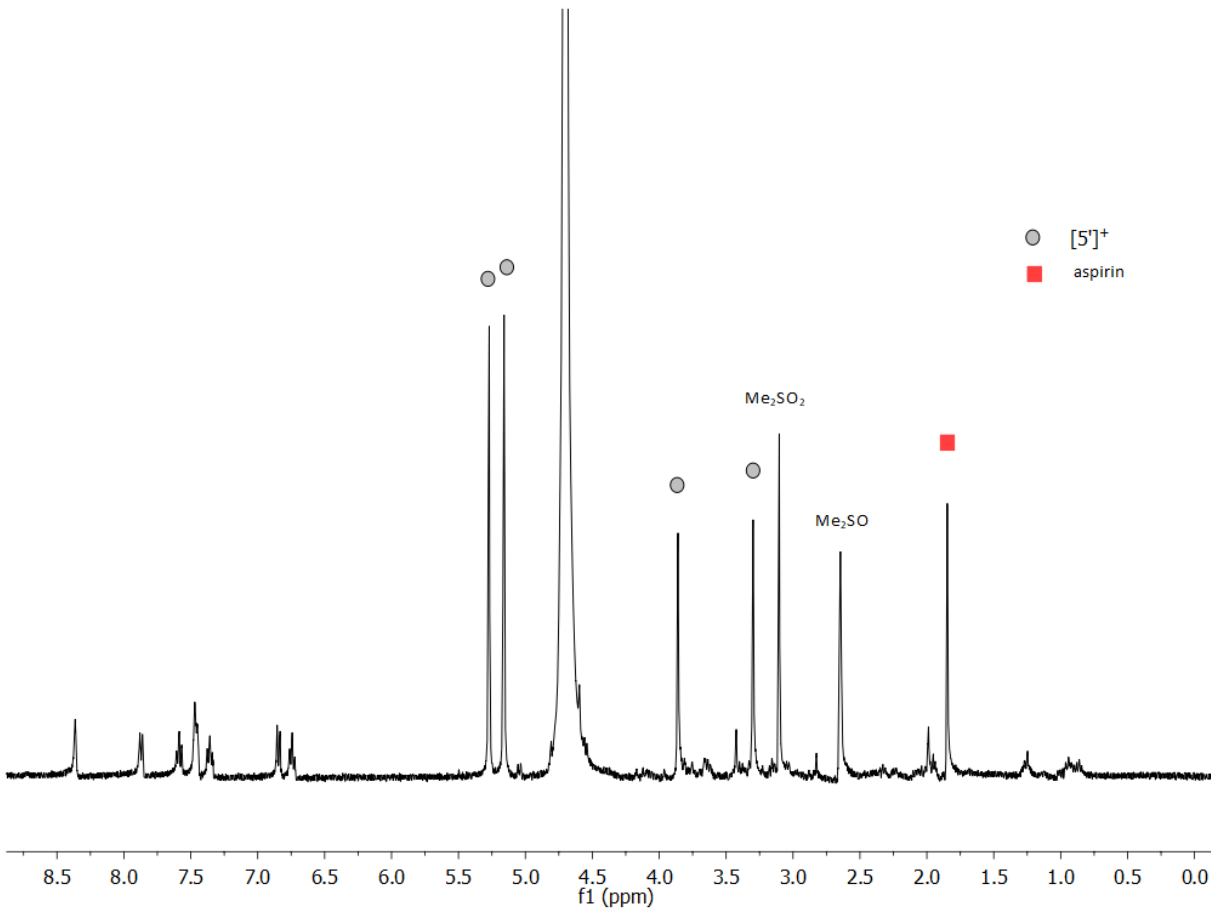

References

1 Rubner, G.; Bensdorf, K.; Wellner, A.; Kircher, B.; Bergemann, S.; Ott, I.; Gust, R. Synthesis and Biological Activities of Transition Metal Complexes Based on Acetylsalicylic Acid as Neo-Anticancer Agents. *J. Med. Chem*. **2010**, *53*, 6889–6898.

2 Biancalana, L.; Batchelor, L. K.; De Palo, A.; Zacchini, S.; Pampaloni, G.; Dyson, P. J.; Marchetti, F. A general strategy to add diversity to ruthenium arene complexes with bioactive organic compounds via a coordinated (4-hydroxyphenyl)diphenylphosphine ligand. *Dalton Trans*. **2017**, *46*, 12001-12004.

3 IR (CH_2Cl_2): ũ/cm^{-1} = 1796s (CO), 1675m, 1615m, 1597sh-w, 1519s.

4 Sheldrick, G. M. SADABS-2008/1 - Bruker AXS Area Detector Scaling and Absorption Correction, Bruker AXS: Madison, Wisconsin, USA, **2008**.

5 Sheldrick, G. M. Crystal structure refinement with SHELXL. *Acta Crystallogr.* **2015**, *71C*, 3-8.

6 (a) Spek, A. L. Single-crystal structure validation with the program PLATON. *J. Appl. Cryst.* **2003**, *36*, 7-13. (b) Spek, A. L. Structure validation in chemical crystallography. *Acta Cryst*. **2009**, *D65*, 148-155.

7 Rice, N. M.; Irving, H. M. N. H.; Leonard, M. A. Nomenclature For Liquid-Liquid Distribution (Solvent Extraction). *Pure & Appl. Chem*. **1993**, *65*, 2373-2396.

8 Biancalana, L.; Batchelor, L. K.; Funaioli, T.; Zacchini, S.; Bortoluzzi, M.; Pampaloni, G.; Dyson, P. J.; Marchetti, F. α-Diimines as Versatile, Derivatizable Ligands in Ruthenium(II) p-Cymene Anticancer Complexes. *Inorg. Chem*. **2018**, *57*, 6669-6685.

9 Rundlöf, T.; Mathiasson, M.; Bekiroglu, S.; Hakkarainen, B.; Bowden, T.; Arvidsson, T. Survey and qualification of internal standards for quantification by ^{1}H NMR spectroscopy. *J. Pharm. Biomed. Anal.* **2010**, *52*, 645-651.
